# Changes in ATP Sulfurylase Activity in Response to Altered Cyanobacteria Growth Conditions

**DOI:** 10.1264/jsme2.ME20145

**Published:** 2021-05-25

**Authors:** Lucia Gastoldi, Lewis M. Ward, Mayuko Nakagawa, Mario Giordano, Shawn E. McGlynn

**Affiliations:** 1 Laboratory of Algal and Plant Physiology, Department of Life and Environmental Sciences (DISVA), Università Politecnica delle Marche (UNIVPM), via Brecce Bianche, 60131 Ancona, Italy; 2 Department of Earth and Planetary Sciences, Harvard University, Cambridge, Massachusetts, USA; 3 Earth-Life Science Institute, Tokyo Institute of Technology, Ookayama, Tokyo, 152–8550, Japan

**Keywords:** ATPS, sulfur, *Synechocystis*, *Synechococcus*, proterozoic

## Abstract

We investigated variations in cell growth and ATP Sulfurylase (ATPS) activity when two cyanobacterial strains—*Synechocystis* sp. PCC6803 and *Synechococcus* sp. WH7803—were grown in conventional media, and media with low ammonium, low sulfate and a high CO_2_/low O_2_ atmosphere. In both organisms, a transition and adaptation to the reconstructed environmental media resulted in a decrease in ATPS activity. This variation appears to be decoupled from growth rate, suggesting the enzyme is not rate-limiting in S assimilation and raising questions about the role of ATPS redox regulation in cell physiology and throughout Earth history.

Sulfur is a universal and integral component of metabolism and biomass. Able to vary oxidation states from +6 to –‍2, and to populate the 3d orbitals, sulfur forms bonds with both carbon and iron, creating a bridge between the inorganic and organic in the cell (Beinert, 2000). With this redox and molecular versatility, sulfur is involved in diverse and unique metabolisms across the tree of life (Dahl and Friedrich, 2008). At the same time, it is found in conserved co-factors and biomass components such as S-adenosyl methionine (Marsh *et al.*, 2010; Bridwell-Rabb *et al.*, 2018), coenzyme A (Strauss, 2010), and proteogenic cysteine residues, with their attendant in Fe-S clusters (Beinert *et al.*, 1997; Rouault, 2019; Gao, 2020). With these properties, sulfur involving reactions likely had a prominent role from the origin of life onward (Wächtershäuser, 1990; De Duve and De Neufville, 1991; Russell *et al.*, 1994; Goldford *et al.*, 2017).

Much later in evolution, sulfur availability in the oxidized form of sulfate may have influenced oceanic phytoplankton primary productivity (Norici *et al.*, 2005; Giordano and Prioretti, 2016), and a considerable literature exists describing sulfur in the metabolism, ecology, and evolution of this group (Kopriva and Rennenberg, 2004; Giordano *et al.*, 2005, 2008; Ratti *et al.*, 2011; Takahashi *et al.*, 2011; Giordano and Prioretti, 2016). Prominent areas of discussion are the sulfonium compound metabolism of DMSP/DMS (Giordano *et al.*, 2005; Ratti and Giordano, 2008; Takahashi *et al.*, 2011; Giordano and Prioretti, 2016), protein Fe-S cluster biosynthesis (Rina *et al.*, 2000; Cassier-Chauvat and Chauvat, 2014; Gao, 2020), and sulfur acquisition required for the thylakoid membrane in chloroplasts (Ratti *et al.*, 2011; Goss and Wilhelm, 2009). Chemical data about different phytoplankton species underline how more ancient cyanobacteria and green algae have a higher C:S ratio than the diatoms, dinoflagellates and coccolithophores which dominate ocean water today, and it has been hypothesized that the historical increases in sulfate availability in the water column allowed the spread of S requiring species. Specifically, Giordano’s laboratory and collaborators (Ratti *et al.*, 2011; Prioretti and Giordano, 2016) suggested that variations in sulfate availability may have been an evolutionary constraint in the phytoplankton radiation (Ratti *et al.*, 2011; Prioretti and Giordano, 2016). Going further through time, sulfate variability in the ocean may also have recorded a linkage between animal evolution and the geochemical record of sulfate deposits derived from the stirring action of benthonic organisms (Canfield and Farquhar, 2009).

In unicellular algae and cyanobacteria (and many other organisms), S acquisition from the environment into biomass begins from sulfate (Giordano and Prioretti, 2016). Sulfate is kinetically inert and requires activation, which is then followed by reduction to biomass appropriate oxidation states (Fig. 1). ATP Sulfurylase (ATPS—EC 2.7.7.4) has the key role of SO_4_^2–^ activation at the beginning of the S assimilation pathway, hydrolyzing ATP and producing a sulfate-ester (Schmidt, 1972, 1988; Ullrich *et al.*, 2001; Takahashi *et al.*, 2011; Prioretti *et al.*, 2014; Giordano and Prioretti, 2016).

In plants, S assimilation is regulated at different levels in response to growth conditions and in accordance with C and N metabolisms (Vauclare *et al.*, 2002; Kopriva and Rennenberg, 2004; Martin *et al.*, 2005; Khan *et al.*, 2010; Takahashi *et al.*, 2011; Koprivova and Kopriva, 2014). In both algae and cyanobacteria, diverse regulatory mechanisms exist (Schmidt, 1988; Takahashi *et al.*, 2011) and have been investigated (MacRae *et al.*, 2001; Kopriva *et al.*, 2002; Patron *et al.*, 2008; Prioretti *et al.*, 2014). At the stage of sulfate activation, redox regulation of ATPS enzyme activity was postulated and subsequently confirmed (Prioretti *et al.*, 2014, 2016). Within cyanobacteria, the redox regulated isoform, ATPS–B, contains 5 conserved cysteine residues (Prioretti *et al.*, 2014), whereas ATPS–A, with only 4 conserved cysteine residues (Prioretti *et al.*, 2014), appears to lack the critical regulatory S-residue organization thought to allow disulfide bridge formation and the modulation of enzyme activity.

It has been hypothesized that freshwater and marine cyanobacteria species are characterized by different evolutionary histories (Sánchez-Baracaldo, 2015; Sánchez-Baracaldo *et al.*, 2017). Consequently, the different environments in which they evolved (perhaps including less or more oxidizing) could have influenced the presence/absence of redox regulation in specific proteins. The phylogenetic distribution pattern of ATPS homologs (Fig. 1B—see Supplemental Material for the explanation of how the tree was constructed) is suggestive of this relationship. The tree shows how the marine photosynthetic Syn/Pro clade (*Synechococcus*, *Prochlorococcus* and *Cyanobium*—Sánchez-Baracaldo, 2015) clade is well separated from other photosynthetic cyanobacteria (including the freshwater group). Further highlighting functional gene acquisition into the cyanobacteria phylum, the Vampirovibrionia class,—which represents an ancient and non-photosynthetic cyanobacteria taxon (Soo *et al.*, 2019)—is found on a different branch of the tree (Fig. 1B). Within freshwater cyanobacteria species (and those marine species not enclosed in the Syn/Pro clade) the ATPS–A isoform without redox regulation is found, while, in the more derived Syn/Pro clade (which constitutes the picocyanobacteria plankton) the ATPS–B isoform with redox-regulation is found (Prioretti *et al.*, 2014, 2016; Giordano and Prioretti, 2016). This pattern of distribution also corresponds to ribulose 1,5-bisphosphate carboxylase/oxygenase (RubisCO), and carboxysomes (Badger and Price, 2003; Rae *et al.*, 2013) distribution patterns: cyanobacteria with ATPS–A match the freshwater and brackish β-cyanobacteria which possess RubisCO-1B and β-carboxysomes; while those with ATPS–B coincide with the marine α-cyanobacteria that have RubisCO-1A and α-carboxysomes (Prioretti *et al.*, 2016).

Protein phylogenies can be linked with gene transfer events during evolution, and several studies indeed confirmed the importance of horizontal gene transfer (HGT) during the evolution of the oxygenic photosynthesis in the cyanobacteria (Fischer *et al.*, 2016; Hohmann-Marriott and Blankenship, 2011). It has been observed for example that the Syn/Pro clade seems to have acquired a large number of genes via HGT from Proteobacteria: several genes involved in the formation of the α–carboxysome have been transferred from this group to cyanobacteria along with bacteriochlorophylls synthesis genes (Bryant *et al.*, 2012; Ward and Shih, 2021). The ATPS phylogeny (where Proteobacterial sequences are more numerous and more widely distributed across the tree) is consistent with the theory that the ATPS gene was part of this exchange (Fig. 1B and Supplemental Materials), with the ATPS–B sequences nested within a clade primarily made up of proteobacterial sequences and very distant from the ATPS–A clade. Moreover, a small number of Vampirovibrionia species having a different version of ATPS protein is consistent with them acquiring it via HGT after their divergence from oxygenic cyanobacteria, similar to how they acquired those proteins involved in aerobic respiration (Soo *et al.*, 2017, 2019).

To further our understandings of the ATPS protein and its regulation in cyanobacteria, we grew two cyanobacteria species: the freshwater *Synechocystis* sp. PCC6803 (referred to simply as *Synechocystis* from now on) with non-redox regulated ATPS–A and, and the marine *Synechococcus* sp. WH7803 (referred to as *Synechococcus* from now on) with redox-regulated ATPS–B in multiple growth conditions and measured the resulting enzyme activity with a crude cell extract assay. The experiments allowed a comparison of enzyme activity levels between the two isoform types when expressed in cells exposed (and adapted) to the modern environment and a possible Precambrian condition. In particular, we considered the Proterozoic Eon, which lasted from 2.5 Gyr to 0.6/0.5 Gyr ago (refer to the following for more description: Fischer, 2008; Rasmussen *et al.*, 2008; Lyons *et al.*, 2014; Knoll and Nowak, 2017) and included the oxygenation of Earth’s atmosphere (Lyons *et al.*, 2014). During this period is when most evolutionary theories place the differentiation and radiation of the cyanobacteria taxon (Sánchez-Baracaldo, 2015; Schirrmeister *et al.*, 2016; Shih *et al.*, 2017)—despite most of the extant diversity of this taxon having been accumulated during the Phanerozoic Eon (Louca *et al.*, 2018).

For each species, three experimental conditions (with three biological replicates each) were set up in a growth chamber (12 h light/12 h dark cycle, temperature 20°C and light with a white LED lamp at 50‍ ‍μmol photon m^–2^ s^–1^ of irradiance). The cells were grown as semi-continuous cultures with the dilution volume based on growth rate data (Gastoldi *et al.*, unpublished) The three conditions analyzed were: Standard Condition (ST—Table S1), the Possible Proterozoic Condition (PPr—Table S2) and the Transitional Condition (TR—Table S3). AMCONA medium (Fanesi *et al.*, 2014) was used for the marine *Synechococcus* species while the BG11 medium (Stanier *et al.*, 1971) was used for the freshwater *Synechocystis*. All the liquid cultures were bubbled continuously with the atmosphere corresponding to the specific condition and experiments were performed after an adaptation period (2/3 months). The PPr condition had a higher CO_2_/O_2_ ratio than today (10,000 ppm of CO_2_ ensured by a controlled gas system) while the TR and PPr growth media had a lower sulfate concentration, a switch from nitrate to ammonium, and, in the case of the *Synechococcus*, lowered Fe concentrations compared to the ST. TR and PPr were identical except for the gas composition, with TR using air and PPr bubbled with a CO_2_ mix (details available in Supplemental Materials). The media compositions were derived from literature survey and consideration of each strain’s standard media. While the modified media might capture some variability with historical relevance, this can and should be debated.

Growth curves were determined as explained in other works (Gastoldi *et al.*, unpublished). ATPS activity was measured using crude cell extracts (refer to Supplemental Materials for a detailed procedure—which followed Giordano *et al.*, 2000): the activity was observed spectrophotometrically at 25°C for 15‍ ‍min and the linear phase of the assay was considered for the data analyses, as in previous works (Burnell, 1984; Giordano *et al.*, 2000; Prioretti *et al.*, 2016). The specific activity of ATPS in the crude extract was then normalized to the concentration of protein (expressed in *mg mL*^–^*^1^*) of the extract itself which was determined through the Lowry/Peterson technique (Lowry *et al.*, 1951; Peterson, 1977).

For both organisms, the specific activity of the ATPS enzyme in the cells was very different between conditions. In *Synechocystis*, the mean activity value was 1459‍ ‍nmol·min^–1^·mg^–1^ in the ST condition, higher than in the TR condition (164‍ ‍nmol·min^–1^·mg^–1^—ANOVA, *P-value*=0.0007, Post hoc: Tukey’s t-test, STvsTR, *P-value*=0.0006; STvsPPr, *P-value*=0.0067; TRvsPPr, *P-value*=0.0717, *n*=9, Fig. 2), where S and Fe were lowered in concentration compared to the standard media and N changed from nitrate to ammonium. The TR condition also showed a lower value than the PPr condition, where the value was 634‍ ‍nmol·min^–1^·mg^–1^, though the difference between TR and PPr was less significant judged by a Tukey test (used as post hoc) which gave a higher *P-value* (*P-value*=0.0717).

In *Synechococcus*, the average value was 2689‍ ‍nmol min^–1^ mg^–1^ in the ST condition while in the TR condition was only 32‍ ‍nmol·min^–1^·mg^–1^ (Welch’s t-Test, *P-value*=0.052, *n*=6). The experiment was not possible for the seawater PPr condition since the *Synechococcus* species was not able to survive in that condition despite several attempts for its adaptation.

In both *Synechocystis* and *Synechococcus*, the difference between the ATP sulfurylase activity in the ST and the TR conditions points out that media components other than supplied gas can have strong effects on the enzyme activity and growth, since the atmosphere was the same in both conditions. It may be that, in these organisms, a limitation in more than one nutrient (in our work we lowered sulfate and switched from nitrate to ammonium between ST and TR conditions), could decrease ATPS expression. This result is contrary to previous results where sulfate alone was varied and the activity increased in *Synechococcus* sp. WH7803 (Prioretti and Giordano, 2016). Further work is needed to address the factors that regulate ATPS, but we can now see that nutrients supplied as dissolved salts can strongly affect the enzyme specific activity.

Plotting enzyme activity vs. growth rate for both the organisms, a trend between growth rate and ATPS activity was not observed, suggesting that the enzyme activity itself is not limiting growth in these conditions (Fig. 2B). In the marine *Synechococcus*, the growth rate in the low sulfate TR condition was higher than the ST condition, but the ATPS enzyme activity was much lower. In the case of *Synechocystis*, which could grow in the PPr condition and does not have a redox regulated ATPS (Prioretti *et al.*, 2016), a higher value of ATPS activity was found in the PPr condition than in the TR condition, despite a lower growth rate value in the PPr condition (Fig. 2B). One hypothesis stemming from this observation is that the lower O_2_ available, which occurred in the PPr environment, promotes the activity of the enzyme or its expression. Although still preliminary, more data of this type, together with sulfur quota data which can be related with growth rates for some algae (Prioretti and Giordano, 2016), will aid in assessing the relationships between ATPS enzyme activity, growth rate, and sulfur cell content. It is curious that in *Synechococcus*, while the enzyme activity decreased the S content of the cells increased (Gastoldi *et al.*, unpublished). This could again imply that the enzyme is not limiting in the uptake of S. Together, our results pose questions as to why the enzyme is redox regulated in some organisms, and also why the activity varies so greatly between conditions.

We analyzed the phylogenetic distribution, and regulation of enzyme activity at the first step in ATP and electron requiring sulfate assimilation. By studying two organisms—one with redox regulation at the ATPS step and one without, our motivation was to gain insight into intra-cell redox responses in relationship to environmental redox. Our results highlight that cyanobacterial lineages display unique phenotypes in these conditions. This variability adds richness—and some complication—to theories about cyanobacteria evolution and adaptation to the oxygenation of the planet, as other works started recently to point out (Herrmann *et al.*, 2020; Uchiyama *et al.*, 2020). A long term goal is to investigate the possible linkages between metabolic regulation and Earth history.

**Funding**: L. Gastoldi received a Ph.D. scholarship from the Univerità Politecnica della Marche (UNIVPM), and the research was additionally supported by the ELSI RIC. L. M. Ward was supported by a Simons Foundation Postdoctoral Fellowship in Marine Microbial Ecology. S.E.M. acknowledges support from JSPS KAKENHI (Grant No. JP18H01325). M.N. was supported by JSPS KAKENHI grants JP17K14412 and JP17H06105.

## Citation

Gastoldi, L., Ward, L. M.., Nakagawa, M., Giordano, M., and McGlynn, S. E.. (2021) Changes in ATP Sulfurylase Activity in Response to Altered Cyanobacteria Growth Conditions. *Microbes Environ ***36**: ME20145.

https://doi.org/10.1264/jsme2.ME20145

## Supplementary Material

Supplementary Material 1

Supplementary Material 2

Supplementary Material 3

## Figures and Tables

**Fig. 1.**
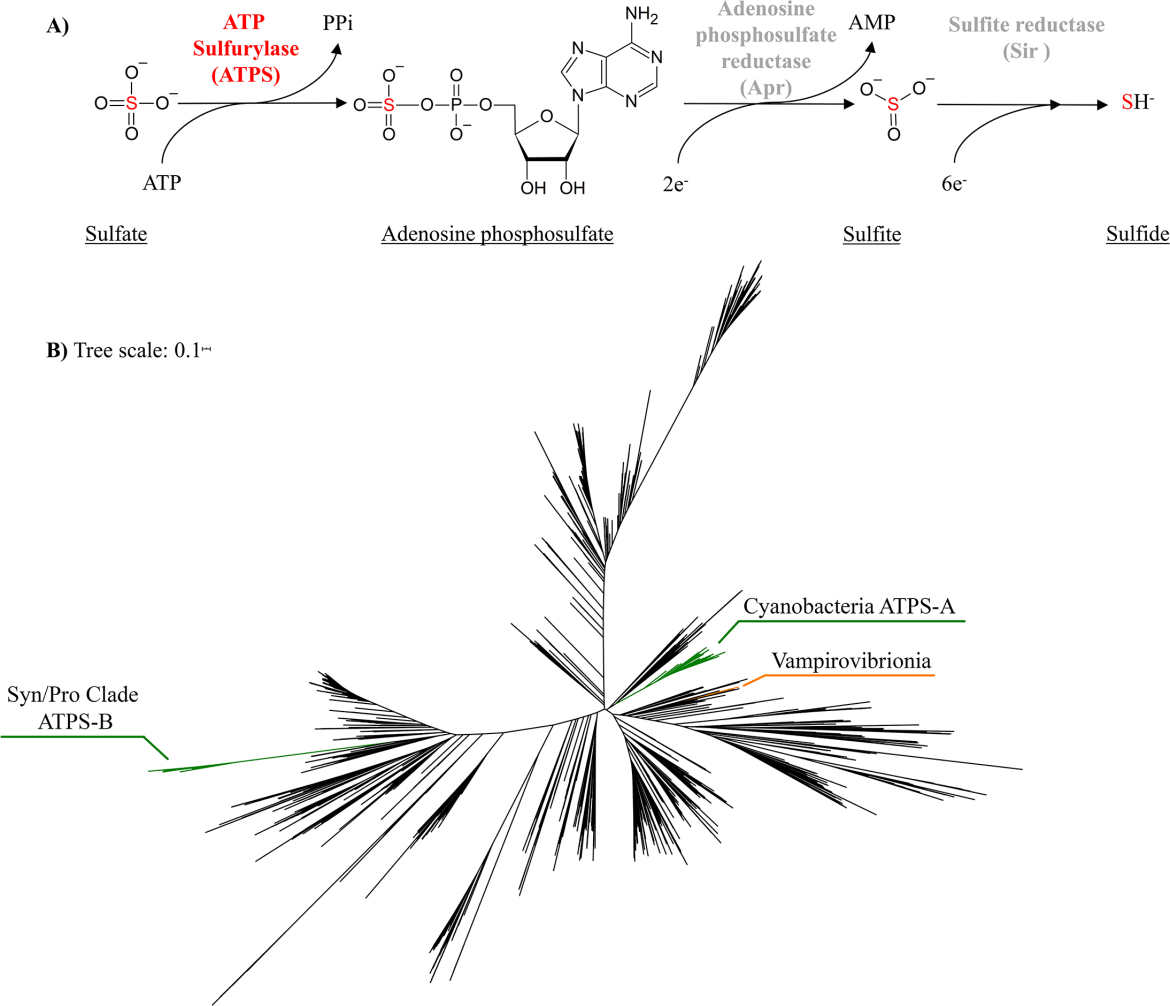
**A)** The role of ATP Sulfurylase in sulfate activation and the downstream steps leading to sulfide. The two step PAPS path can also reduce adenosine phosphosulfate to sulfite but is not shown. **B)** Phylogeny of ATP sulfurylase in bacteria and archaea. Homologs from the Vampirovibrionia (an ancestral non-photosynthetic cyanobacteria class) are in orange, while the oxygenic cyanobacteria are labelled in green as the Syn/Pro clade with the ATPS-B isoform, and those cyanobacteria with the ATPS-A isoform. Remaining archaea and bacteria are colored in black. The tree can be found as a file in the Supplemental Materials.

**Fig. 2.**
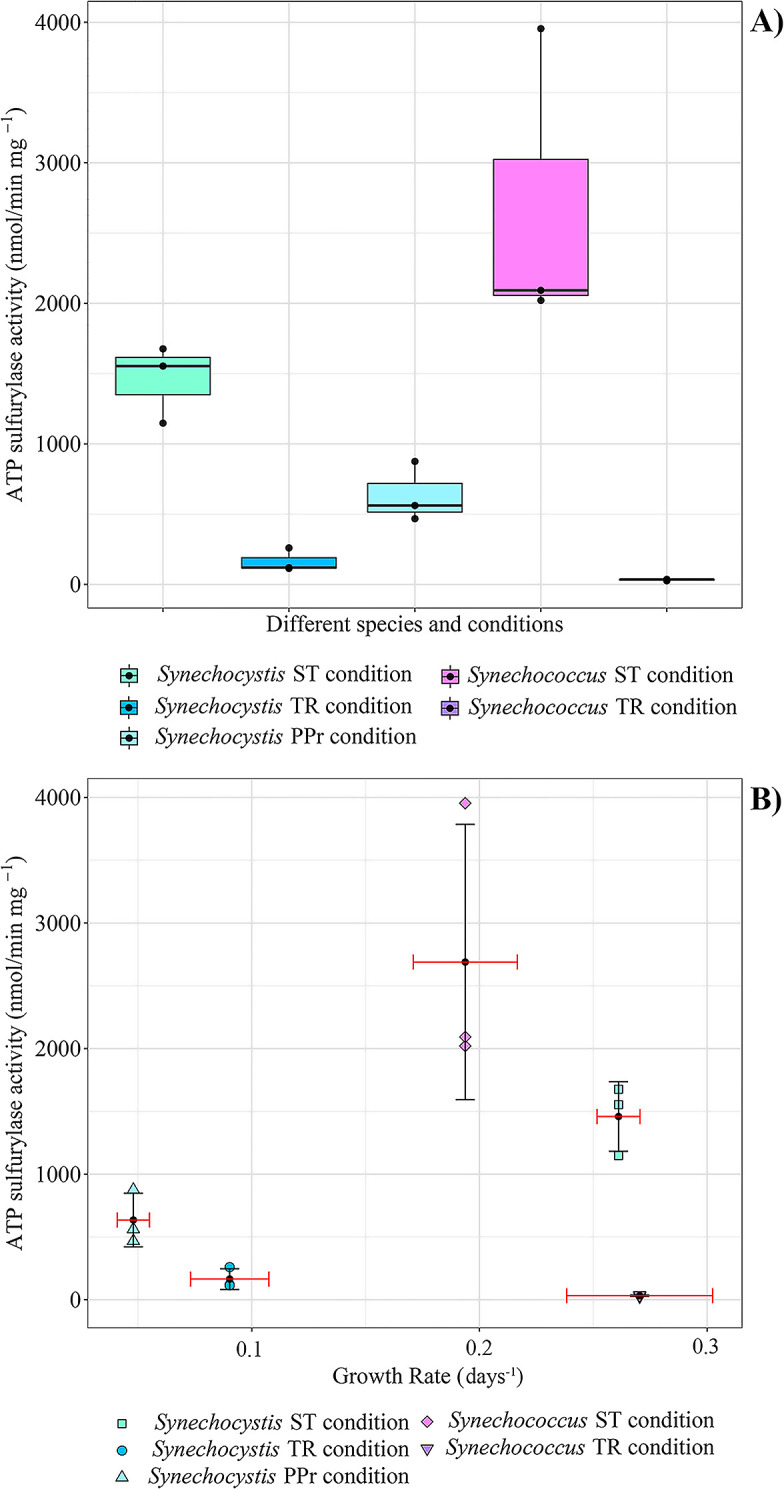
ATP sulfurylase activity. **A)** Each box represents the interquartile range of a specific condition and the black dots are the biological replicates for each one: the black line is the median and represents the second quartile Q2. Above the line is the upper quartile Q3 while below the line is the lower quartile Q1. The lines that come out from each box represent the minimum (lower) and the maximum (higher) value in the data. **B)** Each point represents a biological replicate of ATPS activity measurement; each condition is characterized with different shape and color. The black dot in each set of values is the mean of both the ATPS activity and the Growth Rate for the specific dataset. For each mean a Standard Deviation (SD) was also added, black SD for the ATPS activity value and red SD for the Growth Rate value. Single values used for this figure can be found in Table S4 in the Supplemental Materials.
