# Supplementary material for: Changes in ATP Sulfurylase Activity in Response to Altered Cyanobacteria Growth Conditions
Source: Microbes Environ. 2021 May 25;36(2):ME20145. doi: 10.1264/jsme2.ME20145 (PMC8209453; doi:10.1264/jsme2.ME20145)
Supplement: Supplementary file 3 — Supplementary Material 3 [file 36_20145_s3.pdf]

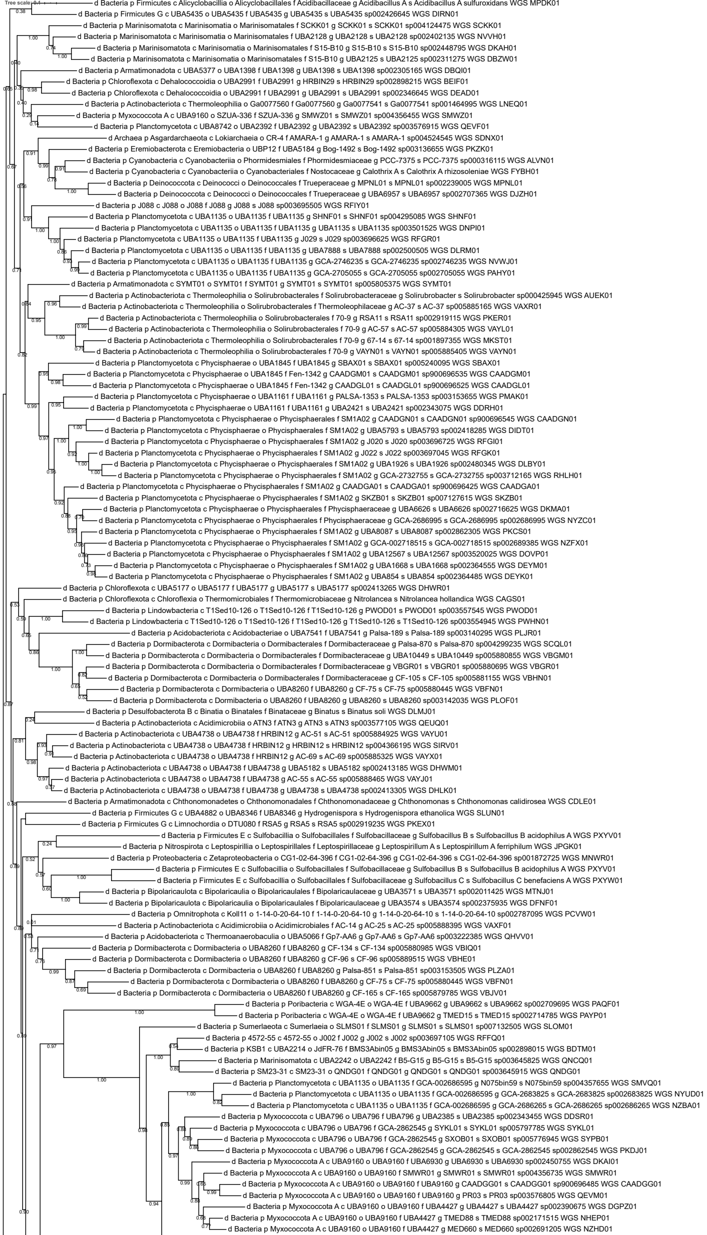
Tree scale: 0.1
d Bacteria p Firmicutes c Alicyclobacillia o Alicyclobacillales f Acidibacillaceae g Acidibacillus A s Acidibacillus A sulfuroxidans WGS MPDK01
d Bacteria p Firmicutes G c UBA5435 o UBA5435 f UBA5435 g UBA5435 s UBA5435 sp002426645 WGS DIRN01
d Bacteria p Marinisomatota c Marinisomatia o Marinisomatales f SCKK01 g SCKK01 s SCKK01 sp004124475 WGS SCKK01
d Bacteria p Marinisomatota c Marinisomatia o Marinisomatales f UBA2128 g UBA2128 s UBA2128 sp002402135 WGS NVVH01
d Bacteria p Marinisomatota c Marinisomatia o Marinisomatales f S15-B10 g S15-B10 s S15-B10 sp002448795 WGS DKAH01
d Bacteria p Marinisomatota c Marinisomatia o Marinisomatales f S15-B10 g UBA2125 s UBA2125 sp002311275 WGS DBZW01
d Bacteria p Armatimonadota c UBA5377 o UBA1398 f UBA1398 g UBA1398 s UBA1398 sp002305165 WGS DBQI01
d Bacteria p Chloroflexota c Dehalococcoidia o UBA2991 f UBA2991 g HRBIN29 s HRBIN29 sp002898215 WGS BEIF01
d Bacteria p Chloroflexota c Dehalococcoidia o UBA2991 f UBA2991 g UBA2991 s UBA2991 sp002346645 WGS DEAD01
d Bacteria p Actinobacteriota c Thermoleophilia o Ga0077560 f Ga0077560 g Ga0077541 s Ga0077541 sp001464995 WGS LNEQ01
d Bacteria p Myxococcota A c UBA9160 o SZUA-336 f SZUA-336 g SMWZ01 s SMWZ01 sp004356455 WGS SMWZ01
d Bacteria p Planctomycetota c UBA8742 o UBA2392 f UBA2392 g UBA2392 s UBA2392 sp003576915 WGS QEVF01
d Archaea p Asgardarchaeota c Lokiarchaeia o CR-4 f AMARA-1 g AMARA-1 s AMARA-1 sp004524545 WGS SDNX01
d Bacteria p Eremiobacterota c Eremiobacteria o UBP12 f UBA5184 g Bog-1492 s Bog-1492 sp003136655 WGS PKZK01
d Bacteria p Cyanobacteria c Cyanobacteriia o Phormidesmiales f Phormidesmiaceae g PCC-7375 s PCC-7375 sp000316115 WGS ALVN01
d Bacteria p Cyanobacteria c Cyanobacteriia o Cyanobacteriales f Nostocaceae g Calothrix A s Calothrix A rhizosoleniae WGS FYBH01
d Bacteria p Deinococcota c Deinococci o Deinococcales f Trueperaceae g MPNL01 s MPNL01 sp002239005 WGS MPNL01
d Bacteria p Deinococcota c Deinococci o Deinococcales f Trueperaceae g UBA6957 s UBA6957 sp002707365 WGS DJZH01
d Bacteria p J088 c J088 o J088 f J088 g J088 s J088 sp003695505 WGS RFIY01
d Bacteria p Planctomycetota c UBA1135 o UBA1135 f UBA1135 g SHNF01 s SHNF01 sp004295085 WGS SHNF01
d Bacteria p Planctomycetota c UBA1135 o UBA1135 f UBA1135 g UBA1135 s UBA1135 sp003501525 WGS DNPI01
d Bacteria p Planctomycetota c UBA1135 o UBA1135 f UBA1135 g J029 s J029 sp003696625 WGS RFGR01
d Bacteria p Planctomycetota c UBA1135 o UBA1135 f UBA1135 g UBA7888 s UBA7888 sp002500505 WGS DLRM01
d Bacteria p Planctomycetota c UBA1135 o UBA1135 f UBA1135 g GCA-2746235 s GCA-2746235 sp002746235 WGS NVWJ01
d Bacteria p Planctomycetota c UBA1135 o UBA1135 f UBA1135 g GCA-2705055 s GCA-2705055 sp002705055 WGS PAHY01
d Bacteria p Armatimonadota c SYMT01 o SYMT01 f SYMT01 g SYMT01 s SYMT01 sp005805375 WGS SYMT01
d Bacteria p Actinobacteriota c Thermoleophilia o Solirubrobacterales f Solirubrobacteraceae g Solirubrobacter s Solirubrobacter sp000425945 WGS AUEK01
d Bacteria p Actinobacteriota c Thermoleophilia o Solirubrobacterales f Thermoleophilaceae g AC-37 s AC-37 sp005885165 WGS VAXR01
d Bacteria p Actinobacteriota c Thermoleophilia o Solirubrobacterales f 70-9 g RSA11 s RSA11 sp002919115 WGS PKER01
d Bacteria p Actinobacteriota c Thermoleophilia o Solirubrobacterales f 70-9 g AC-57 s AC-57 sp005884305 WGS VAYL01
d Bacteria p Actinobacteriota c Thermoleophilia o Solirubrobacterales f 70-9 g 67-14 s 67-14 sp001897355 WGS MKST01
d Bacteria p Actinobacteriota c Thermoleophilia o Solirubrobacterales f 70-9 g VAYN01 s VAYN01 sp005885405 WGS VAYN01
d Bacteria p Planctomycetota c Phycisphaerae o UBA1845 f UBA1845 g SBAX01 s SBAX01 sp005240095 WGS SBAX01
d Bacteria p Planctomycetota c Phycisphaerae o UBA1845 f Fen-1342 g CAADGM01 s CAADGM01 sp900696535 WGS CAADGM01
d Bacteria p Planctomycetota c Phycisphaerae o UBA1845 f Fen-1342 g CAADGL01 s CAADGL01 sp900696525 WGS CAADGL01
d Bacteria p Planctomycetota c Phycisphaerae o UBA1161 f UBA1161 g PALSA-1353 s PALSA-1353 sp003153655 WGS PMAK01
d Bacteria p Planctomycetota c Phycisphaerae o UBA1161 f UBA1161 g UBA2421 s UBA2421 sp002343075 WGS DDRH01
d Bacteria p Planctomycetota c Phycisphaerae o Phycisphaerales f SM1A02 g CAADGN01 s CAADGN01 sp900696545 WGS CAADGN01
d Bacteria p Planctomycetota c Phycisphaerae o Phycisphaerales f SM1A02 g UBA5793 s UBA5793 sp002418285 WGS DIDT01
d Bacteria p Planctomycetota c Phycisphaerae o Phycisphaerales f SM1A02 g J020 s J020 sp003696725 WGS RFGI01
d Bacteria p Planctomycetota c Phycisphaerae o Phycisphaerales f SM1A02 g J022 s J022 sp003697045 WGS RFGK01
d Bacteria p Planctomycetota c Phycisphaerae o Phycisphaerales f SM1A02 g UBA1926 s UBA1926 sp002480345 WGS DLBY01
d Bacteria p Planctomycetota c Phycisphaerae o Phycisphaerales f SM1A02 g GCA-2732755 s GCA-2732755 sp003712165 WGS RHLH01
d Bacteria p Planctomycetota c Phycisphaerae o Phycisphaerales f SM1A02 g CAADGA01 s CAADGA01 sp900696425 WGS CAADGA01
d Bacteria p Planctomycetota c Phycisphaerae o Phycisphaerales f SM1A02 g SKZB01 s SKZB01 sp007127615 WGS SKZB01
d Bacteria p Planctomycetota c Phycisphaerae o Phycisphaerales f Phycisphaeraceae g UBA6626 s UBA6626 sp002716625 WGS DKMA01
d Bacteria p Planctomycetota c Phycisphaerae o Phycisphaerales f Phycisphaeraceae g GCA-2686995 s GCA-2686995 sp002686995 WGS NYZC01
d Bacteria p Planctomycetota c Phycisphaerae o Phycisphaerales f SM1A02 g UBA8087 s UBA8087 sp002862305 WGS PKCS01
d Bacteria p Planctomycetota c Phycisphaerae o Phycisphaerales f SM1A02 g GCA-002718515 s GCA-002718515 sp002689385 WGS NZFX01
d Bacteria p Planctomycetota c Phycisphaerae o Phycisphaerales f SM1A02 g UBA12567 s UBA12567 sp003520025 WGS DOVP01
d Bacteria p Planctomycetota c Phycisphaerae o Phycisphaerales f SM1A02 g UBA1668 s UBA1668 sp002364555 WGS DEYM01
d Bacteria p Planctomycetota c Phycisphaerae o Phycisphaerales f SM1A02 g UBA854 s UBA854 sp002364485 WGS DEYK01
d Bacteria p Chloroflexota c UBA5177 o UBA5177 f UBA5177 g UBA5177 s UBA5177 sp002413265 WGS DHWR01
d Bacteria p Chloroflexota c Chloroflexia o Thermomicrobiales f Thermomicrobiaceae g Nitrolancea s Nitrolancea hollandica WGS CAGS01
d Bacteria p Lindowbacteria c T1Sed10-126 o T1Sed10-126 f T1Sed10-126 g PWOD01 s PWOD01 sp003557545 WGS PWOD01
d Bacteria p Lindowbacteria c T1Sed10-126 o T1Sed10-126 f T1Sed10-126 g T1Sed10-126 s T1Sed10-126 sp003554945 WGS PWHN01
d Bacteria p Acidobacteriota c Acidobacteriae o UBA7541 f UBA7541 g Palsa-189 s Palsa-189 sp003140295 WGS PLJR01
d Bacteria p Dormibacterota c Dormibacteria o Dormibacterales f Dormibacteraceae g Palsa-870 s Palsa-870 sp004299235 WGS SCQL01
d Bacteria p Dormibacterota c Dormibacteria o Dormibacterales f Dormibacteraceae g UBA10449 s UBA10449 sp005880855 WGS VBGM01
d Bacteria p Dormibacterota c Dormibacteria o Dormibacterales f Dormibacteraceae g VBGR01 s VBGR01 sp005880695 WGS VBGR01
d Bacteria p Dormibacterota c Dormibacteria o Dormibacterales f Dormibacteraceae g CF-105 s CF-105 sp005881155 WGS VBHN01
d Bacteria p Dormibacterota c Dormibacteria o UBA8260 f UBA8260 g CF-75 s CF-75 sp005880445 WGS VBFN01
d Bacteria p Dormibacterota c Dormibacteria o UBA8260 f UBA8260 g UBA8260 s UBA8260 sp003142035 WGS PLOF01
d Bacteria p Desulfobacterota B c Binatia o Binatales f Binataceae g Binatus s Binatus soli WGS DLMJ01
d Bacteria p Actinobacteriota c Acidimicrobiia o ATN3 f ATN3 g ATN3 s ATN3 sp003577105 WGS QEUQ01
d Bacteria p Actinobacteriota c UBA4738 o UBA4738 f HRBIN12 g AC-51 s AC-51 sp005884925 WGS VAYU01
d Bacteria p Actinobacteriota c UBA4738 o UBA4738 f HRBIN12 g HRBIN12 s HRBIN12 sp004366195 WGS SIRV01
d Bacteria p Actinobacteriota c UBA4738 o UBA4738 f HRBIN12 g AC-69 s AC-69 sp005885325 WGS VAYX01
d Bacteria p Actinobacteriota c UBA4738 o UBA4738 f UBA4738 g UBA5182 s UBA5182 sp002413185 WGS DHWM01
d Bacteria p Actinobacteriota c UBA4738 o UBA4738 f UBA4738 g AC-55 s AC-55 sp005888465 WGS VAYJ01
d Bacteria p Actinobacteriota c UBA4738 o UBA4738 f UBA4738 g UBA4738 s UBA4738 sp002413305 WGS DHLK01
d Bacteria p Armatimonadota c Chthonomonadetes o Chthonomonadales f Chthonomonadaceae g Chthonomonas s Chthonomonas calidirosea WGS CDLE01
d Bacteria p Firmicutes G c UBA4882 o UBA8346 f UBA8346 g Hydrogenispora s Hydrogenispora ethanolica WGS SLUN01
d Bacteria p Firmicutes G c Limnochordia o DTU080 f RSA5 g RSA5 s RSA5 sp002919235 WGS PKEX01
d Bacteria p Firmicutes E c Sulfobacillia o Sulfobacillales f Sulfobacillaceae g Sulfobacillus B s Sulfobacillus B acidophilus A WGS PXYV01
d Bacteria p Nitrospirota c Leptospirillia o Leptospirillales f Leptospirillaceae g Leptospirillum A s Leptospirillum A ferriphilum WGS JPGK01
d Bacteria p Proteobacteria c Zetaproteobacteria o CG1-02-64-396 f CG1-02-64-396 g CG1-02-64-396 s CG1-02-64-396 sp001872725 WGS MNWR01
d Bacteria p Firmicutes E c Sulfobacillia o Sulfobacillales f Sulfobacillaceae g Sulfobacillus B s Sulfobacillus B acidophilus A WGS PXYV01
d Bacteria p Firmicutes E c Sulfobacillia o Sulfobacillales f Sulfobacillaceae g Sulfobacillus C s Sulfobacillus C benefaciens A WGS PXYW01
d Bacteria p Bipolaricaulota c Bipolaricaulia o Bipolaricaulales f Bipolaricaulaceae g UBA3571 s UBA3571 sp002011425 WGS MTNJ01
d Bacteria p Bipolaricaulota c Bipolaricaulia o Bipolaricaulales f Bipolaricaulaceae g UBA3574 s UBA3574 sp002375935 WGS DFNF01
d Bacteria p Omnitrophota c Koll11 o 1-14-0-20-64-10 f 1-14-0-20-64-10 g 1-14-0-20-64-10 s 1-14-0-20-64-10 sp002787095 WGS PCVW01
d Bacteria p Actinobacteriota c Acidimicrobiia o Acidimicrobiales f AC-14 g AC-25 s AC-25 sp005888395 WGS VAXF01
d Bacteria p Acidobacteriota c Thermoanaerobaculia o UBA5066 f Gp7-AA6 g Gp7-AA6 s Gp7-AA6 sp003222385 WGS QHVV01
d Bacteria p Dormibacterota c Dormibacteria o UBA8260 f UBA8260 g CF-134 s CF-134 sp005880985 WGS VBIQ01
d Bacteria p Dormibacterota c Dormibacteria o UBA8260 f UBA8260 g CF-96 s CF-96 sp005889515 WGS VBHE01
d Bacteria p Dormibacterota c Dormibacteria o UBA8260 f UBA8260 g Palsa-851 s Palsa-851 sp003153505 WGS PLZA01
d Bacteria p Dormibacterota c Dormibacteria o UBA8260 f UBA8260 g CF-75 s CF-75 sp005880445 WGS VBFN01
d Bacteria p Dormibacterota c Dormibacteria o UBA8260 f UBA8260 g CF-165 s CF-165 sp005879785 WGS VBJV01
d Bacteria p Poribacteria c WGA-4E o WGA-4E f UBA9662 g UBA9662 s UBA9662 sp002709695 WGS PAQF01
d Bacteria p Poribacteria c WGA-4E o WGA-4E f UBA9662 g TMED15 s TMED15 sp002714785 WGS PAYP01
d Bacteria p Sumerlaeota c Sumerlaeia o SLMS01 f SLMS01 g SLMS01 s SLMS01 sp007132505 WGS SLOM01
d Bacteria p 4572-55 c 4572-55 o J002 f J002 g J002 s J002 sp003697105 WGS RFFQ01
d Bacteria p KSB1 c UBA2214 o JdFR-76 f BMS3Abin05 g BMS3Abin05 s BMS3Abin05 sp002898015 WGS BDTM01
d Bacteria p Marinisomatota c UBA2242 o UBA2242 f B5-G15 g B5-G15 s B5-G15 sp003645825 WGS QNCQ01
d Bacteria p SM23-31 c SM23-31 o QNDG01 f QNDG01 g QNDG01 s QNDG01 sp003645915 WGS QNDG01
d Bacteria p Planctomycetota c UBA1135 o UBA1135 f GCA-002686595 g N075bin59 s N075bin59 sp004357655 WGS SMVQ01
d Bacteria p Planctomycetota c UBA1135 o UBA1135 f GCA-002686595 g GCA-2683825 s GCA-2683825 sp002683825 WGS NYUD01
d Bacteria p Planctomycetota c UBA1135 o UBA1135 f GCA-002686595 g GCA-2686265 s GCA-2686265 sp002686265 WGS NZBA01
d Bacteria p Myxococcota c UBA796 o UBA796 f UBA796 g UBA2385 s UBA2385 sp002343455 WGS DDSR01
d Bacteria p Myxococcota c UBA796 o UBA796 f GCA-2862545 g SYKL01 s SYKL01 sp005797785 WGS SYKL01
d Bacteria p Myxococcota c UBA796 o UBA796 f GCA-2862545 g SXOB01 s SXOB01 sp005776945 WGS SYPB01
d Bacteria p Myxococcota c UBA796 o UBA796 f GCA-2862545 g GCA-2862545 s GCA-2862545 sp002862545 WGS PKDJ01
d Bacteria p Myxococcota A c UBA9160 o UBA9160 f UBA6930 g UBA6930 s UBA6930 sp002450755 WGS DKAI01
d Bacteria p Myxococcota A c UBA9160 o UBA9160 f SMWR01 g SMWR01 s SMWR01 sp004356735 WGS SMWR01
d Bacteria p Myxococcota A c UBA9160 o UBA9160 f UBA9160 g CAADGG01 s CAADGG01 sp900696485 WGS CAADGG01
d Bacteria p Myxococcota A c UBA9160 o UBA9160 f UBA9160 g PR03 s PR03 sp003576805 WGS QEVM01
d Bacteria p Myxococcota A c UBA9160 o UBA9160 f UBA4427 g UBA4427 s UBA4427 sp002390675 WGS DGPZ01
d Bacteria p Myxococcota A c UBA9160 o UBA9160 f UBA4427 g TMED88 s TMED88 sp002171515 WGS NHEP01
d Bacteria p Myxococcota A c UBA9160 o UBA9160 f UBA4427 g MED660 s MED660 sp002691205 WGS NZHD01

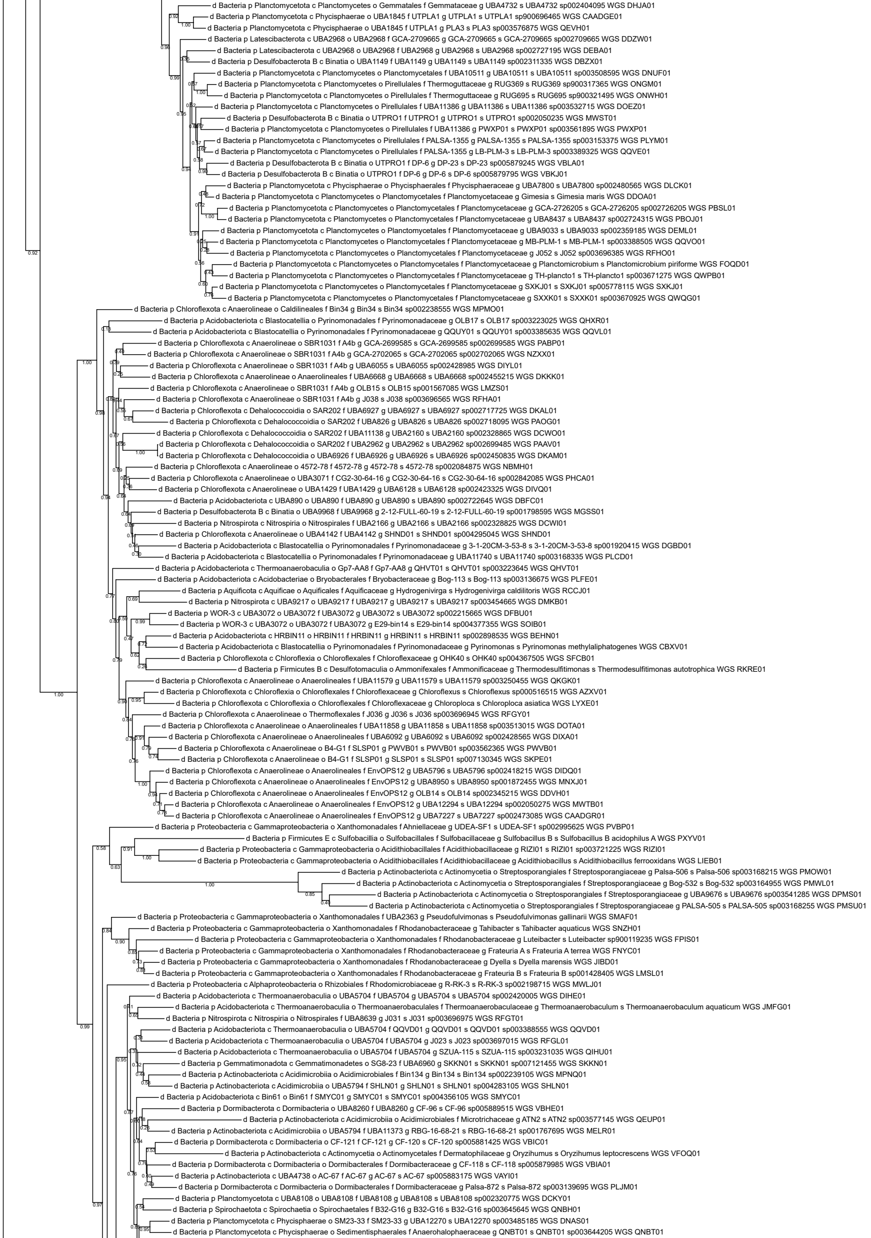

d Bacteria p Planctomycetota c Planctomycetes o Gemmatales f Gemmataceae g UBA4732 s UBA4732 sp002404095 WGS DHJA01
d Bacteria p Planctomycetota c Phycisphaerae o UBA1845 f UTPLA1 g UTPLA1 s UTPLA1 sp900696465 WGS CAADGE01
d Bacteria p Planctomycetota c Phycisphaerae o UBA1845 f UTPLA1 g PLA3 s PLA3 sp003576875 WGS QEVH01
d Bacteria p Latescibacterota c UBA2968 o UBA2968 f GCA-2709665 g GCA-2709665 s GCA-2709665 sp002709665 WGS DDZW01
d Bacteria p Latescibacterota c UBA2968 o UBA2968 f UBA2968 g UBA2968 s UBA2968 sp002727195 WGS DEBA01
d Bacteria p Desulfobacterota B c Binatia o UBA1149 f UBA1149 g UBA1149 s UBA1149 sp002311335 WGS DBZX01
d Bacteria p Planctomycetota c Planctomycetes o Planctomycetales f UBA10511 g UBA10511 s UBA10511 sp003508595 WGS DNUF01
d Bacteria p Planctomycetota c Planctomycetes o Pirellulales f Thermoguttaceae g RUG369 s RUG369 sp900317365 WGS ONGM01
d Bacteria p Planctomycetota c Planctomycetes o Pirellulales f Thermoguttaceae g RUG695 s RUG695 sp900321495 WGS ONWH01
d Bacteria p Planctomycetota c Planctomycetes o Pirellulales f UBA11386 g UBA11386 s UBA11386 sp003532715 WGS DOEZ01
d Bacteria p Desulfobacterota B c Binatia o UTPRO1 f UTPRO1 g UTPRO1 s UTPRO1 sp002050235 WGS MWST01
d Bacteria p Planctomycetota c Planctomycetes o Pirellulales f UBA11386 g PWXP01 s PWXP01 sp003561895 WGS PWXP01
d Bacteria p Planctomycetota c Planctomycetes o Pirellulales f PALSA-1355 g PALSA-1355 s PALSA-1355 sp003153375 WGS PLYM01
d Bacteria p Planctomycetota c Planctomycetes o Pirellulales f PALSA-1355 g LB-PLM-3 s LB-PLM-3 sp003389325 WGS QQVE01
d Bacteria p Desulfobacterota B c Binatia o UTPRO1 f DP-6 g DP-23 s DP-23 sp005879245 WGS VBLA01
d Bacteria p Desulfobacterota B c Binatia o UTPRO1 f DP-6 g DP-6 s DP-6 sp005879795 WGS VBKJ01
d Bacteria p Planctomycetota c Phycisphaerae o Phycisphaerales f Phycisphaeraceae g UBA7800 s UBA7800 sp002480565 WGS DLCK01
d Bacteria p Planctomycetota c Planctomycetes o Planctomycetales f Planctomycetaceae g Gimesia s Gimesia maris WGS DDOA01
d Bacteria p Planctomycetota c Planctomycetes o Planctomycetales f Planctomycetaceae g GCA-2726205 s GCA-2726205 sp002726205 WGS PBSL01
d Bacteria p Planctomycetota c Planctomycetes o Planctomycetales f Planctomycetaceae g UBA8437 s UBA8437 sp002724315 WGS PBOJ01
d Bacteria p Planctomycetota c Planctomycetes o Planctomycetales f Planctomycetaceae g UBA9033 s UBA9033 sp002359185 WGS DEML01
d Bacteria p Planctomycetota c Planctomycetes o Planctomycetales f Planctomycetaceae g MB-PLM-1 s MB-PLM-1 sp003388505 WGS QQVO01
d Bacteria p Planctomycetota c Planctomycetes o Planctomycetales f Planctomycetaceae g J052 s J052 sp003696385 WGS RFHO01
d Bacteria p Planctomycetota c Planctomycetes o Planctomycetales f Planctomycetaceae g Planctomicrobium s Planctomicrobium piriforme WGS FOQD01
d Bacteria p Planctomycetota c Planctomycetes o Planctomycetales f Planctomycetaceae g TH-plancto1 s TH-plancto1 sp003671275 WGS QWPB01
d Bacteria p Planctomycetota c Planctomycetes o Planctomycetales f Planctomycetaceae g SXKJ01 s SXKJ01 sp005778115 WGS SXKJ01
d Bacteria p Planctomycetota c Planctomycetes o Planctomycetales f Planctomycetaceae g SXXK01 s SXXK01 sp003670925 WGS QWQG01
d Bacteria p Chloroflexota c Anaerolineae o Caldilineales f Bin34 g Bin34 s Bin34 sp002238555 WGS MPMO01
d Bacteria p Acidobacteriota c Blastocatellia o Pyrinomonadales f Pyrinomonadaceae g OLB17 s OLB17 sp003223025 WGS QHXR01
d Bacteria p Acidobacteriota c Blastocatellia o Pyrinomonadales f Pyrinomonadaceae g QQUY01 s QQUY01 sp003385635 WGS QQVL01
d Bacteria p Chloroflexota c Anaerolineae o SBR1031 f A4b g GCA-2699585 s GCA-2699585 sp002699585 WGS PABP01
d Bacteria p Chloroflexota c Anaerolineae o SBR1031 f A4b g GCA-2702065 s GCA-2702065 sp002702065 WGS NZXX01
d Bacteria p Chloroflexota c Anaerolineae o SBR1031 f A4b g UBA6055 s UBA6055 sp002428985 WGS DIYL01
d Bacteria p Chloroflexota c Anaerolineae o Anaerolineales f UBA6668 g UBA6668 s UBA6668 sp002455215 WGS DKKK01
d Bacteria p Chloroflexota c Anaerolineae o SBR1031 f A4b g OLB15 s OLB15 sp001567085 WGS LMZS01
d Bacteria p Chloroflexota c Anaerolineae o SBR1031 f A4b g J038 s J038 sp003696565 WGS RFHA01
d Bacteria p Chloroflexota c Dehalococcoidia o SAR202 f UBA6927 g UBA6927 s UBA6927 sp002717725 WGS DKAL01
d Bacteria p Chloroflexota c Dehalococcoidia o SAR202 f UBA826 g UBA826 s UBA826 sp002718095 WGS PAOG01
d Bacteria p Chloroflexota c Dehalococcoidia o SAR202 f UBA11138 g UBA2160 s UBA2160 sp002328865 WGS DCWO01
d Bacteria p Chloroflexota c Dehalococcoidia o SAR202 f UBA2962 g UBA2962 s UBA2962 sp002699485 WGS PAAV01
d Bacteria p Chloroflexota c Dehalococcoidia o UBA6926 f UBA6926 g UBA6926 s UBA6926 sp002450835 WGS DKAM01
d Bacteria p Chloroflexota c Anaerolineae o 4572-78 f 4572-78 g 4572-78 s 4572-78 sp002084875 WGS NBMH01
d Bacteria p Chloroflexota c Anaerolineae o UBA3071 f CG2-30-64-16 g CG2-30-64-16 s CG2-30-64-16 sp002842085 WGS PHCA01
d Bacteria p Chloroflexota c Anaerolineae o UBA1429 f UBA1429 g UBA6128 s UBA6128 sp002423325 WGS DIVQ01
d Bacteria p Acidobacteriota c UBA890 o UBA890 f UBA890 g UBA890 s UBA890 sp002722645 WGS DBFC01
d Bacteria p Desulfobacterota B c Binatia o UBA9968 f UBA9968 g 2-12-FULL-60-19 s 2-12-FULL-60-19 sp001798595 WGS MGSS01
d Bacteria p Nitrospirota c Nitrospiria o Nitrospirales f UBA2166 g UBA2166 s UBA2166 sp002328825 WGS DCWI01
d Bacteria p Chloroflexota c Anaerolineae o UBA4142 f UBA4142 g SHND01 s SHND01 sp004295045 WGS SHND01
d Bacteria p Acidobacteriota c Blastocatellia o Pyrinomonadales f Pyrinomonadaceae g 3-1-20CM-3-53-8 s 3-1-20CM-3-53-8 sp001920415 WGS DGBD01
d Bacteria p Acidobacteriota c Blastocatellia o Pyrinomonadales f Pyrinomonadaceae g UBA11740 s UBA11740 sp003168335 WGS PLCD01
d Bacteria p Acidobacteriota c Thermoanaerobaculia o Gp7-AA8 f Gp7-AA8 g QHVT01 s QHVT01 sp003223645 WGS QHVT01
d Bacteria p Acidobacteriota c Acidobacteriae o Bryobacterales f Bryobacteraceae g Bog-113 s Bog-113 sp003136675 WGS PLFE01
d Bacteria p Aquificota c Aquificae o Aquificales f Aquificaceae g Hydrogenivirga s Hydrogenivirga caldilitoris WGS RCCJ01
d Bacteria p Nitrospirota c UBA9217 o UBA9217 f UBA9217 g UBA9217 s UBA9217 sp003454665 WGS DMKB01
d Bacteria p WOR-3 c UBA3072 o UBA3072 f UBA3072 g UBA3072 s UBA3072 sp002215665 WGS DFBU01
d Bacteria p WOR-3 c UBA3072 o UBA3072 f UBA3072 g E29-bin14 s E29-bin14 sp004377355 WGS SOIB01
d Bacteria p Acidobacteriota c HRBIN11 o HRBIN11 f HRBIN11 g HRBIN11 s HRBIN11 sp002898535 WGS BEHN01
d Bacteria p Acidobacteriota c Blastocatellia o Pyrinomonadales f Pyrinomonadaceae g Pyrinomonas s Pyrinomonas methylaliphatogenes WGS CBXV01
d Bacteria p Chloroflexota c Chloroflexia o Chloroflexales f Chloroflexaceae g OHK40 s OHK40 sp004367505 WGS SFCB01
d Bacteria p Firmicutes B c Desulfotomaculia o Ammonifexales f Ammonificaceae g Thermodesulfitimonas s Thermodesulfitimonas autotrophica WGS RKRE01
d Bacteria p Chloroflexota c Anaerolineae o Anaerolineales f UBA11579 g UBA11579 s UBA11579 sp003250455 WGS QKGK01
d Bacteria p Chloroflexota c Chloroflexia o Chloroflexales f Chloroflexaceae g Chloroflexus s Chloroflexus sp000516515 WGS AZXV01
d Bacteria p Chloroflexota c Chloroflexia o Chloroflexales f Chloroflexaceae g Chloroploca s Chloroploca asiatica WGS LYXE01
d Bacteria p Chloroflexota c Anaerolineae o Thermoflexales f J036 g J036 s J036 sp003696945 WGS RFGY01
d Bacteria p Chloroflexota c Anaerolineae o Anaerolineales f UBA11858 g UBA11858 s UBA11858 sp003513015 WGS DOTA01
d Bacteria p Chloroflexota c Anaerolineae o Anaerolineales f UBA6092 g UBA6092 s UBA6092 sp002428565 WGS DIXA01
d Bacteria p Chloroflexota c Anaerolineae o B4-G1 f SLSP01 g PWVB01 s PWVB01 sp003562365 WGS PWVB01
d Bacteria p Chloroflexota c Anaerolineae o B4-G1 f SLSP01 g SLSP01 s SLSP01 sp007130345 WGS SKPE01
d Bacteria p Chloroflexota c Anaerolineae o Anaerolineales f EnvOPS12 g UBA5796 s UBA5796 sp002418215 WGS DIDQ01
d Bacteria p Chloroflexota c Anaerolineae o Anaerolineales f EnvOPS12 g UBA8950 s UBA8950 sp001872455 WGS MNXJ01
d Bacteria p Chloroflexota c Anaerolineae o Anaerolineales f EnvOPS12 g OLB14 s OLB14 sp002345215 WGS DDVH01
d Bacteria p Chloroflexota c Anaerolineae o Anaerolineales f EnvOPS12 g UBA12294 s UBA12294 sp002050275 WGS MWTB01
d Bacteria p Chloroflexota c Anaerolineae o Anaerolineales f EnvOPS12 g UBA7227 s UBA7227 sp002473085 WGS CAADGR01
d Bacteria p Proteobacteria c Gammaproteobacteria o Xanthomonadales f Ahniellaceae g UDEA-SF1 s UDEA-SF1 sp002995625 WGS PVBP01
d Bacteria p Firmicutes E c Sulfobacillia o Sulfobacillales f Sulfobacillaceae g Sulfobacillus B s Sulfobacillus B acidophilus A WGS PXYV01
d Bacteria p Proteobacteria c Gammaproteobacteria o Acidithiobacillales f Acidithiobacillaceae g RIZI01 s RIZI01 sp003721225 WGS RIZI01
d Bacteria p Proteobacteria c Gammaproteobacteria o Acidithiobacillales f Acidithiobacillaceae g Acidithiobacillus s Acidithiobacillus ferrooxidans WGS LIEB01
d Bacteria p Actinobacteriota c Actinomycetia o Streptosporangiales f Streptosporangiaceae g Palsa-506 s Palsa-506 sp003168215 WGS PMOW01
d Bacteria p Actinobacteriota c Actinomycetia o Streptosporangiales f Streptosporangiaceae g Bog-532 s Bog-532 sp003164955 WGS PMWL01
d Bacteria p Actinobacteriota c Actinomycetia o Streptosporangiales f Streptosporangiaceae g UBA9676 s UBA9676 sp003541285 WGS DPMS01
d Bacteria p Actinobacteriota c Actinomycetia o Streptosporangiales f Streptosporangiaceae g PALSA-505 s PALSA-505 sp003168255 WGS PMSU01
d Bacteria p Proteobacteria c Gammaproteobacteria o Xanthomonadales f UBA2363 g Pseudofulvimonas s Pseudofulvimonas gallinarii WGS SMAF01
d Bacteria p Proteobacteria c Gammaproteobacteria o Xanthomonadales f Rhodanobacteraceae g Tahibacter s Tahibacter aquaticus WGS SNZH01
d Bacteria p Proteobacteria c Gammaproteobacteria o Xanthomonadales f Rhodanobacteraceae g Luteibacter s Luteibacter sp900119235 WGS FPIS01
d Bacteria p Proteobacteria c Gammaproteobacteria o Xanthomonadales f Rhodanobacteraceae g Frateuria A s Frateuria A terrea WGS FNYC01
d Bacteria p Proteobacteria c Gammaproteobacteria o Xanthomonadales f Rhodanobacteraceae g Dyella s Dyella marensis WGS JIBD01
d Bacteria p Proteobacteria c Gammaproteobacteria o Xanthomonadales f Rhodanobacteraceae g Frateuria B s Frateuria B sp001428405 WGS LMSL01
d Bacteria p Proteobacteria c Alphaproteobacteria o Rhizobiales f Rhodomicrobiaceae g R-RK-3 s R-RK-3 sp002198715 WGS MWLJ01
d Bacteria p Acidobacteriota c Thermoanaerobaculia o UBA5704 f UBA5704 g UBA5704 s UBA5704 sp002420005 WGS DIHE01
d Bacteria p Acidobacteriota c Thermoanaerobaculia o Thermoanaerobaculales f Thermoanaerobaculaceae g Thermoanaerobaculum s Thermoanaerobaculum aquaticum WGS JMFG01
d Bacteria p Nitrospirota c Nitrospiria o Nitrospirales f UBA8639 g J031 s J031 sp003696975 WGS RFGT01
d Bacteria p Acidobacteriota c Thermoanaerobaculia o UBA5704 f QQVD01 g QQVD01 s QQVD01 sp003388555 WGS QQVD01
d Bacteria p Acidobacteriota c Thermoanaerobaculia o UBA5704 f UBA5704 g J023 s J023 sp003697015 WGS RFGL01
d Bacteria p Acidobacteriota c Thermoanaerobaculia o UBA5704 f UBA5704 g SZUA-115 s SZUA-115 sp003231035 WGS QIHU01
d Bacteria p Gemmatimonadota c Gemmatimonadetes o SG8-23 f UBA6960 g SKKN01 s SKKN01 sp007121455 WGS SKKN01
d Bacteria p Actinobacteriota c Acidimicrobiia o Acidimicrobiales f Bin134 g Bin134 s Bin134 sp002239105 WGS MPNQ01
d Bacteria p Actinobacteriota c Acidimicrobiia o UBA5794 f SHLN01 g SHLN01 s SHLN01 sp004283105 WGS SHLN01
d Bacteria p Acidobacteriota c Bin61 o Bin61 f SMYC01 g SMYC01 s SMYC01 sp004356105 WGS SMYC01
d Bacteria p Dormibacterota c Dormibacteria o UBA8260 f UBA8260 g CF-96 s CF-96 sp005889515 WGS VBHE01
d Bacteria p Actinobacteriota c Acidimicrobiia o Acidimicrobiales f Microtrichaceae g ATN2 s ATN2 sp003577145 WGS QEUP01
d Bacteria p Actinobacteriota c Acidimicrobiia o UBA5794 f UBA11373 g RBG-16-68-21 s RBG-16-68-21 sp001767695 WGS MELR01
d Bacteria p Dormibacterota c Dormibacteria o CF-121 f CF-121 g CF-120 s CF-120 sp005881425 WGS VBIC01
d Bacteria p Actinobacteriota c Actinomycetia o Actinomycetales f Dermatophilaceae g Oryzihumus s Oryzihumus leptocrescens WGS VFOQ01
d Bacteria p Dormibacterota c Dormibacteria o Dormibacterales f Dormibacteraceae g CF-118 s CF-118 sp005879985 WGS VBIA01
d Bacteria p Actinobacteriota c UBA4738 o AC-67 f AC-67 g AC-67 s AC-67 sp005883175 WGS VAYI01
d Bacteria p Dormibacterota c Dormibacteria o Dormibacterales f Dormibacteraceae g Palsa-872 s Palsa-872 sp003139695 WGS PLJM01
d Bacteria p Planctomycetota c UBA8108 o UBA8108 f UBA8108 g UBA8108 s UBA8108 sp002320775 WGS DCKY01
d Bacteria p Spirochaetota c Spirochaetia o Spirochaetales f B32-G16 g B32-G16 s B32-G16 sp003645645 WGS QNBH01
d Bacteria p Planctomycetota c Phycisphaerae o SM23-33 f SM23-33 g UBA12270 s UBA12270 sp003485185 WGS DNAS01
d Bacteria p Planctomycetota c Phycisphaerae o Sedimentisphaerales f Anaerohalophaeraceae g QNBT01 s QNBT01 sp003644205 WGS QNBT01

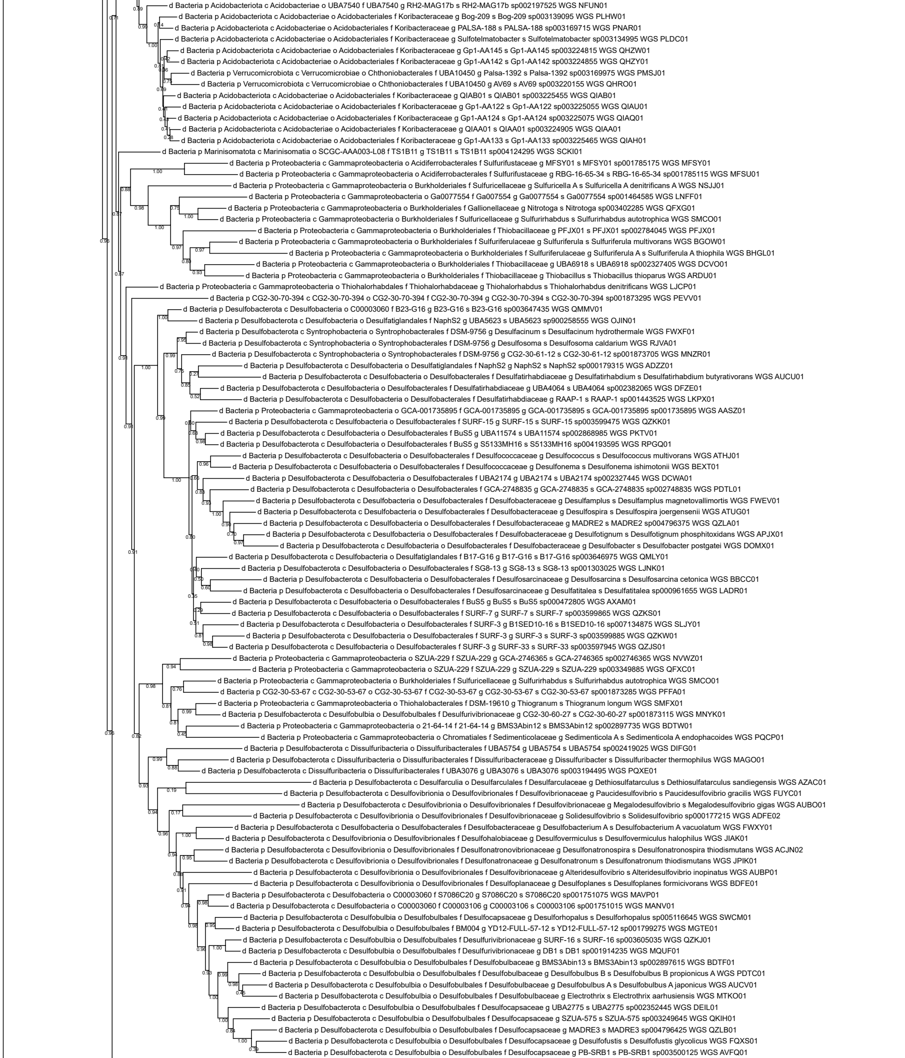
d Bacteria p Acidobacteriota c Acidobacteriae o UBA7540 f UBA7540 g RH2-MAG17b s RH2-MAG17b sp002197525 WGS NFUN01
d Bacteria p Acidobacteriota c Acidobacteriae o Acidobacteriales f Koribacteraceae g Bog-209 s Bog-209 sp003139095 WGS PLHW01
d Bacteria p Acidobacteriota c Acidobacteriae o Acidobacteriales f Koribacteraceae g PALSA-188 s PALSA-188 sp003169715 WGS PNAR01
d Bacteria p Acidobacteriota c Acidobacteriae o Acidobacteriales f Koribacteraceae g Sulfotelmatobacter s Sulfotelmatobacter sp003134995 WGS PLDC01
d Bacteria p Acidobacteriota c Acidobacteriae o Acidobacteriales f Koribacteraceae g Gp1-AA145 s Gp1-AA145 sp003224815 WGS QHZW01
d Bacteria p Acidobacteriota c Acidobacteriae o Acidobacteriales f Koribacteraceae g Gp1-AA142 s Gp1-AA142 sp003224855 WGS QHZY01
d Bacteria p Verrucomicrobiota c Verrucomicrobiae o Chthoniobacterales f UBA10450 g Palsa-1392 s Palsa-1392 sp003169975 WGS PMSJ01
d Bacteria p Verrucomicrobiota c Verrucomicrobiae o Chthoniobacterales f UBA10450 g AV69 s AV69 sp003220155 WGS QHRO01
d Bacteria p Acidobacteriota c Acidobacteriae o Acidobacteriales f Koribacteraceae g QIAB01 s QIAB01 sp003225455 WGS QIAB01
d Bacteria p Acidobacteriota c Acidobacteriae o Acidobacteriales f Koribacteraceae g Gp1-AA122 s Gp1-AA122 sp003225055 WGS QIAU01
d Bacteria p Acidobacteriota c Acidobacteriae o Acidobacteriales f Koribacteraceae g Gp1-AA124 s Gp1-AA124 sp003225075 WGS QIAQ01
d Bacteria p Acidobacteriota c Acidobacteriae o Acidobacteriales f Koribacteraceae g QIAA01 s QIAA01 sp003224905 WGS QIAA01
d Bacteria p Acidobacteriota c Acidobacteriae o Acidobacteriales f Koribacteraceae g Gp1-AA133 s Gp1-AA133 sp003225465 WGS QIAH01
d Bacteria p Marinisomatota c Marinisomatia o SCGC-AAA003-L08 f TS1B11 g TS1B11 s TS1B11 sp004124295 WGS SCKI01
d Bacteria p Proteobacteria c Gammaproteobacteria o Acidiferrobacterales f Sulfurifustaceae g MFSY01 s MFSY01 sp001785175 WGS MFSY01
d Bacteria p Proteobacteria c Gammaproteobacteria o Acidiferrobacterales f Sulfurifustaceae g RBG-16-65-34 s RBG-16-65-34 sp001785115 WGS MFSU01
d Bacteria p Proteobacteria c Gammaproteobacteria o Burkholderiales f Sulfuricellaceae g Sulfuricella A s Sulfuricella A denitrificans A WGS NSJJ01
d Bacteria p Proteobacteria c Gammaproteobacteria o Ga0077554 f Ga007554 g Ga0077554 s Ga0077554 sp001464585 WGS LNFF01
d Bacteria p Proteobacteria c Gammaproteobacteria o Burkholderiales f Gallionellaceae g Nitrotoga s Nitrotoga sp003402285 WGS QFXG01
d Bacteria p Proteobacteria c Gammaproteobacteria o Burkholderiales f Sulfuricellaceae g Sulfurirhabdus s Sulfurirhabdus autotrophica WGS SMCO01
d Bacteria p Proteobacteria c Gammaproteobacteria o Burkholderiales f Thiobacillaceae g PFJX01 s PFJX01 sp002784045 WGS PFJX01
d Bacteria p Proteobacteria c Gammaproteobacteria o Burkholderiales f Sulfuriferulaceae g Sulfuriferula s Sulfuriferula multivorans WGS BGOW01
d Bacteria p Proteobacteria c Gammaproteobacteria o Burkholderiales f Sulfuriferulaceae g Sulfuriferula A s Sulfuriferula A thiophila WGS BHGL01
d Bacteria p Proteobacteria c Gammaproteobacteria o Burkholderiales f Thiobacillaceae g UBA6918 s UBA6918 sp002327405 WGS DCVO01
d Bacteria p Proteobacteria c Gammaproteobacteria o Burkholderiales f Thiobacillaceae g Thiobacillus s Thiobacillus thioparus WGS ARDU01
d Bacteria p Proteobacteria c Gammaproteobacteria o Thiohalorhabdales f Thiohalorhabdaceae g Thiohalorhabdus s Thiohalorhabdus denitrificans WGS LJCP01
d Bacteria p CG2-30-70-394 c CG2-30-70-394 o CG2-30-70-394 f CG2-30-70-394 g CG2-30-70-394 s CG2-30-70-394 sp001873295 WGS PEVV01
d Bacteria p Desulfobacterota c Desulfobacteria o C00003060 f B23-G16 g B23-G16 s B23-G16 sp003647435 WGS QMMV01
d Bacteria p Desulfobacterota c Desulfobacteria o Desulfatiglandales f NaphS2 g UBA5623 s UBA5623 sp900258555 WGS OJIN01
d Bacteria p Desulfobacterota c Syntrophobacteria o Syntrophobacterales f DSM-9756 g Desulfacinum s Desulfacinum hydrothermale WGS FWXF01
d Bacteria p Desulfobacterota c Syntrophobacteria o Syntrophobacterales f DSM-9756 g Desulfosoma s Desulfosoma caldarium WGS RJVA01
d Bacteria p Desulfobacterota c Syntrophobacteria o Syntrophobacterales f DSM-9756 g CG2-30-61-12 s CG2-30-61-12 sp001873705 WGS MNZR01
d Bacteria p Desulfobacterota c Desulfobacteria o Desulfatiglandales f NaphS2 g NaphS2 s NaphS2 sp000179315 WGS ADZZ01
d Bacteria p Desulfobacterota c Desulfobacteria o Desulfobacterales f Desulfatirhabdiaceae g Desulfatirhabdium s Desulfatirhabdium butyrativorans WGS AUCU01
d Bacteria p Desulfobacterota c Desulfobacteria o Desulfobacterales f Desulfatirhabdiaceae g UBA4064 s UBA4064 sp002382065 WGS DFZE01
d Bacteria p Desulfobacterota c Desulfobacteria o Desulfobacterales f Desulfatirhabdiaceae g RAAP-1 s RAAP-1 sp001443525 WGS LKPX01
d Bacteria p Proteobacteria c Gammaproteobacteria o GCA-001735895 f GCA-001735895 g GCA-001735895 s GCA-001735895 sp001735895 WGS AASZ01
d Bacteria p Desulfobacterota c Desulfobacteria o Desulfobacterales f SURF-15 g SURF-15 s SURF-15 sp003599475 WGS QZKK01
d Bacteria p Desulfobacterota c Desulfobacteria o Desulfobacterales f BuS5 g UBA11574 s UBA11574 sp002868985 WGS PKTV01
d Bacteria p Desulfobacterota c Desulfobacteria o Desulfobacterales f BuS5 g S5133MH16 s S5133MH16 sp004193595 WGS RPGQ01
d Bacteria p Desulfobacterota c Desulfobacteria o Desulfobacterales f Desulfococcaceae g Desulfococcus s Desulfococcus multivorans WGS ATHJ01
d Bacteria p Desulfobacterota c Desulfobacteria o Desulfobacterales f Desulfococcaceae g Desulfonema s Desulfonema ishimotonii WGS BEXT01
d Bacteria p Desulfobacterota c Desulfobacteria o Desulfobacterales f UBA2174 g UBA2174 s UBA2174 sp002327445 WGS DCWA01
d Bacteria p Desulfobacterota c Desulfobacteria o Desulfobacterales f GCA-2748835 g GCA-2748835 s GCA-2748835 sp002748835 WGS PDTL01
d Bacteria p Desulfobacterota c Desulfobacteria o Desulfobacterales f Desulfobacteraceae g Desulfamplus s Desulfamplus magnetovallimortis WGS FWEV01
d Bacteria p Desulfobacterota c Desulfobacteria o Desulfobacterales f Desulfobacteraceae g Desulfospira s Desulfospira joergensenii WGS ATUG01
d Bacteria p Desulfobacterota c Desulfobacteria o Desulfobacterales f Desulfobacteraceae g MADRE2 s MADRE2 sp004796375 WGS QZLA01
d Bacteria p Desulfobacterota c Desulfobacteria o Desulfobacterales f Desulfobacteraceae g Desulfotignum s Desulfotignum phosphitoxidans WGS APJX01
d Bacteria p Desulfobacterota c Desulfobacteria o Desulfobacterales f Desulfobacteraceae g Desulfobacter s Desulfobacter postgatei WGS DOMX01
d Bacteria p Desulfobacterota c Desulfobacteria o Desulfatiglandales f B17-G16 g B17-G16 s B17-G16 sp003646975 WGS QMLY01
d Bacteria p Desulfobacterota c Desulfobacteria o Desulfobacterales f SG8-13 g SG8-13 s SG8-13 sp001303025 WGS LJNK01
d Bacteria p Desulfobacterota c Desulfobacteria o Desulfobacterales f Desulfosarcinaceae g Desulfosarcina s Desulfosarcina cetonica WGS BBCC01
d Bacteria p Desulfobacterota c Desulfobacteria o Desulfobacterales f Desulfosarcinaceae g Desulfatitalea s Desulfatitalea sp000961655 WGS LADR01
d Bacteria p Desulfobacterota c Desulfobacteria o Desulfobacterales f BuS5 g BuS5 s BuS5 sp000472805 WGS AXAM01
d Bacteria p Desulfobacterota c Desulfobacteria o Desulfobacterales f SURF-7 g SURF-7 s SURF-7 sp003599865 WGS QZKS01
d Bacteria p Desulfobacterota c Desulfobacteria o Desulfobacterales f SURF-3 g B1SED10-16 s B1SED10-16 sp007134875 WGS SLJY01
d Bacteria p Desulfobacterota c Desulfobacteria o Desulfobacterales f SURF-3 g SURF-3 s SURF-3 sp003599885 WGS QZKW01
d Bacteria p Desulfobacterota c Desulfobacteria o Desulfobacterales f SURF-3 g SURF-33 s SURF-33 sp003597945 WGS QZJS01
d Bacteria p Proteobacteria c Gammaproteobacteria o SZUA-229 f SZUA-229 g GCA-2746365 s GCA-2746365 sp002746365 WGS NVWZ01
d Bacteria p Proteobacteria c Gammaproteobacteria o SZUA-229 f SZUA-229 g SZUA-229 s SZUA-229 sp003349885 WGS QFXC01
d Bacteria p Proteobacteria c Gammaproteobacteria o Burkholderiales f Sulfuricellaceae g Sulfurirhabdus s Sulfurirhabdus autotrophica WGS SMCO01
d Bacteria p CG2-30-53-67 c CG2-30-53-67 o CG2-30-53-67 f CG2-30-53-67 g CG2-30-53-67 s CG2-30-53-67 sp001873285 WGS PFFA01
d Bacteria p Proteobacteria c Gammaproteobacteria o Thiohalobacterales f DSM-19610 g Thiogranum s Thiogranum longum WGS SMFX01
d Bacteria p Desulfobacterota c Desulfobulbia o Desulfobulbales f Desulfurivibrionaceae g CG2-30-60-27 s CG2-30-60-27 sp001873115 WGS MNYK01
d Bacteria p Proteobacteria c Gammaproteobacteria o 21-64-14 f 21-64-14 g BMS3Abin12 s BMS3Abin12 sp002897735 WGS BDTW01
d Bacteria p Proteobacteria c Gammaproteobacteria o Chromatiales f Sedimenticolaceae g Sedimenticola A s Sedimenticola A endophacoides WGS PQCP01
d Bacteria p Desulfobacterota c Dissulfuribacteria o Dissulfuribacterales f UBA5754 g UBA5754 s UBA5754 sp002419025 WGS DIFG01
d Bacteria p Desulfobacterota c Dissulfuribacteria o Dissulfuribacterales f Dissulfuribacteraceae g Dissulfuribacter s Dissulfuribacter thermophilus WGS MAGO01
d Bacteria p Desulfobacterota c Dissulfuribacteria o Dissulfuribacterales f UBA3076 g UBA3076 s UBA3076 sp003194495 WGS PQXE01
d Bacteria p Desulfobacterota c Desulfarculia o Desulfarculales f Desulfarculaceae g Dethiosulfatarculus s Dethiosulfatarculus sandiegensis WGS AZAC01
d Bacteria p Desulfobacterota c Desulfovibrionia o Desulfovibrionales f Desulfovibrionaceae g Paucidesulfovibrio s Paucidesulfovibrio gracilis WGS FUYC01
d Bacteria p Desulfobacterota c Desulfovibrionia o Desulfovibrionales f Desulfovibrionaceae g Megalodesulfovibrio s Megalodesulfovibrio gigas WGS AUBO01
d Bacteria p Desulfobacterota c Desulfovibrionia o Desulfovibrionales f Desulfovibrionaceae g Solidesulfovibrio s Solidesulfovibrio sp000177215 WGS ADFE02
d Bacteria p Desulfobacterota c Desulfobacteria o Desulfobacterales f Desulfobacteraceae g Desulfobacterium A s Desulfobacterium A vacuolatum WGS FWXY01
d Bacteria p Desulfobacterota c Desulfovibrionia o Desulfovibrionales f Desulfohalobiaceae g Desulfovermiculus s Desulfovermiculus halophilus WGS JIAK01
d Bacteria p Desulfobacterota c Desulfovibrionia o Desulfovibrionales f Desulfonatronovibrionaceae g Desulfonatronospira s Desulfonatronospira thiodismutans WGS ACJN02
d Bacteria p Desulfobacterota c Desulfovibrionia o Desulfovibrionales f Desulfonatronaceae g Desulfonatronum s Desulfonatronum thiodismutans WGS JPIK01
d Bacteria p Desulfobacterota c Desulfovibrionia o Desulfovibrionales f Desulfovibrionaceae g Alteridesulfovibrio s Alteridesulfovibrio inopinatus WGS AUBP01
d Bacteria p Desulfobacterota c Desulfovibrionia o Desulfovibrionales f Desulfoplanaceae g Desulfoplanes s Desulfoplanes formicivorans WGS BDFE01
d Bacteria p Desulfobacterota c Desulfobacteria o C00003060 f S7086C20 g S7086C20 s S7086C20 sp001751075 WGS MAVP01
d Bacteria p Desulfobacterota c Desulfobacteria o C00003060 f C00003106 g C00003106 s C00003106 sp001751015 WGS MANV01
d Bacteria p Desulfobacterota c Desulfobulbia o Desulfobulbales f Desulfocapsaceae g Desulforhopalus s Desulforhopalus sp005116645 WGS SWCM01
d Bacteria p Desulfobacterota c Desulfobulbia o Desulfobulbales f BM004 g YD12-FULL-57-12 s YD12-FULL-57-12 sp001799275 WGS MGTE01
d Bacteria p Desulfobacterota c Desulfobulbia o Desulfobulbales f Desulfurivibrionaceae g SURF-16 s SURF-16 sp003605035 WGS QZKJ01
d Bacteria p Desulfobacterota c Desulfobulbia o Desulfobulbales f Desulfurivibrionaceae g DB1 s DB1 sp001914235 WGS MQUF01
d Bacteria p Desulfobacterota c Desulfobulbia o Desulfobulbales f Desulfobulbaceae g BMS3Abin13 s BMS3Abin13 sp002897615 WGS BDTF01
d Bacteria p Desulfobacterota c Desulfobulbia o Desulfobulbales f Desulfobulbaceae g Desulfobulbus B s Desulfobulbus B propionicus A WGS PDTC01
d Bacteria p Desulfobacterota c Desulfobulbia o Desulfobulbales f Desulfobulbaceae g Desulfobulbus A s Desulfobulbus A japonicus WGS AUCV01
d Bacteria p Desulfobacterota c Desulfobulbia o Desulfobulbales f Desulfobulbaceae g Electrothrix s Electrothrix aarhusiensis WGS MTKO01
d Bacteria p Desulfobacterota c Desulfobulbia o Desulfobulbales f Desulfocapsaceae g UBA2775 s UBA2775 sp002352445 WGS DEIL01
d Bacteria p Desulfobacterota c Desulfobulbia o Desulfobulbales f Desulfocapsaceae g SZUA-575 s SZUA-575 sp003249645 WGS QKIH01
d Bacteria p Desulfobacterota c Desulfobulbia o Desulfobulbales f Desulfocapsaceae g MADRE3 s MADRE3 sp004796425 WGS QZLB01
d Bacteria p Desulfobacterota c Desulfobulbia o Desulfobulbales f Desulfocapsaceae g Desulfofustis s Desulfofustis glycolicus WGS FQXS01
d Bacteria p Desulfobacterota c Desulfobulbia o Desulfobulbales f Desulfocapsaceae g PB-SRB1 s PB-SRB1 sp003500125 WGS AVFQ01

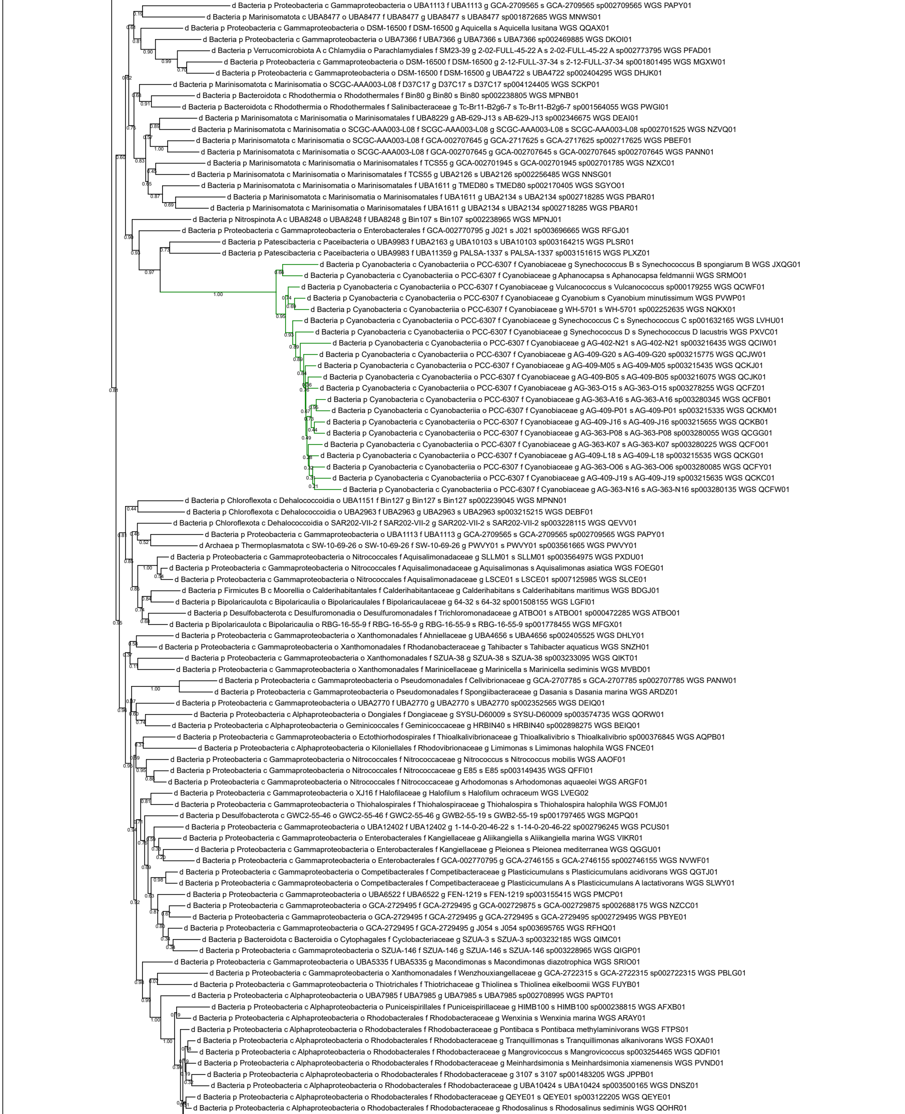
d Bacteria p Proteobacteria c Gammaproteobacteria o UBA1113 f UBA1113 g GCA-2709565 s GCA-2709565 sp002709565 WGS PAPY01
d Bacteria p Marinisomatota c UBA8477 o UBA8477 f UBA8477 g UBA8477 s UBA8477 sp001872685 WGS MNWS01
d Bacteria p Proteobacteria c Gammaproteobacteria o DSM-16500 f DSM-16500 g Aquicella s Aquicella lusitana WGS QQAX01
d Bacteria p Proteobacteria c Gammaproteobacteria o UBA7366 f UBA7366 g UBA7366 s UBA7366 sp002469885 WGS DKOI01
d Bacteria p Verrucomicrobiota A c Chlamydiia o Parachlamydiales f SM23-39 g 2-02-FULL-45-22 A s 2-02-FULL-45-22 A sp002773795 WGS PFAD01
d Bacteria p Proteobacteria c Gammaproteobacteria o DSM-16500 f DSM-16500 g 2-12-FULL-37-34 s 2-12-FULL-37-34 sp001801495 WGS MGXW01
d Bacteria p Proteobacteria c Gammaproteobacteria o DSM-16500 f DSM-16500 g UBA4722 s UBA4722 sp002404295 WGS DHJK01
d Bacteria p Marinisomatota c Marinisomatia o SCGC-AAA003-L08 f D37C17 g D37C17 s D37C17 sp004124405 WGS SCKP01
d Bacteria p Bacteroidota c Rhodothermia o Rhodothermales f Bin80 g Bin80 s Bin80 sp002238805 WGS MPNB01
d Bacteria p Bacteroidota c Rhodothermia o Rhodothermales f Salinibacteraceae g Tc-Br11-B2g6-7 s Tc-Br11-B2g6-7 sp001564055 WGS PWGI01
d Bacteria p Marinisomatota c Marinisomatia o Marinisomatales f UBA8229 g AB-629-J13 s AB-629-J13 sp002346675 WGS DEAI01
d Bacteria p Marinisomatota c Marinisomatia o SCGC-AAA003-L08 f SCGC-AAA003-L08 g SCGC-AAA003-L08 s SCGC-AAA003-L08 sp002701525 WGS NZVQ01
d Bacteria p Marinisomatota c Marinisomatia o SCGC-AAA003-L08 f GCA-002707645 g GCA-2717625 s GCA-2717625 sp002717625 WGS PBEF01
d Bacteria p Marinisomatota c Marinisomatia o SCGC-AAA003-L08 f GCA-002707645 g GCA-002707645 s GCA-002707645 sp002707645 WGS PANN01
d Bacteria p Marinisomatota c Marinisomatia o Marinisomatales f TCS55 g GCA-002701945 s GCA-002701945 sp002701785 WGS NZXC01
d Bacteria p Marinisomatota c Marinisomatia o Marinisomatales f TCS55 g UBA2126 s UBA2126 sp002256485 WGS NNSG01
d Bacteria p Marinisomatota c Marinisomatia o Marinisomatales f UBA1611 g TMED80 s TMED80 sp002170405 WGS SGYO01
d Bacteria p Marinisomatota c Marinisomatia o Marinisomatales f UBA1611 g UBA2134 s UBA2134 sp002718285 WGS PBAR01
d Bacteria p Marinisomatota c Marinisomatia o Marinisomatales f UBA1611 g UBA2134 s UBA2134 sp002718285 WGS PBAR01
d Bacteria p Nitrospinota A c UBA8248 o UBA8248 f UBA8248 g Bin107 s Bin107 sp002238965 WGS MPNJ01
d Bacteria p Proteobacteria c Gammaproteobacteria o Enterobacterales f GCA-002770795 g J021 s J021 sp003696665 WGS RFGJ01
d Bacteria p Patescibacteria c Paceibacteria o UBA9983 f UBA2163 g UBA10103 s UBA10103 sp003164215 WGS PLSR01
d Bacteria p Patescibacteria c Paceibacteria o UBA9983 f UBA11359 g PALSA-1337 s PALSA-1337 sp003151615 WGS PLXZ01
d Bacteria p Cyanobacteria c Cyanobacteriia o PCC-6307 f Cyanobiaceae g Synechococcus B s Synechococcus B spongiarum B WGS JXQG01
d Bacteria p Cyanobacteria c Cyanobacteriia o PCC-6307 f Cyanobiaceae g Aphanocapsa s Aphanocapsa feldmannii WGS SRMO01
d Bacteria p Cyanobacteria c Cyanobacteriia o PCC-6307 f Cyanobiaceae g Vulcanococcus s Vulcanococcus sp000179255 WGS QCWF01
d Bacteria p Cyanobacteria c Cyanobacteriia o PCC-6307 f Cyanobiaceae g Cyanobium s Cyanobium minutissimum WGS PVWP01
d Bacteria p Cyanobacteria c Cyanobacteriia o PCC-6307 f Cyanobiaceae g WH-5701 s WH-5701 sp002252635 WGS NQKX01
d Bacteria p Cyanobacteria c Cyanobacteriia o PCC-6307 f Cyanobiaceae g Synechococcus C s Synechococcus C sp001632165 WGS LVHU01
d Bacteria p Cyanobacteria c Cyanobacteriia o PCC-6307 f Cyanobiaceae g Synechococcus D s Synechococcus D lacustris WGS PXVC01
d Bacteria p Cyanobacteria c Cyanobacteriia o PCC-6307 f Cyanobiaceae g AG-402-N21 s AG-402-N21 sp003216435 WGS QCIW01
d Bacteria p Cyanobacteria c Cyanobacteriia o PCC-6307 f Cyanobiaceae g AG-409-G20 s AG-409-G20 sp003215775 WGS QCJW01
d Bacteria p Cyanobacteria c Cyanobacteriia o PCC-6307 f Cyanobiaceae g AG-409-M05 s AG-409-M05 sp003215435 WGS QCKJ01
d Bacteria p Cyanobacteria c Cyanobacteriia o PCC-6307 f Cyanobiaceae g AG-409-B05 s AG-409-B05 sp003216075 WGS QCJK01
d Bacteria p Cyanobacteria c Cyanobacteriia o PCC-6307 f Cyanobiaceae g AG-363-O15 s AG-363-O15 sp003278255 WGS QCFZ01
d Bacteria p Cyanobacteria c Cyanobacteriia o PCC-6307 f Cyanobiaceae g AG-363-A16 s AG-363-A16 sp003280345 WGS QCFB01
d Bacteria p Cyanobacteria c Cyanobacteriia o PCC-6307 f Cyanobiaceae g AG-409-P01 s AG-409-P01 sp003215335 WGS QCKM01
d Bacteria p Cyanobacteria c Cyanobacteriia o PCC-6307 f Cyanobiaceae g AG-409-J16 s AG-409-J16 sp003215655 WGS QCKB01
d Bacteria p Cyanobacteria c Cyanobacteriia o PCC-6307 f Cyanobiaceae g AG-363-P08 s AG-363-P08 sp003280055 WGS QCGG01
d Bacteria p Cyanobacteria c Cyanobacteriia o PCC-6307 f Cyanobiaceae g AG-363-K07 s AG-363-K07 sp003280225 WGS QCFO01
d Bacteria p Cyanobacteria c Cyanobacteriia o PCC-6307 f Cyanobiaceae g AG-409-L18 s AG-409-L18 sp003215535 WGS QCKG01
d Bacteria p Cyanobacteria c Cyanobacteriia o PCC-6307 f Cyanobiaceae g AG-363-O06 s AG-363-O06 sp003280085 WGS QCFY01
d Bacteria p Cyanobacteria c Cyanobacteriia o PCC-6307 f Cyanobiaceae g AG-409-J19 s AG-409-J19 sp003215635 WGS QCKC01
d Bacteria p Cyanobacteria c Cyanobacteriia o PCC-6307 f Cyanobiaceae g AG-363-N16 s AG-363-N16 sp003280135 WGS QCFW01
d Bacteria p Chloroflexota c Dehalococcoidia o UBA1151 f Bin127 g Bin127 s Bin127 sp002239045 WGS MPNN01
d Bacteria p Chloroflexota c Dehalococcoidia o UBA2963 f UBA2963 g UBA2963 s UBA2963 sp003215215 WGS DEBF01
d Bacteria p Chloroflexota c Dehalococcoidia o SAR202-VII-2 f SAR202-VII-2 g SAR202-VII-2 s SAR202-VII-2 sp003228115 WGS QEVV01
d Bacteria p Proteobacteria c Gammaproteobacteria o UBA1113 f UBA1113 g GCA-2709565 s GCA-2709565 sp002709565 WGS PAPY01
d Archaea p Thermoplasmatota c SW-10-69-26 o SW-10-69-26 f SW-10-69-26 g PWVY01 s PWVY01 sp003561665 WGS PWVY01
d Bacteria p Proteobacteria c Gammaproteobacteria o Nitrococcales f Aquisalimonadaceae g SLLM01 s SLLM01 sp003564975 WGS PXDU01
d Bacteria p Proteobacteria c Gammaproteobacteria o Nitrococcales f Aquisalimonadaceae g Aquisalimonas s Aquisalimonas asiatica WGS FOEG01
d Bacteria p Proteobacteria c Gammaproteobacteria o Nitrococcales f Aquisalimonadaceae g LSCE01 s LSCE01 sp007125985 WGS SLCE01
d Bacteria p Firmicutes B c Moorellia o Calderihabitantales f Calderihabitantaceae g Calderihabitans s Calderihabitans maritimus WGS BDGJ01
d Bacteria p Bipolaricaulota c Bipolaricaulia o Bipolaricaulales f Bipolaricaulaceae g 64-32 s 64-32 sp001508155 WGS LGFI01
d Bacteria p Desulfobacterota c Desulfuromonadia o Desulfuromonadales f Trichloromonadaceae g ATBO01 s ATBO01 sp000472285 WGS ATBO01
d Bacteria p Bipolaricaulota c Bipolaricaulia o RBG-16-55-9 f RBG-16-55-9 g RBG-16-55-9 s RBG-16-55-9 sp001778455 WGS MFGX01
d Bacteria p Proteobacteria c Gammaproteobacteria o Xanthomonadales f Ahniellaceae g UBA4656 s UBA4656 sp002405525 WGS DHLY01
d Bacteria p Proteobacteria c Gammaproteobacteria o Xanthomonadales f Rhodanobacteraceae g Tahibacter s Tahibacter aquaticus WGS SNZH01
d Bacteria p Proteobacteria c Gammaproteobacteria o Xanthomonadales f SZUA-38 g SZUA-38 s SZUA-38 sp003233095 WGS QIKT01
d Bacteria p Proteobacteria c Gammaproteobacteria o Xanthomonadales f Marinicellaceae g Marinicella s Marinicella sediminis WGS MVBD01
d Bacteria p Proteobacteria c Gammaproteobacteria o Pseudomonadales f Cellvibrionaceae g GCA-2707785 s GCA-2707785 sp002707785 WGS PANW01
d Bacteria p Proteobacteria c Gammaproteobacteria o Pseudomonadales f Spongiibacteraceae g Dasania s Dasania marina WGS ARDZ01
d Bacteria p Proteobacteria c Gammaproteobacteria o UBA2770 f UBA2770 g UBA2770 s UBA2770 sp002352565 WGS DEIQ01
d Bacteria p Proteobacteria c Alphaproteobacteria o Dongiales f Dongiaceae g SYSU-D60009 s SYSU-D60009 sp003574735 WGS QORW01
d Bacteria p Proteobacteria c Alphaproteobacteria o Geminicoccales f Geminicoccaceae g HRBIN40 s HRBIN40 sp002898275 WGS BEIQ01
d Bacteria p Proteobacteria c Gammaproteobacteria o Ectothiorhodospirales f Thioalkalivibrionaceae g Thioalkalivibrio s Thioalkalivibrio sp000376845 WGS AQPB01
d Bacteria p Proteobacteria c Alphaproteobacteria o Kiloniellales f Rhodovibrionaceae g Limimonas s Limimonas halophila WGS FNCE01
d Bacteria p Proteobacteria c Gammaproteobacteria o Nitrococcales f Nitrococcaceae g Nitrococcus s Nitrococcus mobilis WGS AAOF01
d Bacteria p Proteobacteria c Gammaproteobacteria o Nitrococcales f Nitrococcaceae g E85 s E85 sp003149435 WGS QFFI01
d Bacteria p Proteobacteria c Gammaproteobacteria o Nitrococcales f Nitrococcaceae g Arhodomonas s Arhodomonas aquaeolei WGS ARGF01
d Bacteria p Proteobacteria c Gammaproteobacteria o XJ16 f Halofilaceae g Halofilum s Halofilum ochraceum WGS LVEG02
d Bacteria p Proteobacteria c Gammaproteobacteria o Thiohalospirales f Thiohalospiraceae g Thiohalospira s Thiohalospira halophila WGS FOMJ01
d Bacteria p Desulfobacterota c GWC2-55-46 o GWC2-55-46 f GWC2-55-46 g GWB2-55-19 s GWB2-55-19 sp001797465 WGS MGPQ01
d Bacteria p Proteobacteria c Gammaproteobacteria o UBA12402 f UBA12402 g 1-14-0-20-46-22 s 1-14-0-20-46-22 sp002796245 WGS PCUS01
d Bacteria p Proteobacteria c Gammaproteobacteria o Enterobacterales f Kangiellaceae g Aliikangiella s Aliikangiella marina WGS VIKR01
d Bacteria p Proteobacteria c Gammaproteobacteria o Enterobacterales f Kangiellaceae g Pleionea s Pleionea mediterranea WGS QGGU01
d Bacteria p Proteobacteria c Gammaproteobacteria o Enterobacterales f GCA-002770795 g GCA-2746155 s GCA-2746155 sp002746155 WGS NVWF01
d Bacteria p Proteobacteria c Gammaproteobacteria o Competibacterales f Competibacteraceae g Plasticicumulans s Plasticicumulans acidivorans WGS QGTJ01
d Bacteria p Proteobacteria c Gammaproteobacteria o Competibacterales f Competibacteraceae g Plasticicumulans A s Plasticicumulans A lactativorans WGS SLWY01
d Bacteria p Proteobacteria c Gammaproteobacteria o UBA6522 f UBA6522 g FEN-1219 s FEN-1219 sp003155415 WGS PMCP01
d Bacteria p Proteobacteria c Gammaproteobacteria o GCA-2729495 f GCA-2729495 g GCA-002729875 s GCA-002729875 sp002688175 WGS NZCC01
d Bacteria p Proteobacteria c Gammaproteobacteria o GCA-2729495 f GCA-2729495 g GCA-2729495 s GCA-2729495 sp002729495 WGS PBYE01
d Bacteria p Proteobacteria c Gammaproteobacteria o GCA-2729495 f GCA-2729495 g J054 s J054 sp003695765 WGS RFHQ01
d Bacteria p Bacteroidota c Bacteroidia o Cytophagales f Cyclobacteriaceae g SZUA-3 s SZUA-3 sp003232185 WGS QIMC01
d Bacteria p Proteobacteria c Gammaproteobacteria o SZUA-146 f SZUA-146 g SZUA-146 s SZUA-146 sp003228965 WGS QIGP01
d Bacteria p Proteobacteria c Gammaproteobacteria o UBA5335 f UBA5335 g Macondimonas s Macondimonas diazotrophica WGS SRIO01
d Bacteria p Proteobacteria c Gammaproteobacteria o Xanthomonadales f Wenzhouxiangellaceae g GCA-2722315 s GCA-2722315 sp002722315 WGS PBLG01
d Bacteria p Proteobacteria c Gammaproteobacteria o Thiotrichales f Thiotrichaceae g Thiolinea s Thiolinea eikelboomii WGS FUYB01
d Bacteria p Proteobacteria c Alphaproteobacteria o UBA7985 f UBA7985 g UBA7985 s UBA7985 sp002708995 WGS PAPT01
d Bacteria p Proteobacteria c Alphaproteobacteria o Puniceispirillales f Puniceispirillaceae g HIMB100 s HIMB100 sp000238815 WGS AFXB01
d Bacteria p Proteobacteria c Alphaproteobacteria o Rhodobacterales f Rhodobacteraceae g Wenxinia s Wenxinia marina WGS ARAY01
d Bacteria p Proteobacteria c Alphaproteobacteria o Rhodobacterales f Rhodobacteraceae g Pontibaca s Pontibaca methylaminivorans WGS FTPS01
d Bacteria p Proteobacteria c Alphaproteobacteria o Rhodobacterales f Rhodobacteraceae g Tranquillimonas s Tranquillimonas alkanivorans WGS FOXA01
d Bacteria p Proteobacteria c Alphaproteobacteria o Rhodobacterales f Rhodobacteraceae g Mangrovicoccus s Mangrovicoccus sp003254465 WGS QDFI01
d Bacteria p Proteobacteria c Alphaproteobacteria o Rhodobacterales f Rhodobacteraceae g Meinhardsimonia s Meinhardsimonia xiamenensis WGS PVND01
d Bacteria p Proteobacteria c Alphaproteobacteria o Rhodobacterales f Rhodobacteraceae g 3107 s 3107 sp001483205 WGS JPPB01
d Bacteria p Proteobacteria c Alphaproteobacteria o Rhodobacterales f Rhodobacteraceae g UBA10424 s UBA10424 sp003500165 WGS DNSZ01
d Bacteria p Proteobacteria c Alphaproteobacteria o Rhodobacterales f Rhodobacteraceae g QEYE01 s QEYE01 sp003122205 WGS QEYE01
d Bacteria p Proteobacteria c Alphaproteobacteria o Rhodobacterales f Rhodobacteraceae g Rhodosalinus s Rhodosalinus sediminis WGS QOHR01

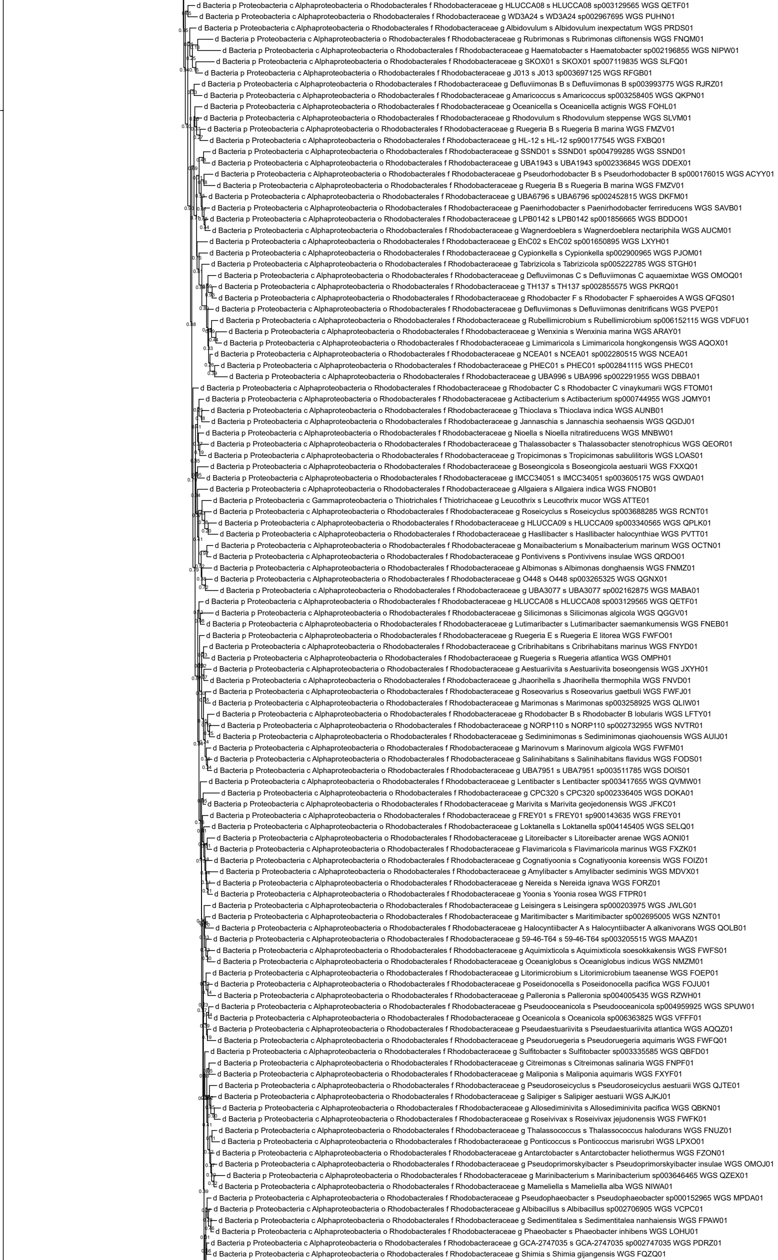
d Bacteria p Proteobacteria c Alphaproteobacteria o Rhodobacterales f Rhodobacteraceae g HLUCCA08 s HLUCCA08 sp003129565 WGS QETF01
d Bacteria p Proteobacteria c Alphaproteobacteria o Rhodobacterales f Rhodobacteraceae g WD3A24 s WD3A24 sp002967695 WGS PUHN01
d Bacteria p Proteobacteria c Alphaproteobacteria o Rhodobacterales f Rhodobacteraceae g Albidovulum s Albidovulum inexpectatum WGS PRDS01
d Bacteria p Proteobacteria c Alphaproteobacteria o Rhodobacterales f Rhodobacteraceae g Rubrimonas s Rubrimonas cliftonensis WGS FNQM01
d Bacteria p Proteobacteria c Alphaproteobacteria o Rhodobacterales f Rhodobacteraceae g Haematobacter s Haematobacter sp002196855 WGS NIPW01
d Bacteria p Proteobacteria c Alphaproteobacteria o Rhodobacterales f Rhodobacteraceae g SKOX01 s SKOX01 sp007119835 WGS SLFQ01
d Bacteria p Proteobacteria c Alphaproteobacteria o Rhodobacterales f Rhodobacteraceae g J013 s J013 sp003697125 WGS RFGB01
d Bacteria p Proteobacteria c Alphaproteobacteria o Rhodobacterales f Rhodobacteraceae g Defluviimonas B s Defluviimonas B sp003993775 WGS RJRZ01
d Bacteria p Proteobacteria c Alphaproteobacteria o Rhodobacterales f Rhodobacteraceae g Amaricoccus s Amaricoccus sp003258405 WGS QKPN01
d Bacteria p Proteobacteria c Alphaproteobacteria o Rhodobacterales f Rhodobacteraceae g Oceanicella s Oceanicella actignis WGS FOHL01
d Bacteria p Proteobacteria c Alphaproteobacteria o Rhodobacterales f Rhodobacteraceae g Rhodovulum s Rhodovulum steppense WGS SLVM01
d Bacteria p Proteobacteria c Alphaproteobacteria o Rhodobacterales f Rhodobacteraceae g Ruegeria B s Ruegeria B marina WGS FMZV01
d Bacteria p Proteobacteria c Alphaproteobacteria o Rhodobacterales f Rhodobacteraceae g HL-12 s HL-12 sp900177545 WGS FXBQ01
d Bacteria p Proteobacteria c Alphaproteobacteria o Rhodobacterales f Rhodobacteraceae g SSND01 s SSND01 sp004799285 WGS SSND01
d Bacteria p Proteobacteria c Alphaproteobacteria o Rhodobacterales f Rhodobacteraceae g UBA1943 s UBA1943 sp002336845 WGS DDEX01
d Bacteria p Proteobacteria c Alphaproteobacteria o Rhodobacterales f Rhodobacteraceae g Pseudorhodobacter B s Pseudorhodobacter B sp000176015 WGS ACYY01
d Bacteria p Proteobacteria c Alphaproteobacteria o Rhodobacterales f Rhodobacteraceae g Ruegeria B s Ruegeria B marina WGS FMZV01
d Bacteria p Proteobacteria c Alphaproteobacteria o Rhodobacterales f Rhodobacteraceae g UBA6796 s UBA6796 sp002452815 WGS DKFM01
d Bacteria p Proteobacteria c Alphaproteobacteria o Rhodobacterales f Rhodobacteraceae g Paenirhodobacter s Paenirhodobacter ferrireducens WGS SAVB01
d Bacteria p Proteobacteria c Alphaproteobacteria o Rhodobacterales f Rhodobacteraceae g LPB0142 s LPB0142 sp001856665 WGS BDDO01
d Bacteria p Proteobacteria c Alphaproteobacteria o Rhodobacterales f Rhodobacteraceae g Wagnerdoeblera s Wagnerdoeblera nectariphila WGS AUCM01
d Bacteria p Proteobacteria c Alphaproteobacteria o Rhodobacterales f Rhodobacteraceae g EhC02 s EhC02 sp001650895 WGS LXYH01
d Bacteria p Proteobacteria c Alphaproteobacteria o Rhodobacterales f Rhodobacteraceae g Cypionkella s Cypionkella sp002900965 WGS PJOM01
d Bacteria p Proteobacteria c Alphaproteobacteria o Rhodobacterales f Rhodobacteraceae g Tabrizicola s Tabrizicola sp005222785 WGS STGH01
d Bacteria p Proteobacteria c Alphaproteobacteria o Rhodobacterales f Rhodobacteraceae g Defluviimonas C s Defluviimonas C aquaemixtae WGS OMOQ01
d Bacteria p Proteobacteria c Alphaproteobacteria o Rhodobacterales f Rhodobacteraceae g TH137 s TH137 sp002855575 WGS PKRQ01
d Bacteria p Proteobacteria c Alphaproteobacteria o Rhodobacterales f Rhodobacteraceae g Rhodobacter F s Rhodobacter F sphaeroides A WGS QFQS01
d Bacteria p Proteobacteria c Alphaproteobacteria o Rhodobacterales f Rhodobacteraceae g Defluviimonas s Defluviimonas denitrificans WGS PVEP01
d Bacteria p Proteobacteria c Alphaproteobacteria o Rhodobacterales f Rhodobacteraceae g Rubellimicrobium s Rubellimicrobium sp006152115 WGS VDFU01
d Bacteria p Proteobacteria c Alphaproteobacteria o Rhodobacterales f Rhodobacteraceae g Wenxinia s Wenxinia marina WGS ARAY01
d Bacteria p Proteobacteria c Alphaproteobacteria o Rhodobacterales f Rhodobacteraceae g Limimaricola s Limimaricola hongkongensis WGS AQOX01
d Bacteria p Proteobacteria c Alphaproteobacteria o Rhodobacterales f Rhodobacteraceae g NCEA01 s NCEA01 sp002280515 WGS NCEA01
d Bacteria p Proteobacteria c Alphaproteobacteria o Rhodobacterales f Rhodobacteraceae g PHEC01 s PHEC01 sp002841115 WGS PHEC01
d Bacteria p Proteobacteria c Alphaproteobacteria o Rhodobacterales f Rhodobacteraceae g UBA996 s UBA996 sp002291955 WGS DBBA01
d Bacteria p Proteobacteria c Alphaproteobacteria o Rhodobacterales f Rhodobacteraceae g Rhodobacter C s Rhodobacter C vinaykumarii WGS FTOM01
d Bacteria p Proteobacteria c Alphaproteobacteria o Rhodobacterales f Rhodobacteraceae g Actibacterium s Actibacterium sp000744955 WGS JQMY01
d Bacteria p Proteobacteria c Alphaproteobacteria o Rhodobacterales f Rhodobacteraceae g Thioclava s Thioclava indica WGS AUNB01
d Bacteria p Proteobacteria c Alphaproteobacteria o Rhodobacterales f Rhodobacteraceae g Jannaschia s Jannaschia seohaensis WGS QGDJ01
d Bacteria p Proteobacteria c Alphaproteobacteria o Rhodobacterales f Rhodobacteraceae g Nioella s Nioella nitratireducens WGS MNBW01
d Bacteria p Proteobacteria c Alphaproteobacteria o Rhodobacterales f Rhodobacteraceae g Thalassobacter s Thalassobacter stenotrophicus WGS QEOR01
d Bacteria p Proteobacteria c Alphaproteobacteria o Rhodobacterales f Rhodobacteraceae g Tropicimonas s Tropicimonas sabulilitoris WGS LOAS01
d Bacteria p Proteobacteria c Alphaproteobacteria o Rhodobacterales f Rhodobacteraceae g Boseongicola s Boseongicola aestuarii WGS FXXQ01
d Bacteria p Proteobacteria c Alphaproteobacteria o Rhodobacterales f Rhodobacteraceae g IMCC34051 s IMCC34051 sp003605175 WGS QWDA01
d Bacteria p Proteobacteria c Alphaproteobacteria o Rhodobacterales f Rhodobacteraceae g Allgaiera s Allgaiera indica WGS FNOB01
d Bacteria p Proteobacteria c Gammaproteobacteria o Thiotrichales f Thiotrichaceae g Leucothrix s Leucothrix mucor WGS ATTE01
d Bacteria p Proteobacteria c Alphaproteobacteria o Rhodobacterales f Rhodobacteraceae g Roseicyclus s Roseicyclus sp003688285 WGS RCNT01
d Bacteria p Proteobacteria c Alphaproteobacteria o Rhodobacterales f Rhodobacteraceae g HLUCCA09 s HLUCCA09 sp003340565 WGS QPLK01
d Bacteria p Proteobacteria c Alphaproteobacteria o Rhodobacterales f Rhodobacteraceae g Hasllibacter s Hasllibacter halocynthiae WGS PVTT01
d Bacteria p Proteobacteria c Alphaproteobacteria o Rhodobacterales f Rhodobacteraceae g Monaibacterium s Monaibacterium marinum WGS OCTN01
d Bacteria p Proteobacteria c Alphaproteobacteria o Rhodobacterales f Rhodobacteraceae g Pontivivens s Pontivivens insulae WGS QRDO01
d Bacteria p Proteobacteria c Alphaproteobacteria o Rhodobacterales f Rhodobacteraceae g Albimonas s Albimonas donghaensis WGS FNMZ01
d Bacteria p Proteobacteria c Alphaproteobacteria o Rhodobacterales f Rhodobacteraceae g O448 s O448 sp003265325 WGS QGNX01
d Bacteria p Proteobacteria c Alphaproteobacteria o Rhodobacterales f Rhodobacteraceae g UBA3077 s UBA3077 sp002162875 WGS MABA01
d Bacteria p Proteobacteria c Alphaproteobacteria o Rhodobacterales f Rhodobacteraceae g HLUCCA08 s HLUCCA08 sp003129565 WGS QETF01
d Bacteria p Proteobacteria c Alphaproteobacteria o Rhodobacterales f Rhodobacteraceae g Silicimonas s Silicimonas algicola WGS QGGV01
d Bacteria p Proteobacteria c Alphaproteobacteria o Rhodobacterales f Rhodobacteraceae g Lutimaribacter s Lutimaribacter saemankumensis WGS FNEB01
d Bacteria p Proteobacteria c Alphaproteobacteria o Rhodobacterales f Rhodobacteraceae g Ruegeria E s Ruegeria E litorea WGS FWFO01
d Bacteria p Proteobacteria c Alphaproteobacteria o Rhodobacterales f Rhodobacteraceae g Cribrihabitans s Cribrihabitans marinus WGS FNYD01
d Bacteria p Proteobacteria c Alphaproteobacteria o Rhodobacterales f Rhodobacteraceae g Ruegeria s Ruegeria atlantica WGS OMPH01
d Bacteria p Proteobacteria c Alphaproteobacteria o Rhodobacterales f Rhodobacteraceae g Aestuariivita s Aestuariivita boseongensis WGS JXYH01
d Bacteria p Proteobacteria c Alphaproteobacteria o Rhodobacterales f Rhodobacteraceae g Jhaorihella s Jhaorihella thermophila WGS FNVD01
d Bacteria p Proteobacteria c Alphaproteobacteria o Rhodobacterales f Rhodobacteraceae g Roseovarius s Roseovarius gaetbuli WGS FWFJ01
d Bacteria p Proteobacteria c Alphaproteobacteria o Rhodobacterales f Rhodobacteraceae g Marimonas s Marimonas sp003258925 WGS QLIW01
d Bacteria p Proteobacteria c Alphaproteobacteria o Rhodobacterales f Rhodobacteraceae g Rhodobacter B s Rhodobacter B lobularis WGS LFTY01
d Bacteria p Proteobacteria c Alphaproteobacteria o Rhodobacterales f Rhodobacteraceae g NORP110 s NORP110 sp002732955 WGS NVTR01
d Bacteria p Proteobacteria c Alphaproteobacteria o Rhodobacterales f Rhodobacteraceae g Sediminimonas s Sediminimonas qiaohouensis WGS AUIJ01
d Bacteria p Proteobacteria c Alphaproteobacteria o Rhodobacterales f Rhodobacteraceae g Marinovum s Marinovum algicola WGS FWFM01
d Bacteria p Proteobacteria c Alphaproteobacteria o Rhodobacterales f Rhodobacteraceae g Salinihabitans s Salinihabitans flavidus WGS FODS01
d Bacteria p Proteobacteria c Alphaproteobacteria o Rhodobacterales f Rhodobacteraceae g UBA7951 s UBA7951 sp003511785 WGS DOIS01
d Bacteria p Proteobacteria c Alphaproteobacteria o Rhodobacterales f Rhodobacteraceae g Lentibacter s Lentibacter sp003417655 WGS QVMW01
d Bacteria p Proteobacteria c Alphaproteobacteria o Rhodobacterales f Rhodobacteraceae g CPC320 s CPC320 sp002336405 WGS DOKA01
d Bacteria p Proteobacteria c Alphaproteobacteria o Rhodobacterales f Rhodobacteraceae g Marivita s Marivita geojedonensis WGS JFKC01
d Bacteria p Proteobacteria c Alphaproteobacteria o Rhodobacterales f Rhodobacteraceae g FREY01 s FREY01 sp900143635 WGS FREY01
d Bacteria p Proteobacteria c Alphaproteobacteria o Rhodobacterales f Rhodobacteraceae g Loktanella s Loktanella sp004145405 WGS SELQ01
d Bacteria p Proteobacteria c Alphaproteobacteria o Rhodobacterales f Rhodobacteraceae g Litoreibacter s Litoreibacter arenae WGS AONI01
d Bacteria p Proteobacteria c Alphaproteobacteria o Rhodobacterales f Rhodobacteraceae g Flavimaricola s Flavimaricola marinus WGS FXZK01
d Bacteria p Proteobacteria c Alphaproteobacteria o Rhodobacterales f Rhodobacteraceae g Cognatiyoonia s Cognatiyoonia koreensis WGS FOIZ01
d Bacteria p Proteobacteria c Alphaproteobacteria o Rhodobacterales f Rhodobacteraceae g Amylibacter s Amylibacter sediminis WGS MDVX01
d Bacteria p Proteobacteria c Alphaproteobacteria o Rhodobacterales f Rhodobacteraceae g Nereida s Nereida ignava WGS FORZ01
d Bacteria p Proteobacteria c Alphaproteobacteria o Rhodobacterales f Rhodobacteraceae g Yoonia s Yoonia rosea WGS FTPR01
d Bacteria p Proteobacteria c Alphaproteobacteria o Rhodobacterales f Rhodobacteraceae g Leisingera s Leisingera sp000203975 WGS JWLG01
d Bacteria p Proteobacteria c Alphaproteobacteria o Rhodobacterales f Rhodobacteraceae g Maritimibacter s Maritimibacter sp002695005 WGS NZNT01
d Bacteria p Proteobacteria c Alphaproteobacteria o Rhodobacterales f Rhodobacteraceae g Halocyntiibacter A s Halocyntiibacter A alkanivorans WGS QOLB01
d Bacteria p Proteobacteria c Alphaproteobacteria o Rhodobacterales f Rhodobacteraceae g 59-46-T64 s 59-46-T64 sp003205515 WGS MAAZ01
d Bacteria p Proteobacteria c Alphaproteobacteria o Rhodobacterales f Rhodobacteraceae g Aquimixticola s Aquimixticola soesokkakensis WGS FWFS01
d Bacteria p Proteobacteria c Alphaproteobacteria o Rhodobacterales f Rhodobacteraceae g Oceaniglobus s Oceaniglobus indicus WGS NMZM01
d Bacteria p Proteobacteria c Alphaproteobacteria o Rhodobacterales f Rhodobacteraceae g Litorimicrobium s Litorimicrobium taeanense WGS FOEP01
d Bacteria p Proteobacteria c Alphaproteobacteria o Rhodobacterales f Rhodobacteraceae g Poseidonocella s Poseidonocella pacifica WGS FOJU01
d Bacteria p Proteobacteria c Alphaproteobacteria o Rhodobacterales f Rhodobacteraceae g Palleronia s Palleronia sp004005435 WGS RZWH01
d Bacteria p Proteobacteria c Alphaproteobacteria o Rhodobacterales f Rhodobacteraceae g Pseudooceanicola s Pseudooceanicola sp004959925 WGS SPUW01
d Bacteria p Proteobacteria c Alphaproteobacteria o Rhodobacterales f Rhodobacteraceae g Oceanicola s Oceanicola sp006363825 WGS VFFF01
d Bacteria p Proteobacteria c Alphaproteobacteria o Rhodobacterales f Rhodobacteraceae g Pseudaestuariivita s Pseudaestuariivita atlantica WGS AQQZ01
d Bacteria p Proteobacteria c Alphaproteobacteria o Rhodobacterales f Rhodobacteraceae g Pseudoruegeria s Pseudoruegeria aquimaris WGS FWFQ01
d Bacteria p Proteobacteria c Alphaproteobacteria o Rhodobacterales f Rhodobacteraceae g Sulfitobacter s Sulfitobacter sp003335585 WGS QBFD01
d Bacteria p Proteobacteria c Alphaproteobacteria o Rhodobacterales f Rhodobacteraceae g Citreimonas s Citreimonas salinaria WGS FNPF01
d Bacteria p Proteobacteria c Alphaproteobacteria o Rhodobacterales f Rhodobacteraceae g Maliponia s Maliponia aquimaris WGS FXYF01
d Bacteria p Proteobacteria c Alphaproteobacteria o Rhodobacterales f Rhodobacteraceae g Pseudoroseicyclus s Pseudoroseicyclus aestuarii WGS QJTE01
d Bacteria p Proteobacteria c Alphaproteobacteria o Rhodobacterales f Rhodobacteraceae g Salipiger s Salipiger aestuarii WGS AJKJ01
d Bacteria p Proteobacteria c Alphaproteobacteria o Rhodobacterales f Rhodobacteraceae g Allosediminivita s Allosediminivita pacifica WGS QBKN01
d Bacteria p Proteobacteria c Alphaproteobacteria o Rhodobacterales f Rhodobacteraceae g Roseivivax s Roseivivax jejudonensis WGS FWFK01
d Bacteria p Proteobacteria c Alphaproteobacteria o Rhodobacterales f Rhodobacteraceae g Thalassococcus s Thalassococcus halodurans WGS FNUZ01
d Bacteria p Proteobacteria c Alphaproteobacteria o Rhodobacterales f Rhodobacteraceae g Ponticoccus s Ponticoccus marisrubri WGS LPXO01
d Bacteria p Proteobacteria c Alphaproteobacteria o Rhodobacterales f Rhodobacteraceae g Antarctobacter s Antarctobacter heliothermus WGS FZON01
d Bacteria p Proteobacteria c Alphaproteobacteria o Rhodobacterales f Rhodobacteraceae g Pseudoprimorskyibacter s Pseudoprimorskyibacter insulae WGS OMOJ01
d Bacteria p Proteobacteria c Alphaproteobacteria o Rhodobacterales f Rhodobacteraceae g Marinibacterium s Marinibacterium sp003646465 WGS QZEX01
d Bacteria p Proteobacteria c Alphaproteobacteria o Rhodobacterales f Rhodobacteraceae g Mameliella s Mameliella alba WGS NIWA01
d Bacteria p Proteobacteria c Alphaproteobacteria o Rhodobacterales f Rhodobacteraceae g Pseudophaeobacter s Pseudophaeobacter sp000152965 WGS MPDA01
d Bacteria p Proteobacteria c Alphaproteobacteria o Rhodobacterales f Rhodobacteraceae g Albibacillus s Albibacillus sp002706905 WGS VCPC01
d Bacteria p Proteobacteria c Alphaproteobacteria o Rhodobacterales f Rhodobacteraceae g Sedimentitalea s Sedimentitalea nanhaiensis WGS FPAW01
d Bacteria p Proteobacteria c Alphaproteobacteria o Rhodobacterales f Rhodobacteraceae g Phaeobacter s Phaeobacter inhibens WGS LOHU01
d Bacteria p Proteobacteria c Alphaproteobacteria o Rhodobacterales f Rhodobacteraceae g GCA-2747035 s GCA-2747035 sp002747035 WGS PDRZ01
d Bacteria p Proteobacteria c Alphaproteobacteria o Rhodobacterales f Rhodobacteraceae g Shimia s Shimia gijangensis WGS FQZQ01

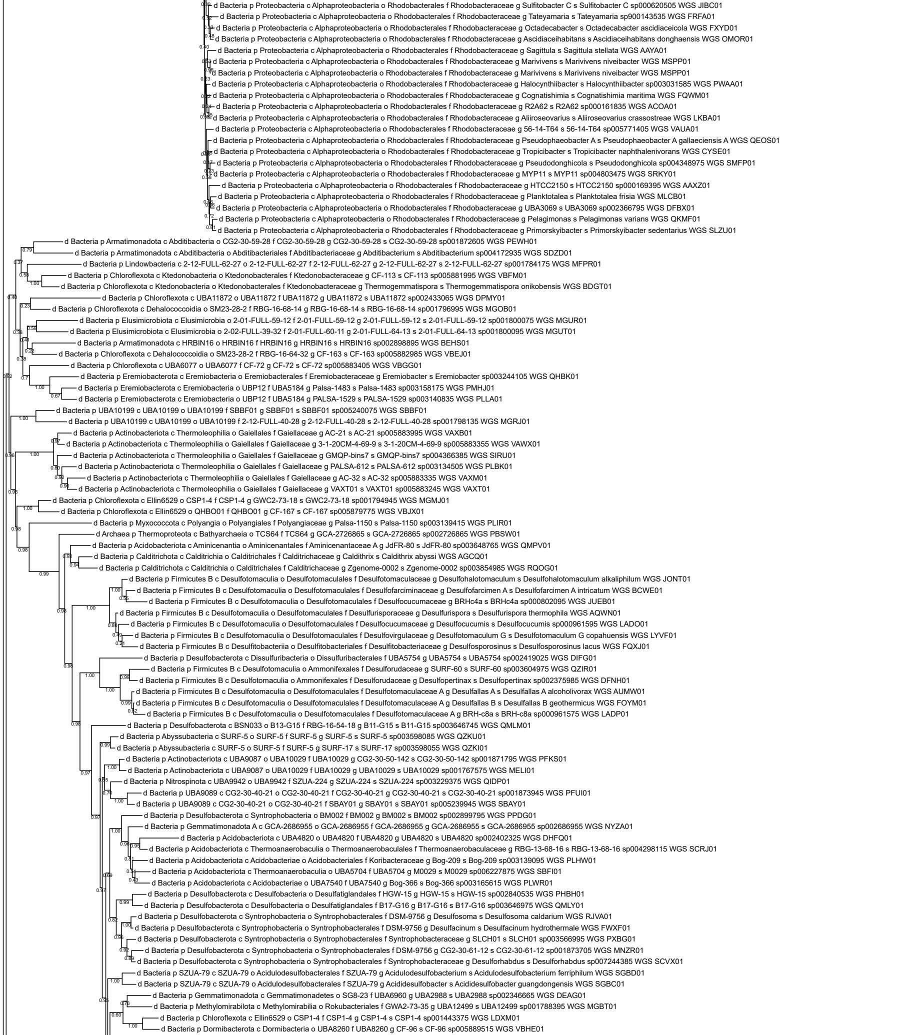
d Bacteria p Proteobacteria c Alphaproteobacteria o Rhodobacterales f Rhodobacteraceae g Sulfitobacter C s Sulfitobacter C sp000620505 WGS JIBC01
d Bacteria p Proteobacteria c Alphaproteobacteria o Rhodobacterales f Rhodobacteraceae g Tateyamaria s Tateyamaria sp900143535 WGS FRFA01
d Bacteria p Proteobacteria c Alphaproteobacteria o Rhodobacterales f Rhodobacteraceae g Octadecabacter s Octadecabacter ascidiaceicola WGS FXYD01
d Bacteria p Proteobacteria c Alphaproteobacteria o Rhodobacterales f Rhodobacteraceae g Ascidiaceihabitans s Ascidiaceihabitans donghaensis WGS OMOR01
d Bacteria p Proteobacteria c Alphaproteobacteria o Rhodobacterales f Rhodobacteraceae g Sagittula s Sagittula stellata WGS AAYA01
d Bacteria p Proteobacteria c Alphaproteobacteria o Rhodobacterales f Rhodobacteraceae g Marivivens s Marivivens niveibacter WGS MSPP01
d Bacteria p Proteobacteria c Alphaproteobacteria o Rhodobacterales f Rhodobacteraceae g Marivivens s Marivivens niveibacter WGS MSPP01
d Bacteria p Proteobacteria c Alphaproteobacteria o Rhodobacterales f Rhodobacteraceae g Halocynthiibacter s Halocynthiibacter sp003031585 WGS PWAA01
d Bacteria p Proteobacteria c Alphaproteobacteria o Rhodobacterales f Rhodobacteraceae g Cognatishimia s Cognatishimia maritima WGS FQWM01
d Bacteria p Proteobacteria c Alphaproteobacteria o Rhodobacterales f Rhodobacteraceae g R2A62 s R2A62 sp000161835 WGS ACOA01
d Bacteria p Proteobacteria c Alphaproteobacteria o Rhodobacterales f Rhodobacteraceae g Aliiroseovarius s Aliiroseovarius crassostreae WGS LKBA01
d Bacteria p Proteobacteria c Alphaproteobacteria o Rhodobacterales f Rhodobacteraceae g 56-14-T64 s 56-14-T64 sp005771405 WGS VAUA01
d Bacteria p Proteobacteria c Alphaproteobacteria o Rhodobacterales f Rhodobacteraceae g Pseudophaeobacter A s Pseudophaeobacter A gallaeciensis A WGS QEOS01
d Bacteria p Proteobacteria c Alphaproteobacteria o Rhodobacterales f Rhodobacteraceae g Tropicibacter s Tropicibacter naphthalenivorans WGS CYSE01
d Bacteria p Proteobacteria c Alphaproteobacteria o Rhodobacterales f Rhodobacteraceae g Pseudodonghicola s Pseudodonghicola sp004348975 WGS SMFP01
d Bacteria p Proteobacteria c Alphaproteobacteria o Rhodobacterales f Rhodobacteraceae g MYP11 s MYP11 sp004803475 WGS SRKY01
d Bacteria p Proteobacteria c Alphaproteobacteria o Rhodobacterales f Rhodobacteraceae g HTCC2150 s HTCC2150 sp000169395 WGS AAXZ01
d Bacteria p Proteobacteria c Alphaproteobacteria o Rhodobacterales f Rhodobacteraceae g Planktotalea s Planktotalea frisia WGS MLCB01
d Bacteria p Proteobacteria c Alphaproteobacteria o Rhodobacterales f Rhodobacteraceae g UBA3069 s UBA3069 sp002366795 WGS DFBX01
d Bacteria p Proteobacteria c Alphaproteobacteria o Rhodobacterales f Rhodobacteraceae g Pelagimonas s Pelagimonas varians WGS QKMF01
d Bacteria p Proteobacteria c Alphaproteobacteria o Rhodobacterales f Rhodobacteraceae g Primorskyibacter s Primorskyibacter sedentarius WGS SLZU01
d Bacteria p Armatimonadota c Abditibacteria o CG2-30-59-28 f CG2-30-59-28 g CG2-30-59-28 s CG2-30-59-28 sp001872605 WGS PEWH01
d Bacteria p Armatimonadota c Abditibacteria o Abditibacteriales f Abditibacteriaceae g Abditibacterium s Abditibacterium sp004172935 WGS SDZD01
d Bacteria p Lindowbacteria c 2-12-FULL-62-27 o 2-12-FULL-62-27 f 2-12-FULL-62-27 g 2-12-FULL-62-27 s 2-12-FULL-62-27 sp001784175 WGS MFPR01
d Bacteria p Chloroflexota c Ktedonobacteria o Ktedonobacterales f Ktedonobacteraceae g CF-113 s CF-113 sp005881995 WGS VBFM01
d Bacteria p Chloroflexota c Ktedonobacteria o Ktedonobacterales f Ktedonobacteraceae g Thermogemmatispora s Thermogemmatispora onikobensis WGS BDGT01
d Bacteria p Chloroflexota c UBA11872 o UBA11872 f UBA11872 g UBA11872 s UBA11872 sp002433065 WGS DPMY01
d Bacteria p Chloroflexota c Dehalococcoidia o SM23-28-2 f RBG-16-68-14 g RBG-16-68-14 s RBG-16-68-14 sp001796995 WGS MGOB01
d Bacteria p Elusimicrobiota c Elusimicrobia o 2-01-FULL-59-12 f 2-01-FULL-59-12 g 2-01-FULL-59-12 s 2-01-FULL-59-12 sp001800075 WGS MGUR01
d Bacteria p Elusimicrobiota c Elusimicrobia o 2-02-FULL-39-32 f 2-01-FULL-60-11 g 2-01-FULL-64-13 s 2-01-FULL-64-13 sp001800095 WGS MGUT01
d Bacteria p Armatimonadota c HRBIN16 o HRBIN16 f HRBIN16 g HRBIN16 s HRBIN16 sp002898895 WGS BEHS01
d Bacteria p Chloroflexota c Dehalococcoidia o SM23-28-2 f RBG-16-64-32 g CF-163 s CF-163 sp005882985 WGS VBEJ01
d Bacteria p Chloroflexota c UBA6077 o UBA6077 f CF-72 g CF-72 s CF-72 sp005883405 WGS VBGG01
d Bacteria p Eremiobacterota c Eremiobacteria o Eremiobacterales f Eremiobacteraceae g Eremiobacter s Eremiobacter sp003244105 WGS QHBK01
d Bacteria p Eremiobacterota c Eremiobacteria o UBP12 f UBA5184 g Palsa-1483 s Palsa-1483 sp003158175 WGS PMHJ01
d Bacteria p Eremiobacterota c Eremiobacteria o UBP12 f UBA5184 g PALSA-1529 s PALSA-1529 sp003140835 WGS PLLA01
d Bacteria p UBA10199 c UBA10199 o UBA10199 f SBBF01 g SBBF01 s SBBF01 sp005240075 WGS SBBF01
d Bacteria p UBA10199 c UBA10199 o UBA10199 f 2-12-FULL-40-28 g 2-12-FULL-40-28 s 2-12-FULL-40-28 sp001798135 WGS MGRJ01
d Bacteria p Actinobacteriota c Thermoleophilia o Gaiellales f Gaiellaceae g AC-21 s AC-21 sp005883995 WGS VAXB01
d Bacteria p Actinobacteriota c Thermoleophilia o Gaiellales f Gaiellaceae g 3-1-20CM-4-69-9 s 3-1-20CM-4-69-9 sp005883355 WGS VAWX01
d Bacteria p Actinobacteriota c Thermoleophilia o Gaiellales f Gaiellaceae g GMQP-bins7 s GMQP-bins7 sp004366385 WGS SIRU01
d Bacteria p Actinobacteriota c Thermoleophilia o Gaiellales f Gaiellaceae g PALSA-612 s PALSA-612 sp003134505 WGS PLBK01
d Bacteria p Actinobacteriota c Thermoleophilia o Gaiellales f Gaiellaceae g AC-32 s AC-32 sp005883335 WGS VAXM01
d Bacteria p Actinobacteriota c Thermoleophilia o Gaiellales f Gaiellaceae g VAXT01 s VAXT01 sp005883245 WGS VAXT01
d Bacteria p Chloroflexota c Ellin6529 o CSP1-4 f CSP1-4 g GWC2-73-18 s GWC2-73-18 sp001794945 WGS MGMJ01
d Bacteria p Chloroflexota c Ellin6529 o QHBO01 f QHBO01 g CF-167 s CF-167 sp005879775 WGS VBJX01
d Bacteria p Myxococcota c Polyangia o Polyangiales f Polyangiaceae g Palsa-1150 s Palsa-1150 sp003139415 WGS PLIR01
d Archaea p Thermoproteota c Bathyarchaeia o TCS64 f TCS64 g GCA-2726865 s GCA-2726865 sp002726865 WGS PBSW01
d Bacteria p Acidobacteriota c Aminicenantia o Aminicenantales f Aminicenantaceae A g JdFR-80 s JdFR-80 sp003648765 WGS QMPV01
d Bacteria p Calditrichota c Calditrichia o Calditrichales f Calditrichaceae g Caldithrix s Caldithrix abyssi WGS AGCQ01
d Bacteria p Calditrichota c Calditrichia o Calditrichales f Calditrichaceae g Zgenome-0002 s Zgenome-0002 sp003854985 WGS RQOG01
d Bacteria p Firmicutes B c Desulfotomaculia o Desulfotomaculales f Desulfotomaculaceae g Desulfohalotomaculum s Desulfohalotomaculum alkaliphilum WGS JONT01
d Bacteria p Firmicutes B c Desulfotomaculia o Desulfotomaculales f Desulfofarciminaceae g Desulfofarcimen A s Desulfofarcimen A intricatum WGS BCWE01
d Bacteria p Firmicutes B c Desulfotomaculia o Desulfotomaculales f Desulfocucumaceae g BRHc4a s BRHc4a sp000802095 WGS JUEB01
d Bacteria p Firmicutes B c Desulfotomaculia o Desulfotomaculales f Desulfurisporaceae g Desulfurispora s Desulfurispora thermophila WGS AQWN01
d Bacteria p Firmicutes B c Desulfotomaculia o Desulfotomaculales f Desulfocucumaceae g Desulfocucumis s Desulfocucumis sp000961595 WGS LADO01
d Bacteria p Firmicutes B c Desulfotomaculia o Desulfotomaculales f Desulfovirgulaceae g Desulfotomaculum G s Desulfotomaculum G copahuensis WGS LYVF01
d Bacteria p Firmicutes B c Desulfitobacteriia o Desulfitobacteriales f Desulfitobacteriaceae g Desulfosporosinus s Desulfosporosinus lacus WGS FQXJ01
d Bacteria p Desulfobacterota c Dissulfuribacteria o Dissulfuribacterales f UBA5754 g UBA5754 s UBA5754 sp002419025 WGS DIFG01
d Bacteria p Firmicutes B c Desulfotomaculia o Ammonifexales f Desulforudaceae g SURF-60 s SURF-60 sp003604975 WGS QZIR01
d Bacteria p Firmicutes B c Desulfotomaculia o Ammonifexales f Desulforudaceae g Desulfopertinax s Desulfopertinax sp002375985 WGS DFNH01
d Bacteria p Firmicutes B c Desulfotomaculia o Desulfotomaculales f Desulfotomaculaceae A g Desulfallas A s Desulfallas A alcoholivorax WGS AUMW01
d Bacteria p Firmicutes B c Desulfotomaculia o Desulfotomaculales f Desulfotomaculaceae A g Desulfallas B s Desulfallas B geothermicus WGS FOYM01
d Bacteria p Firmicutes B c Desulfotomaculia o Desulfotomaculales f Desulfotomaculaceae A g BRH-c8a s BRH-c8a sp000961575 WGS LADP01
d Bacteria p Desulfobacterota c BSN033 o B13-G15 f RBG-16-54-18 g B11-G15 s B11-G15 sp003646745 WGS QMLM01
d Bacteria p Abyssubacteria c SURF-5 o SURF-5 f SURF-5 g SURF-5 s SURF-5 sp003598085 WGS QZKU01
d Bacteria p Abyssubacteria c SURF-5 o SURF-5 f SURF-5 g SURF-17 s SURF-17 sp003598055 WGS QZKI01
d Bacteria p Actinobacteriota c UBA9087 o UBA10029 f UBA10029 g CG2-30-50-142 s CG2-30-50-142 sp001871795 WGS PFKS01
d Bacteria p Actinobacteriota c UBA9087 o UBA10029 f UBA10029 g UBA10029 s UBA10029 sp001767575 WGS MELI01
d Bacteria p Nitrospinota c UBA9942 o UBA9942 f SZUA-224 g SZUA-224 s SZUA-224 sp003229375 WGS QIDP01
d Bacteria p UBA9089 c CG2-30-40-21 o CG2-30-40-21 f CG2-30-40-21 g CG2-30-40-21 s CG2-30-40-21 sp001873945 WGS PFUI01
d Bacteria p UBA9089 c CG2-30-40-21 o CG2-30-40-21 f SBAY01 g SBAY01 s SBAY01 sp005239945 WGS SBAY01
d Bacteria p Desulfobacterota c Syntrophobacteria o BM002 f BM002 g BM002 s BM002 sp002899795 WGS PPDG01
d Bacteria p Gemmatimonadota A c GCA-2686955 o GCA-2686955 f GCA-2686955 g GCA-2686955 s GCA-2686955 sp002686955 WGS NYZA01
d Bacteria p Acidobacteriota c UBA4820 o UBA4820 f UBA4820 g UBA4820 s UBA4820 sp002402325 WGS DHFQ01
d Bacteria p Acidobacteriota c Thermoanaerobaculia o Thermoanaerobaculales f Thermoanaerobaculaceae g RBG-13-68-16 s RBG-13-68-16 sp004298115 WGS SCRJ01
d Bacteria p Acidobacteriota c Acidobacteriae o Acidobacteriales f Koribacteraceae g Bog-209 s Bog-209 sp003139095 WGS PLHW01
d Bacteria p Acidobacteriota c Thermoanaerobaculia o UBA5704 f UBA5704 g M0029 s M0029 sp006227875 WGS SBFI01
d Bacteria p Acidobacteriota c Acidobacteriae o UBA7540 f UBA7540 g Bog-366 s Bog-366 sp003165615 WGS PLWR01
d Bacteria p Desulfobacterota c Desulfobacteria o Desulfatiglandales f HGW-15 g HGW-15 s HGW-15 sp002840535 WGS PHBH01
d Bacteria p Desulfobacterota c Desulfobacteria o Desulfatiglandales f B17-G16 g B17-G16 s B17-G16 sp003646975 WGS QMLY01
d Bacteria p Desulfobacterota c Syntrophobacteria o Syntrophobacterales f DSM-9756 g Desulfosoma s Desulfosoma caldarium WGS RJVA01
d Bacteria p Desulfobacterota c Syntrophobacteria o Syntrophobacterales f DSM-9756 g Desulfacinum s Desulfacinum hydrothermale WGS FWXF01
d Bacteria p Desulfobacterota c Syntrophobacteria o Syntrophobacterales f Syntrophobacteraceae g SLCH01 s SLCH01 sp003566995 WGS PXBG01
d Bacteria p Desulfobacterota c Syntrophobacteria o Syntrophobacterales f DSM-9756 g CG2-30-61-12 s CG2-30-61-12 sp001873705 WGS MNZR01
d Bacteria p Desulfobacterota c Syntrophobacteria o Syntrophobacterales f Syntrophobacteraceae g Desulforhabdus s Desulforhabdus sp007244385 WGS SCVX01
d Bacteria p SZUA-79 c SZUA-79 o Acidulodesulfobacterales f SZUA-79 g Acidulodesulfobacterium s Acidulodesulfobacterium ferriphilum WGS SGBD01
d Bacteria p SZUA-79 c SZUA-79 o Acidulodesulfobacterales f SZUA-79 g Acididesulfobacter s Acididesulfobacter guangdongensis WGS SGBC01
d Bacteria p Gemmatimonadota c Gemmatimonadetes o SG8-23 f UBA6960 g UBA2988 s UBA2988 sp002346665 WGS DEAG01
d Bacteria p Methylomirabilota c Methylomirabilia o Rokubacteriales f GWA2-73-35 g UBA12499 s UBA12499 sp001788395 WGS MGBT01
d Bacteria p Chloroflexota c Ellin6529 o CSP1-4 f CSP1-4 g CSP1-4 s CSP1-4 sp001443375 WGS LDXM01
d Bacteria p Dormibacterota c Dormibacteria o UBA8260 f UBA8260 g CF-96 s CF-96 sp005889515 WGS VBHE01

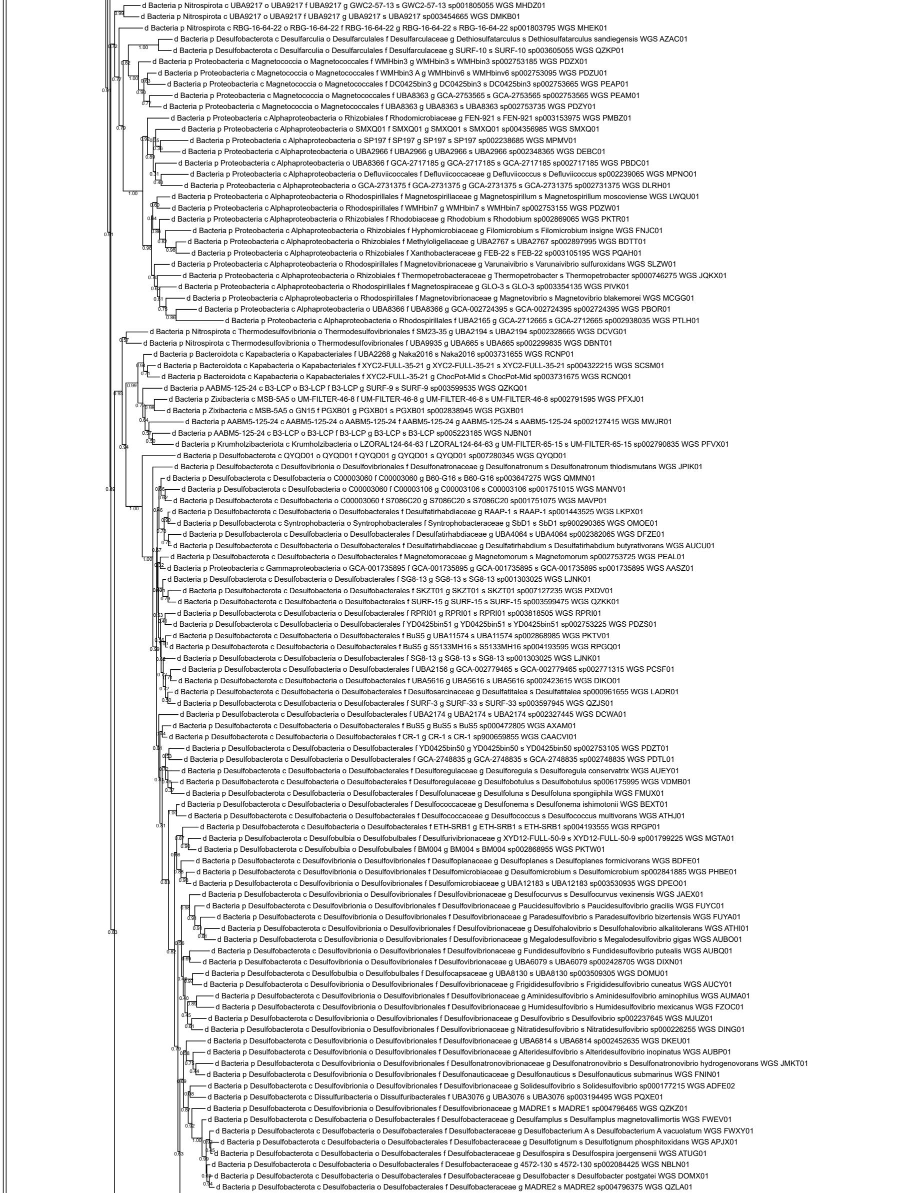
d Bacteria p Nitrospirota c UBA9217 o UBA9217 f UBA9217 g GWC2-57-13 s GWC2-57-13 sp001805055 WGS MHDZ01
d Bacteria p Nitrospirota c UBA9217 o UBA9217 f UBA9217 g UBA9217 s UBA9217 sp003454665 WGS DMKB01
d Bacteria p Nitrospirota c RBG-16-64-22 o RBG-16-64-22 f RBG-16-64-22 g RBG-16-64-22 s RBG-16-64-22 sp001803795 WGS MHEK01
d Bacteria p Desulfobacterota c Desulfarculia o Desulfarculales f Desulfarculaceae g Dethiosulfatarculus s Dethiosulfatarculus sandiegensis WGS AZAC01
d Bacteria p Desulfobacterota c Desulfarculia o Desulfarculales f Desulfarculaceae g SURF-10 s SURF-10 sp003605055 WGS QZKP01
d Bacteria p Proteobacteria c Magnetococcia o Magnetococcales f WMHbin3 g WMHbin3 s WMHbin3 sp002753185 WGS PDZX01
d Bacteria p Proteobacteria c Magnetococcia o Magnetococcales f WMHbin3 A g WMHbinv6 s WMHbinv6 sp002753095 WGS PDZU01
d Bacteria p Proteobacteria c Magnetococcia o Magnetococcales f DC0425bin3 g DC0425bin3 s DC0425bin3 sp002753665 WGS PEAP01
d Bacteria p Proteobacteria c Magnetococcia o Magnetococcales f UBA8363 g GCA-2753565 s GCA-2753565 sp002753565 WGS PEAM01
d Bacteria p Proteobacteria c Magnetococcia o Magnetococcales f UBA8363 g UBA8363 s UBA8363 sp002753735 WGS PDZY01
d Bacteria p Proteobacteria c Alphaproteobacteria o Rhizobiales f Rhodomicrobiaceae g FEN-921 s FEN-921 sp003153975 WGS PMBZ01
d Bacteria p Proteobacteria c Alphaproteobacteria o SMXQ01 f SMXQ01 g SMXQ01 s SMXQ01 sp004356985 WGS SMXQ01
d Bacteria p Proteobacteria c Alphaproteobacteria o SP197 f SP197 g SP197 s SP197 sp002238685 WGS MPMV01
d Bacteria p Proteobacteria c Alphaproteobacteria o UBA2966 f UBA2966 g UBA2966 s UBA2966 sp002348365 WGS DEBC01
d Bacteria p Proteobacteria c Alphaproteobacteria o UBA8366 f GCA-2717185 g GCA-2717185 s GCA-2717185 sp002717185 WGS PBDC01
d Bacteria p Proteobacteria c Alphaproteobacteria o Defluviicoccales f Defluviicoccaceae g Defluviicoccus s Defluviicoccus sp002239065 WGS MPNO01
d Bacteria p Proteobacteria c Alphaproteobacteria o GCA-2731375 f GCA-2731375 g GCA-2731375 s GCA-2731375 sp002731375 WGS DLRH01
d Bacteria p Proteobacteria c Alphaproteobacteria o Rhodospirillales f Magnetospirillaceae g Magnetospirillum s Magnetospirillum moscoviense WGS LWQU01
d Bacteria p Proteobacteria c Alphaproteobacteria o Rhodospirillales f WMHbin7 g WMHbin7 s WMHbin7 sp002753155 WGS PDZW01
d Bacteria p Proteobacteria c Alphaproteobacteria o Rhizobiales f Rhodobiaceae g Rhodobium s Rhodobium sp002869065 WGS PKTR01
d Bacteria p Proteobacteria c Alphaproteobacteria o Rhizobiales f Hyphomicrobiaceae g Filomicrobium s Filomicrobium insigne WGS FNJC01
d Bacteria p Proteobacteria c Alphaproteobacteria o Rhizobiales f Methyloligellaceae g UBA2767 s UBA2767 sp002897995 WGS BDTT01
d Bacteria p Proteobacteria c Alphaproteobacteria o Rhizobiales f Xanthobacteraceae g FEB-22 s FEB-22 sp003105195 WGS PQAH01
d Bacteria p Proteobacteria c Alphaproteobacteria o Rhodospirillales f Magnetovibrionaceae g Varunaivibrio s Varunaivibrio sulfuroxidans WGS SLZW01
d Bacteria p Proteobacteria c Alphaproteobacteria o Rhizobiales f Thermopetrobacteraceae g Thermopetrobacter s Thermopetrobacter sp000746275 WGS JQKX01
d Bacteria p Proteobacteria c Alphaproteobacteria o Rhodospirillales f Magnetospiraceae g GLO-3 s GLO-3 sp003354135 WGS PIVK01
d Bacteria p Proteobacteria c Alphaproteobacteria o Rhodospirillales f Magnetovibrionaceae g Magnetovibrio s Magnetovibrio blakemorei WGS MCGG01
d Bacteria p Proteobacteria c Alphaproteobacteria o UBA8366 f UBA8366 g GCA-002724395 s GCA-002724395 sp002724395 WGS PBOR01
d Bacteria p Proteobacteria c Alphaproteobacteria o Rhodospirillales f UBA2165 g GCA-2712665 s GCA-2712665 sp002938035 WGS PTLH01
d Bacteria p Nitrospirota c Thermodesulfovibrionia o Thermodesulfovibrionales f SM23-35 g UBA2194 s UBA2194 sp002328665 WGS DCVG01
d Bacteria p Nitrospirota c Thermodesulfovibrionia o Thermodesulfovibrionales f UBA9935 g UBA665 s UBA665 sp002299835 WGS DBNT01
d Bacteria p Bacteroidota c Kapabacteria o Kapabacteriales f UBA2268 g Naka2016 s Naka2016 sp003731655 WGS RCNP01
d Bacteria p Bacteroidota c Kapabacteria o Kapabacteriales f XYC2-FULL-35-21 g XYC2-FULL-35-21 s XYC2-FULL-35-21 sp004322215 WGS SCSM01
d Bacteria p Bacteroidota c Kapabacteria o Kapabacteriales f XYC2-FULL-35-21 g ChocPot-Mid s ChocPot-Mid sp003731675 WGS RCNQ01
d Bacteria p AABM5-125-24 c B3-LCP o B3-LCP f B3-LCP g SURF-9 s SURF-9 sp003599535 WGS QZKQ01
d Bacteria p Zixibacteria c MSB-5A5 o UM-FILTER-46-8 f UM-FILTER-46-8 g UM-FILTER-46-8 s UM-FILTER-46-8 sp002791595 WGS PFXJ01
d Bacteria p Zixibacteria c MSB-5A5 o GN15 f PGXB01 g PGXB01 s PGXB01 sp002838945 WGS PGXB01
d Bacteria p AABM5-125-24 c AABM5-125-24 o AABM5-125-24 f AABM5-125-24 g AABM5-125-24 s AABM5-125-24 sp002127415 WGS MWJR01
d Bacteria p AABM5-125-24 c B3-LCP o B3-LCP f B3-LCP g B3-LCP s B3-LCP sp005223185 WGS NJBN01
d Bacteria p Krumholzibacteriota c Krumholzibacteria o LZORAL124-64-63 f LZORAL124-64-63 g UM-FILTER-65-15 s UM-FILTER-65-15 sp002790835 WGS PFVX01
d Bacteria p Desulfobacterota c QYQD01 o QYQD01 f QYQD01 g QYQD01 s QYQD01 sp007280345 WGS QYQD01
d Bacteria p Desulfobacterota c Desulfovibrionia o Desulfovibrionales f Desulfonatronaceae g Desulfonatronum s Desulfonatronum thiodismutans WGS JPIK01
d Bacteria p Desulfobacterota c Desulfobacteria o C00003060 f C00003060 g B60-G16 s B60-G16 sp003647275 WGS QMMN01
d Bacteria p Desulfobacterota c Desulfobacteria o C00003060 f C00003106 g C00003106 s C00003106 sp001751015 WGS MANV01
d Bacteria p Desulfobacterota c Desulfobacteria o C00003060 f S7086C20 g S7086C20 s S7086C20 sp001751075 WGS MAVP01
d Bacteria p Desulfobacterota c Desulfobacteria o Desulfobacterales f Desulfatirhabdiaceae g RAAP-1 s RAAP-1 sp001443525 WGS LKPX01
d Bacteria p Desulfobacterota c Syntrophobacteria o Syntrophobacterales f Syntrophobacteraceae g SbD1 s SbD1 sp900290365 WGS OMOE01
d Bacteria p Desulfobacterota c Desulfobacteria o Desulfobacterales f Desulfatirhabdiaceae g UBA4064 s UBA4064 sp002382065 WGS DFZE01
d Bacteria p Desulfobacterota c Desulfobacteria o Desulfobacterales f Desulfatirhabdiaceae g Desulfatirhabdium s Desulfatirhabdium butyrativorans WGS AUCU01
d Bacteria p Desulfobacterota c Desulfobacteria o Desulfobacterales f Magnetomoraceae g Magnetomorum s Magnetomorum sp002753725 WGS PEAL01
d Bacteria p Proteobacteria c Gammaproteobacteria o GCA-001735895 f GCA-001735895 g GCA-001735895 s GCA-001735895 sp001735895 WGS AASZ01
d Bacteria p Desulfobacterota c Desulfobacteria o Desulfobacterales f SG8-13 g SG8-13 s SG8-13 sp001303025 WGS LJNK01
d Bacteria p Desulfobacterota c Desulfobacteria o Desulfobacterales f SKZT01 g SKZT01 s SKZT01 sp007127235 WGS PXDV01
d Bacteria p Desulfobacterota c Desulfobacteria o Desulfobacterales f SURF-15 g SURF-15 s SURF-15 sp003599475 WGS QZKK01
d Bacteria p Desulfobacterota c Desulfobacteria o Desulfobacterales f RPRI01 g RPRI01 s RPRI01 sp003818505 WGS RPRI01
d Bacteria p Desulfobacterota c Desulfobacteria o Desulfobacterales f YD0425bin51 g YD0425bin51 s YD0425bin51 sp002753225 WGS PDZS01
d Bacteria p Desulfobacterota c Desulfobacteria o Desulfobacterales f BuS5 g UBA11574 s UBA11574 sp002868985 WGS PKTV01
d Bacteria p Desulfobacterota c Desulfobacteria o Desulfobacterales f BuS5 g S5133MH16 s S5133MH16 sp004193595 WGS RPGQ01
d Bacteria p Desulfobacterota c Desulfobacteria o Desulfobacterales f SG8-13 g SG8-13 s SG8-13 sp001303025 WGS LJNK01
d Bacteria p Desulfobacterota c Desulfobacteria o Desulfobacterales f UBA2156 g GCA-002779465 s GCA-002779465 sp002771315 WGS PCSF01
d Bacteria p Desulfobacterota c Desulfobacteria o Desulfobacterales f UBA5616 g UBA5616 s UBA5616 sp002423615 WGS DIKO01
d Bacteria p Desulfobacterota c Desulfobacteria o Desulfobacterales f Desulfosarcinaceae g Desulfatitalea s Desulfatitalea sp000961655 WGS LADR01
d Bacteria p Desulfobacterota c Desulfobacteria o Desulfobacterales f SURF-3 g SURF-33 s SURF-33 sp003597945 WGS QZJS01
d Bacteria p Desulfobacterota c Desulfobacteria o Desulfobacterales f UBA2174 g UBA2174 s UBA2174 sp002327445 WGS DCWA01
d Bacteria p Desulfobacterota c Desulfobacteria o Desulfobacterales f BuS5 g BuS5 s BuS5 sp000472805 WGS AXAM01
d Bacteria p Desulfobacterota c Desulfobacteria o Desulfobacterales f CR-1 g CR-1 s CR-1 sp900659855 WGS CAACVI01
d Bacteria p Desulfobacterota c Desulfobacteria o Desulfobacterales f YD0425bin50 g YD0425bin50 s YD0425bin50 sp002753105 WGS PDZT01
d Bacteria p Desulfobacterota c Desulfobacteria o Desulfobacterales f GCA-2748835 g GCA-2748835 s GCA-2748835 sp002748835 WGS PDTL01
d Bacteria p Desulfobacterota c Desulfobacteria o Desulfobacterales f Desulforegulaceae g Desulforegula s Desulforegula conservatrix WGS AUEY01
d Bacteria p Desulfobacterota c Desulfobacteria o Desulfobacterales f Desulforegulaceae g Desulfobotulus s Desulfobotulus sp006175995 WGS VDMB01
d Bacteria p Desulfobacterota c Desulfobacteria o Desulfobacterales f Desulfolunaceae g Desulfoluna s Desulfoluna spongiiphila WGS FMUX01
d Bacteria p Desulfobacterota c Desulfobacteria o Desulfobacterales f Desulfococcaceae g Desulfonema s Desulfonema ishimotonii WGS BEXT01
d Bacteria p Desulfobacterota c Desulfobacteria o Desulfobacterales f Desulfococcaceae g Desulfococcus s Desulfococcus multivorans WGS ATHJ01
d Bacteria p Desulfobacterota c Desulfobacteria o Desulfobacterales f ETH-SRB1 g ETH-SRB1 s ETH-SRB1 sp004193555 WGS RPGP01
d Bacteria p Desulfobacterota c Desulfobulbia o Desulfobulbales f Desulfurivibrionaceae g XYD12-FULL-50-9 s XYD12-FULL-50-9 sp001799225 WGS MGTA01
d Bacteria p Desulfobacterota c Desulfobulbia o Desulfobulbales f BM004 g BM004 s BM004 sp002868955 WGS PKTW01
d Bacteria p Desulfobacterota c Desulfovibrionia o Desulfovibrionales f Desulfoplanaceae g Desulfoplanes s Desulfoplanes formicivorans WGS BDFE01
d Bacteria p Desulfobacterota c Desulfovibrionia o Desulfovibrionales f Desulfomicrobiaceae g Desulfomicrobium s Desulfomicrobium sp002841885 WGS PHBE01
d Bacteria p Desulfobacterota c Desulfovibrionia o Desulfovibrionales f Desulfomicrobiaceae g UBA12183 s UBA12183 sp003530935 WGS DPEO01
d Bacteria p Desulfobacterota c Desulfovibrionia o Desulfovibrionales f Desulfovibrionaceae g Desulfocurvus s Desulfocurvus vexinensis WGS JAEX01
d Bacteria p Desulfobacterota c Desulfovibrionia o Desulfovibrionales f Desulfovibrionaceae g Paucidesulfovibrio s Paucidesulfovibrio gracilis WGS FUYC01
d Bacteria p Desulfobacterota c Desulfovibrionia o Desulfovibrionales f Desulfovibrionaceae g Paradesulfovibrio s Paradesulfovibrio bizertensis WGS FUYA01
d Bacteria p Desulfobacterota c Desulfovibrionia o Desulfovibrionales f Desulfovibrionaceae g Desulfohalovibrio s Desulfohalovibrio alkalitolerans WGS ATHI01
d Bacteria p Desulfobacterota c Desulfovibrionia o Desulfovibrionales f Desulfovibrionaceae g Megalodesulfovibrio s Megalodesulfovibrio gigas WGS AUBO01
d Bacteria p Desulfobacterota c Desulfovibrionia o Desulfovibrionales f Desulfovibrionaceae g Fundidesulfovibrio s Fundidesulfovibrio putealis WGS AUBQ01
d Bacteria p Desulfobacterota c Desulfovibrionia o Desulfovibrionales f Desulfovibrionaceae g UBA6079 s UBA6079 sp002428705 WGS DIXN01
d Bacteria p Desulfobacterota c Desulfobulbia o Desulfobulbales f Desulfocapsaceae g UBA8130 s UBA8130 sp003509305 WGS DOMU01
d Bacteria p Desulfobacterota c Desulfovibrionia o Desulfovibrionales f Desulfovibrionaceae g Frigidesulfovibrio s Frigidesulfovibrio cuneatus WGS AUCY01
d Bacteria p Desulfobacterota c Desulfovibrionia o Desulfovibrionales f Desulfovibrionaceae g Aminidesulfovibrio s Aminidesulfovibrio aminophilus WGS AUMA01
d Bacteria p Desulfobacterota c Desulfovibrionia o Desulfovibrionales f Desulfovibrionaceae g Humidesulfovibrio s Humidesulfovibrio mexicanus WGS FZOC01
d Bacteria p Desulfobacterota c Desulfovibrionia o Desulfovibrionales f Desulfovibrionaceae g Desulfovibrio s Desulfovibrio sp002237645 WGS MJUZ01
d Bacteria p Desulfobacterota c Desulfovibrionia o Desulfovibrionales f Desulfovibrionaceae g Nitratidesulfovibrio s Nitratidesulfovibrio sp000226255 WGS DING01
d Bacteria p Desulfobacterota c Desulfovibrionia o Desulfovibrionales f Desulfovibrionaceae g UBA6814 s UBA6814 sp002452635 WGS DKEU01
d Bacteria p Desulfobacterota c Desulfovibrionia o Desulfovibrionales f Desulfovibrionaceae g Alteridesulfovibrio s Alteridesulfovibrio inopinatus WGS AUBP01
d Bacteria p Desulfobacterota c Desulfovibrionia o Desulfovibrionales f Desulfonatronovibrionaceae g Desulfonatronovibrio s Desulfonatronovibrio hydrogenovorans WGS JMKT01
d Bacteria p Desulfobacterota c Desulfovibrionia o Desulfovibrionales f Desulfonauticaceae g Desulfonauticus s Desulfonauticus submarinus WGS FNIN01
d Bacteria p Desulfobacterota c Desulfovibrionia o Desulfovibrionales f Desulfovibrionaceae g Solidesulfovibrio s Solidesulfovibrio sp000177215 WGS ADFE02
d Bacteria p Desulfobacterota c Dissulfuribacteria o Dissulfuribacterales f UBA3076 g UBA3076 s UBA3076 sp003194495 WGS PQXE01
d Bacteria p Desulfobacterota c Desulfovibrionia o Desulfovibrionales f Desulfovibrionaceae g MADRE1 s MADRE1 sp004796465 WGS QZKZ01
d Bacteria p Desulfobacterota c Desulfobacteria o Desulfobacterales f Desulfamplus g Desulfamplus s Desulfamplus magnetovallimortis WGS FWEV01
d Bacteria p Desulfobacterota c Desulfobacteria o Desulfobacterales f Desulfobacteraceae g Desulfobacterium A s Desulfobacterium A vacuolatum WGS FWXY01
d Bacteria p Desulfobacterota c Desulfobacteria o Desulfobacterales f Desulfobacteraceae g Desulfotignum s Desulfotignum phosphitoxidans WGS APJX01
d Bacteria p Desulfobacterota c Desulfobacteria o Desulfobacterales f Desulfobacteraceae g Desulfospira s Desulfospira joergensenii WGS ATUG01
d Bacteria p Desulfobacterota c Desulfobacteria o Desulfobacterales f Desulfobacteraceae g 4572-130 s 4572-130 sp002084425 WGS NBLN01
d Bacteria p Desulfobacterota c Desulfobacteria o Desulfobacterales f Desulfobacteraceae g Desulfobacter s Desulfobacter postgatei WGS DOMX01
d Bacteria p Desulfobacterota c Desulfobacteria o Desulfobacterales f Desulfobacteraceae g MADRE2 s MADRE2 sp004796375 WGS QZLA01

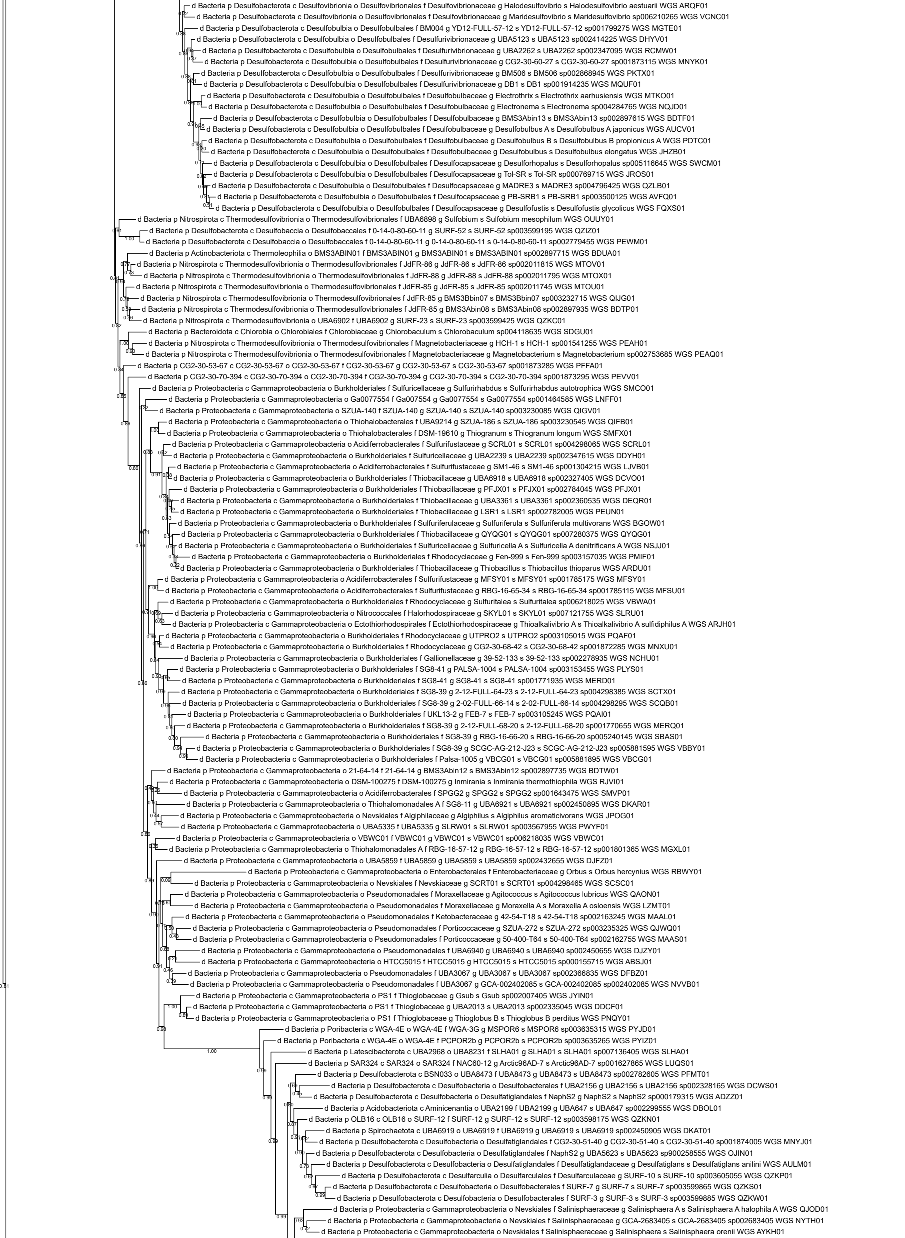
d Bacteria p Desulfobacterota c Desulfovibrionia o Desulfovibrionales f Desulfovibrionaceae g Halodesulfovibrio s Halodesulfovibrio aestuarii WGS ARQF01
d Bacteria p Desulfobacterota c Desulfovibrionia o Desulfovibrionales f Desulfovibrionaceae g Maridesulfovibrio s Maridesulfovibrio sp006210265 WGS VCNC01
d Bacteria p Desulfobacterota c Desulfobulbia o Desulfobulbales f BM004 g YD12-FULL-57-12 s YD12-FULL-57-12 sp001799275 WGS MGTE01
d Bacteria p Desulfobacterota c Desulfobulbia o Desulfobulbales f Desulfurivibrionaceae g UBA5123 s UBA5123 sp002414225 WGS DHYV01
d Bacteria p Desulfobacterota c Desulfobulbia o Desulfobulbales f Desulfurivibrionaceae g UBA2262 s UBA2262 sp002347095 WGS RCMW01
d Bacteria p Desulfobacterota c Desulfobulbia o Desulfobulbales f Desulfurivibrionaceae g CG2-30-60-27 s CG2-30-60-27 sp001873115 WGS MNYK01
d Bacteria p Desulfobacterota c Desulfobulbia o Desulfobulbales f Desulfurivibrionaceae g BM506 s BM506 sp002868945 WGS PKTX01
d Bacteria p Desulfobacterota c Desulfobulbia o Desulfobulbales f Desulfurivibrionaceae g DB1 s DB1 sp001914235 WGS MQUF01
d Bacteria p Desulfobacterota c Desulfobulbia o Desulfobulbales f Desulfobulbaceae g Electrothrix s Electrothrix aarhusiensis WGS MTKO01
d Bacteria p Desulfobacterota c Desulfobulbia o Desulfobulbales f Desulfobulbaceae g Electronema s Electronema sp004284765 WGS NQJD01
d Bacteria p Desulfobacterota c Desulfobulbia o Desulfobulbales f Desulfobulbaceae g BMS3Abin13 s BMS3Abin13 sp002897615 WGS BDTF01
d Bacteria p Desulfobacterota c Desulfobulbia o Desulfobulbales f Desulfobulbaceae g Desulfobulbus A s Desulfobulbus A japonicus WGS AUCV01
d Bacteria p Desulfobacterota c Desulfobulbia o Desulfobulbales f Desulfobulbaceae g Desulfobulbus B s Desulfobulbus B propionicus A WGS PDTC01
d Bacteria p Desulfobacterota c Desulfobulbia o Desulfobulbales f Desulfobulbaceae g Desulfobulbus s Desulfobulbus elongatus WGS JHZB01
d Bacteria p Desulfobacterota c Desulfobulbia o Desulfobulbales f Desulfocapsaceae g Desulforhopalus s Desulforhopalus sp005116645 WGS SWCM01
d Bacteria p Desulfobacterota c Desulfobulbia o Desulfobulbales f Desulfocapsaceae g Tol-SR s Tol-SR sp000769715 WGS JROS01
d Bacteria p Desulfobacterota c Desulfobulbia o Desulfobulbales f Desulfocapsaceae g MADRE3 s MADRE3 sp004796425 WGS QZLB01
d Bacteria p Desulfobacterota c Desulfobulbia o Desulfobulbales f Desulfocapsaceae g PB-SRB1 s PB-SRB1 sp003500125 WGS AVFQ01
d Bacteria p Desulfobacterota c Desulfobulbia o Desulfobulbales f Desulfocapsaceae g Desulfofustis s Desulfofustis glycolicus WGS FQXS01
d Bacteria p Nitrospirota c Thermodesulfovibrionia o Thermodesulfovibrionales f UBA6898 g Sulfobium s Sulfobium mesophilum WGS OUUY01
d Bacteria p Desulfobacterota c Desulfobaccia o Desulfobaccales f 0-14-0-80-60-11 g SURF-52 s SURF-52 sp003599195 WGS QZIZ01
d Bacteria p Desulfobacterota c Desulfobaccia o Desulfobaccales f 0-14-0-80-60-11 g 0-14-0-80-60-11 s 0-14-0-80-60-11 sp002779455 WGS PEWM01
d Bacteria p Actinobacteriota c Thermoleophilia o BMS3ABIN01 f BMS3ABIN01 g BMS3ABIN01 s BMS3ABIN01 sp002897715 WGS BDUA01
d Bacteria p Nitrospirota c Thermodesulfovibrionia o Thermodesulfovibrionales f JdFR-86 g JdFR-86 s JdFR-86 sp002011815 WGS MTOV01
d Bacteria p Nitrospirota c Thermodesulfovibrionia o Thermodesulfovibrionales f JdFR-88 g JdFR-88 s JdFR-88 sp002011795 WGS MTOX01
d Bacteria p Nitrospirota c Thermodesulfovibrionia o Thermodesulfovibrionales f JdFR-85 g JdFR-85 s JdFR-85 sp002011745 WGS MTOU01
d Bacteria p Nitrospirota c Thermodesulfovibrionia o Thermodesulfovibrionales f JdFR-85 g BMS3Bbin07 s BMS3Bbin07 sp003232715 WGS QIJG01
d Bacteria p Nitrospirota c Thermodesulfovibrionia o Thermodesulfovibrionales f JdFR-85 g BMS3Abin08 s BMS3Abin08 sp002897935 WGS BDTP01
d Bacteria p Nitrospirota c Thermodesulfovibrionia o UBA6902 f UBA6902 g SURF-23 s SURF-23 sp003599425 WGS QZKC01
d Bacteria p Bacteroidota c Chlorobia o Chlorobiales f Chlorobiaceae g Chlorobaculum s Chlorobaculum sp004118635 WGS SDGU01
d Bacteria p Nitrospirota c Thermodesulfovibrionia o Thermodesulfovibrionales f Magnetobacteriaceae g HCH-1 s HCH-1 sp001541255 WGS PEAH01
d Bacteria p Nitrospirota c Thermodesulfovibrionia o Thermodesulfovibrionales f Magnetobacteriaceae g Magnetobacterium s Magnetobacterium sp002753685 WGS PEAQ01
d Bacteria p CG2-30-53-67 c CG2-30-53-67 o CG2-30-53-67 f CG2-30-53-67 g CG2-30-53-67 s CG2-30-53-67 sp001873285 WGS PFFA01
d Bacteria p CG2-30-70-394 c CG2-30-70-394 o CG2-30-70-394 f CG2-30-70-394 g CG2-30-70-394 s CG2-30-70-394 sp001873295 WGS PEVV01
d Bacteria p Proteobacteria c Gammaproteobacteria o Burkholderiales f Sulfuricellaceae g Sulfurirhabdus s Sulfurirhabdus autotrophica WGS SMCO01
d Bacteria p Proteobacteria c Gammaproteobacteria o Ga0077554 f Ga007554 g Ga0077554 s Ga0077554 sp001464585 WGS LNFF01
d Bacteria p Proteobacteria c Gammaproteobacteria o SZUA-140 f SZUA-140 g SZUA-140 s SZUA-140 sp003230085 WGS QIGV01
d Bacteria p Proteobacteria c Gammaproteobacteria o Thiohalobacterales f UBA9214 g SZUA-186 s SZUA-186 sp003230545 WGS QIFB01
d Bacteria p Proteobacteria c Gammaproteobacteria o Thiohalobacterales f DSM-19610 g Thiogranum s Thiogranum longum WGS SMFX01
d Bacteria p Proteobacteria c Gammaproteobacteria o Acidiferrobacterales f Sulfurifustaceae g SCRL01 s SCRL01 sp004298065 WGS SCRL01
d Bacteria p Proteobacteria c Gammaproteobacteria o Burkholderiales f Sulfuricellaceae g UBA2239 s UBA2239 sp002347615 WGS DDYH01
d Bacteria p Proteobacteria c Gammaproteobacteria o Acidiferrobacterales f Sulfurifustaceae g SM1-46 s SM1-46 sp001304215 WGS LJVB01
d Bacteria p Proteobacteria c Gammaproteobacteria o Burkholderiales f Thiobacillaceae g UBA6918 s UBA6918 sp002327405 WGS DCVO01
d Bacteria p Proteobacteria c Gammaproteobacteria o Burkholderiales f Thiobacillaceae g PFJX01 s PFJX01 sp002784045 WGS PFJX01
d Bacteria p Proteobacteria c Gammaproteobacteria o Burkholderiales f Thiobacillaceae g UBA3361 s UBA3361 sp002360535 WGS DEQR01
d Bacteria p Proteobacteria c Gammaproteobacteria o Burkholderiales f Thiobacillaceae g LSR1 s LSR1 sp002782005 WGS PEUN01
d Bacteria p Proteobacteria c Gammaproteobacteria o Burkholderiales f Sulfuriferulaceae g Sulfuriferula s Sulfuriferula multivorans WGS BGOW01
d Bacteria p Proteobacteria c Gammaproteobacteria o Burkholderiales f Thiobacillaceae g QYQG01 s QYQG01 sp007280375 WGS QYQG01
d Bacteria p Proteobacteria c Gammaproteobacteria o Burkholderiales f Sulfuricellaceae g Sulfuricella A s Sulfuricella A denitrificans A WGS NSJJ01
d Bacteria p Proteobacteria c Gammaproteobacteria o Burkholderiales f Rhodocyclaceae g Fen-999 s Fen-999 sp003157035 WGS PMIF01
d Bacteria p Proteobacteria c Gammaproteobacteria o Burkholderiales f Thiobacillaceae g Thiobacillus s Thiobacillus thioparus WGS ARDU01
d Bacteria p Proteobacteria c Gammaproteobacteria o Acidiferrobacterales f Sulfurifustaceae g MFSY01 s MFSY01 sp001785175 WGS MFSY01
d Bacteria p Proteobacteria c Gammaproteobacteria o Acidiferrobacterales f Sulfurifustaceae g RBG-16-65-34 s RBG-16-65-34 sp001785115 WGS MFSU01
d Bacteria p Proteobacteria c Gammaproteobacteria o Burkholderiales f Rhodocyclaceae g Sulfuritalea s Sulfuritalea sp006218025 WGS VBWA01
d Bacteria p Proteobacteria c Gammaproteobacteria o Nitrococcales f Halorhodospiraceae g SKYL01 s SKYL01 sp007121755 WGS SLRU01
d Bacteria p Proteobacteria c Gammaproteobacteria o Ectothiorhodospirales f Ectothiorhodospiraceae g Thioalkalivibrio A s Thioalkalivibrio A sulfidiphilus A WGS ARJH01
d Bacteria p Proteobacteria c Gammaproteobacteria o Burkholderiales f Rhodocyclaceae g UTPRO2 s UTPRO2 sp003105015 WGS PQAF01
d Bacteria p Proteobacteria c Gammaproteobacteria o Burkholderiales f Rhodocyclaceae g CG2-30-68-42 s CG2-30-68-42 sp001872285 WGS MNXU01
d Bacteria p Proteobacteria c Gammaproteobacteria o Burkholderiales f Gallionellaceae g 39-52-133 s 39-52-133 sp002278935 WGS NCHU01
d Bacteria p Proteobacteria c Gammaproteobacteria o Burkholderiales f SG8-41 g PALSA-1004 s PALSA-1004 sp003153455 WGS PLYS01
d Bacteria p Proteobacteria c Gammaproteobacteria o Burkholderiales f SG8-41 g SG8-41 s SG8-41 sp001771935 WGS MERD01
d Bacteria p Proteobacteria c Gammaproteobacteria o Burkholderiales f SG8-39 g 2-12-FULL-64-23 s 2-12-FULL-64-23 sp004298385 WGS SCTX01
d Bacteria p Proteobacteria c Gammaproteobacteria o Burkholderiales f SG8-39 g 2-02-FULL-66-14 s 2-02-FULL-66-14 sp004298295 WGS SCQB01
d Bacteria p Proteobacteria c Gammaproteobacteria o Burkholderiales f UKL13-2 g FEB-7 s FEB-7 sp003105245 WGS PQAI01
d Bacteria p Proteobacteria c Gammaproteobacteria o Burkholderiales f SG8-39 g 2-12-FULL-68-20 s 2-12-FULL-68-20 sp001770655 WGS MERQ01
d Bacteria p Proteobacteria c Gammaproteobacteria o Burkholderiales f SG8-39 g RBG-16-66-20 s RBG-16-66-20 sp005240145 WGS SBAS01
d Bacteria p Proteobacteria c Gammaproteobacteria o Burkholderiales f SG8-39 g SCGC-AG-212-J23 s SCGC-AG-212-J23 sp005881595 WGS VBBY01
d Bacteria p Proteobacteria c Gammaproteobacteria o Burkholderiales f Palsa-1005 g VBCG01 s VBCG01 sp005881895 WGS VBCG01
d Bacteria p Proteobacteria c Gammaproteobacteria o 21-64-14 f 21-64-14 g BMS3Abin12 s BMS3Abin12 sp002897735 WGS BDTW01
d Bacteria p Proteobacteria c Gammaproteobacteria o DSM-100275 f DSM-100275 g Inmirania s Inmirania thermothiophila WGS RJVI01
d Bacteria p Proteobacteria c Gammaproteobacteria o Acidiferrobacterales f SPGG2 g SPGG2 s SPGG2 sp001643475 WGS SMVP01
d Bacteria p Proteobacteria c Gammaproteobacteria o Thiohalomonadales A f SG8-11 g UBA6921 s UBA6921 sp002450895 WGS DKAR01
d Bacteria p Proteobacteria c Gammaproteobacteria o Nevskiales f Algiphilaceae g Algiphilus s Algiphilus aromaticivorans WGS JPOG01
d Bacteria p Proteobacteria c Gammaproteobacteria o UBA5335 f UBA5335 g SLRW01 s SLRW01 sp003567955 WGS PWYF01
d Bacteria p Proteobacteria c Gammaproteobacteria o VBWC01 f VBWC01 g VBWC01 s VBWC01 sp006218035 WGS VBWC01
d Bacteria p Proteobacteria c Gammaproteobacteria o Thiohalomonadales A f RBG-16-57-12 g RBG-16-57-12 s RBG-16-57-12 sp001801365 WGS MGXL01
d Bacteria p Proteobacteria c Gammaproteobacteria o UBA5859 f UBA5859 g UBA5859 s UBA5859 sp002432655 WGS DJFZ01
d Bacteria p Proteobacteria c Gammaproteobacteria o Enterobacterales f Enterobacteriaceae g Orbus s Orbus hercynius WGS RBWY01
d Bacteria p Proteobacteria c Gammaproteobacteria o Nevskiales f Nevskiaceae g SCRT01 s SCRT01 sp004298465 WGS SCSC01
d Bacteria p Proteobacteria c Gammaproteobacteria o Pseudomonadales f Moraxellaceae g Agitococcus s Agitococcus lubricus WGS QAON01
d Bacteria p Proteobacteria c Gammaproteobacteria o Pseudomonadales f Moraxellaceae g Moraxella A s Moraxella A osloensis WGS LZMT01
d Bacteria p Proteobacteria c Gammaproteobacteria o Pseudomonadales f Ketobacteraceae g 42-54-T18 s 42-54-T18 sp002163245 WGS MAAL01
d Bacteria p Proteobacteria c Gammaproteobacteria o Pseudomonadales f Porticoccaceae g SZUA-272 s SZUA-272 sp003235325 WGS QJWQ01
d Bacteria p Proteobacteria c Gammaproteobacteria o Pseudomonadales f Porticoccaceae g 50-400-T64 s 50-400-T64 sp002162755 WGS MAAS01
d Bacteria p Proteobacteria c Gammaproteobacteria o Pseudomonadales f UBA6940 g UBA6940 s UBA6940 sp002450655 WGS DJZY01
d Bacteria p Proteobacteria c Gammaproteobacteria o HTCC5015 f HTCC5015 g HTCC5015 s HTCC5015 sp000155715 WGS ABSJ01
d Bacteria p Proteobacteria c Gammaproteobacteria o Pseudomonadales f UBA3067 g UBA3067 s UBA3067 sp002366835 WGS DFBZ01
d Bacteria p Proteobacteria c Gammaproteobacteria o Pseudomonadales f UBA3067 g GCA-002402085 s GCA-002402085 sp002402085 WGS NVVB01
d Bacteria p Proteobacteria c Gammaproteobacteria o PS1 f Thioglobaceae g Gsub s Gsub sp002007405 WGS JYIN01
d Bacteria p Proteobacteria c Gammaproteobacteria o PS1 f Thioglobaceae g UBA2013 s UBA2013 sp002335045 WGS DDCF01
d Bacteria p Proteobacteria c Gammaproteobacteria o PS1 f Thioglobaceae g Thioglobus B s Thioglobus B perditus WGS PNQY01
d Bacteria p Poribacteria c WGA-4E o WGA-4E f WGA-3G g MSPOR6 s MSPOR6 sp003635315 WGS PYJD01
d Bacteria p Poribacteria c WGA-4E o WGA-4E f PCPOR2b g PCPOR2b s PCPOR2b sp003635265 WGS PYIZ01
d Bacteria p Latescibacterota c UBA2968 o UBA8231 f SLHA01 g SLHA01 s SLHA01 sp007136405 WGS SLHA01
d Bacteria p SAR324 c SAR324 o SAR324 f NAC60-12 g Arctic96AD-7 s Arctic96AD-7 sp001627865 WGS LUQS01
d Bacteria p Desulfobacterota c BSN033 o UBA8473 f UBA8473 g UBA8473 s UBA8473 sp002782605 WGS PFMT01
d Bacteria p Desulfobacterota c Desulfobacteria o Desulfobacterales f UBA2156 g UBA2156 s UBA2156 sp002328165 WGS DCWS01
d Bacteria p Desulfobacterota c Desulfobacteria o Desulfatiglandales f NaphS2 g NaphS2 s NaphS2 sp000179315 WGS ADZZ01
d Bacteria p Acidobacteriota c Aminicenantia o UBA2199 f UBA2199 g UBA647 s UBA647 sp002299555 WGS DBOL01
d Bacteria p OLB16 c OLB16 o SURF-12 f SURF-12 g SURF-12 s SURF-12 sp003598175 WGS QZKN01
d Bacteria p Spirochaetota c UBA6919 o UBA6919 f UBA6919 g UBA6919 s UBA6919 sp002450905 WGS DKAT01
d Bacteria p Desulfobacterota c Desulfobacteria o Desulfatiglandales f CG2-30-51-40 g CG2-30-51-40 s CG2-30-51-40 sp001874005 WGS MNYJ01
d Bacteria p Desulfobacterota c Desulfobacteria o Desulfatiglandales f NaphS2 g UBA5623 s UBA5623 sp900258555 WGS OJIN01
d Bacteria p Desulfobacterota c Desulfobacteria o Desulfatiglandales f Desulfatiglandaceae g Desulfatiglans s Desulfatiglans anilini WGS AULM01
d Bacteria p Desulfobacterota c Desulfarculia o Desulfarculales f Desulfarculaceae g SURF-10 s SURF-10 sp003605055 WGS QZKP01
d Bacteria p Desulfobacterota c Desulfobacteria o Desulfobacterales f SURF-7 g SURF-7 s SURF-7 sp003599865 WGS QZKS01
d Bacteria p Desulfobacterota c Desulfobacteria o Desulfobacterales f SURF-3 g SURF-3 s SURF-3 sp003599885 WGS QZKW01
d Bacteria p Proteobacteria c Gammaproteobacteria o Nevskiales f Salinisphaeraceae g Salinisphaera A s Salinisphaera A halophila A WGS QJOD01
d Bacteria p Proteobacteria c Gammaproteobacteria o Nevskiales f Salinisphaeraceae g GCA-2683405 s GCA-2683405 sp002683405 WGS NYTH01
d Bacteria p Proteobacteria c Gammaproteobacteria o Nevskiales f Salinisphaeraceae g Salinisphaera s Salinisphaera orenii WGS AYKH01

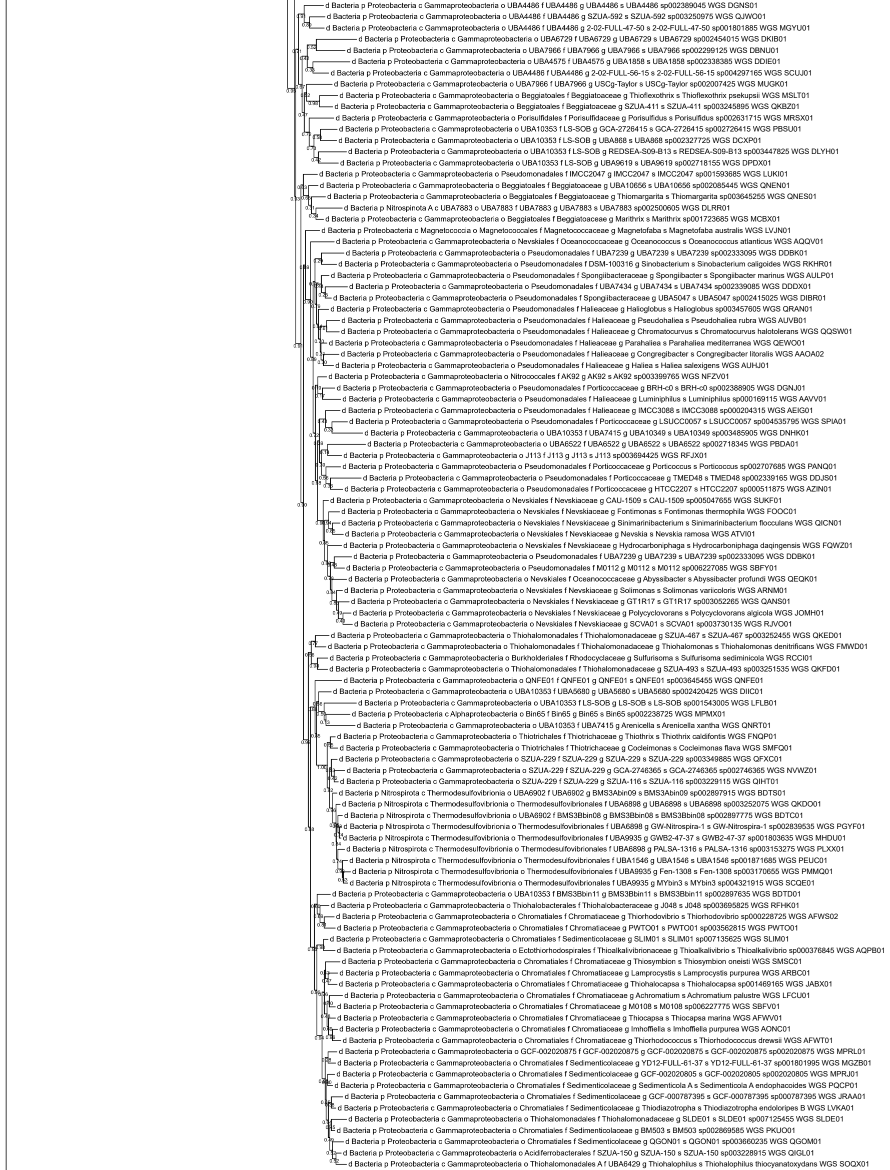
d Bacteria p Proteobacteria c Gammaproteobacteria o UBA4486 f UBA4486 g UBA4486 s UBA4486 sp002389045 WGS DGNS01
d Bacteria p Proteobacteria c Gammaproteobacteria o UBA4486 f UBA4486 g SZUA-592 s SZUA-592 sp003250975 WGS QJWO01
d Bacteria p Proteobacteria c Gammaproteobacteria o UBA4486 f UBA4486 g 2-02-FULL-47-50 s 2-02-FULL-47-50 sp001801885 WGS MGYU01
d Bacteria p Proteobacteria c Gammaproteobacteria o UBA6729 f UBA6729 g UBA6729 s UBA6729 sp002454015 WGS DKIB01
d Bacteria p Proteobacteria c Gammaproteobacteria o UBA7966 f UBA7966 g UBA7966 s UBA7966 sp002299125 WGS DBNU01
d Bacteria p Proteobacteria c Gammaproteobacteria o UBA4575 f UBA4575 g UBA1858 s UBA1858 sp002338385 WGS DDIE01
d Bacteria p Proteobacteria c Gammaproteobacteria o UBA4486 f UBA4486 g 2-02-FULL-56-15 s 2-02-FULL-56-15 sp004297165 WGS SCUJ01
d Bacteria p Proteobacteria c Gammaproteobacteria o UBA7966 f UBA7966 g USCg-Taylor s USCg-Taylor sp002007425 WGS MUGK01
d Bacteria p Proteobacteria c Gammaproteobacteria o Beggiatoales f Beggiatoaceae g Thioflexothrix s Thioflexothrix psekupsii WGS MSLT01
d Bacteria p Proteobacteria c Gammaproteobacteria o Beggiatoales f Beggiatoaceae g SZUA-411 s SZUA-411 sp003245895 WGS QKBZ01
d Bacteria p Proteobacteria c Gammaproteobacteria o Porisulfidales f Porisulfidaceae g Porisulfidus s Porisulfidus sp002631715 WGS MRSX01
d Bacteria p Proteobacteria c Gammaproteobacteria o UBA10353 f LS-SOB g GCA-2726415 s GCA-2726415 sp002726415 WGS PBSU01
d Bacteria p Proteobacteria c Gammaproteobacteria o UBA10353 f LS-SOB g UBA868 s UBA868 sp002327725 WGS DCXP01
d Bacteria p Proteobacteria c Gammaproteobacteria o UBA10353 f LS-SOB g REDSEA-S09-B13 s REDSEA-S09-B13 sp003447825 WGS DLYH01
d Bacteria p Proteobacteria c Gammaproteobacteria o UBA10353 f LS-SOB g UBA9619 s UBA9619 sp002718155 WGS DPDX01
d Bacteria p Proteobacteria c Gammaproteobacteria o Pseudomonadales f IMCC2047 g IMCC2047 s IMCC2047 sp001593685 WGS LUKI01
d Bacteria p Proteobacteria c Gammaproteobacteria o Beggiatoales f Beggiatoaceae g UBA10656 s UBA10656 sp002085445 WGS QNEN01
d Bacteria p Proteobacteria c Gammaproteobacteria o Beggiatoales f Beggiatoaceae g Thiomargarita s Thiomargarita sp003645255 WGS QNES01
d Bacteria p Nitrospinota A c UBA7883 o UBA7883 f UBA7883 g UBA7883 s UBA7883 sp002500605 WGS DLRR01
d Bacteria p Proteobacteria c Gammaproteobacteria o Beggiatoales f Beggiatoaceae g Marithrix s Marithrix sp001723685 WGS MCBX01
d Bacteria p Proteobacteria c Magnetococcia o Magnetococcales f Magnetococcaceae g Magnetofaba s Magnetofaba australis WGS LVJN01
d Bacteria p Proteobacteria c Gammaproteobacteria o Nevskiales f Oceanococcaceae g Oceanococcus s Oceanococcus atlanticus WGS AQQV01
d Bacteria p Proteobacteria c Gammaproteobacteria o Pseudomonadales f UBA7239 g UBA7239 s UBA7239 sp002333095 WGS DDBK01
d Bacteria p Proteobacteria c Gammaproteobacteria o Pseudomonadales f DSM-100316 g Sinobacterium s Sinobacterium caligoides WGS RKHR01
d Bacteria p Proteobacteria c Gammaproteobacteria o Pseudomonadales f Spongiibacteraceae g Spongiibacter s Spongiibacter marinus WGS AULP01
d Bacteria p Proteobacteria c Gammaproteobacteria o Pseudomonadales f UBA7434 g UBA7434 s UBA7434 sp002339085 WGS DDDX01
d Bacteria p Proteobacteria c Gammaproteobacteria o Pseudomonadales f Spongiibacteraceae g UBA5047 s UBA5047 sp002415025 WGS DIBR01
d Bacteria p Proteobacteria c Gammaproteobacteria o Pseudomonadales f Halieaceae g Halioglobus s Halioglobus sp003457605 WGS QRAN01
d Bacteria p Proteobacteria c Gammaproteobacteria o Pseudomonadales f Halieaceae g Pseudohaliea s Pseudohaliea rubra WGS AUVB01
d Bacteria p Proteobacteria c Gammaproteobacteria o Pseudomonadales f Halieaceae g Chromatocurvus s Chromatocurvus halotolerans WGS QQSW01
d Bacteria p Proteobacteria c Gammaproteobacteria o Pseudomonadales f Halieaceae g Parahaliea s Parahaliea mediterranea WGS QEWO01
d Bacteria p Proteobacteria c Gammaproteobacteria o Pseudomonadales f Halieaceae g Congregibacter s Congregibacter litoralis WGS AAOA02
d Bacteria p Proteobacteria c Gammaproteobacteria o Pseudomonadales f Halieaceae g Haliea s Haliea salexigens WGS AUHJ01
d Bacteria p Proteobacteria c Gammaproteobacteria o Nitrococcales f AK92 g AK92 s AK92 sp003399765 WGS NFZV01
d Bacteria p Proteobacteria c Gammaproteobacteria o Pseudomonadales f Porticoccaceae g BRH-c0 s BRH-c0 sp002388905 WGS DGNJ01
d Bacteria p Proteobacteria c Gammaproteobacteria o Pseudomonadales f Halieaceae g Luminiphilus s Luminiphilus sp000169115 WGS AAVV01
d Bacteria p Proteobacteria c Gammaproteobacteria o Pseudomonadales f Halieaceae g IMCC3088 s IMCC3088 sp000204315 WGS AEIG01
d Bacteria p Proteobacteria c Gammaproteobacteria o Pseudomonadales f Porticoccaceae g LSUCC0057 s LSUCC0057 sp004535795 WGS SPIA01
d Bacteria p Proteobacteria c Gammaproteobacteria o UBA10353 f UBA7415 g UBA10349 s UBA10349 sp003485905 WGS DNHK01
d Bacteria p Proteobacteria c Gammaproteobacteria o UBA6522 f UBA6522 g UBA6522 s UBA6522 sp002718345 WGS PBDA01
d Bacteria p Proteobacteria c Gammaproteobacteria o J113 f J113 g J113 s J113 sp003694425 WGS RFJX01
d Bacteria p Proteobacteria c Gammaproteobacteria o Pseudomonadales f Porticoccaceae g Porticoccus s Porticoccus sp002707685 WGS PANQ01
d Bacteria p Proteobacteria c Gammaproteobacteria o Pseudomonadales f Porticoccaceae g TMED48 s TMED48 sp002339165 WGS DDJS01
d Bacteria p Proteobacteria c Gammaproteobacteria o Pseudomonadales f Porticoccaceae g HTCC2207 s HTCC2207 sp000511875 WGS AZIN01
d Bacteria p Proteobacteria c Gammaproteobacteria o Nevskiales f Nevskiaceae g CAU-1509 s CAU-1509 sp005047655 WGS SUKF01
d Bacteria p Proteobacteria c Gammaproteobacteria o Nevskiales f Nevskiaceae g Fontimonas s Fontimonas thermophila WGS FOOC01
d Bacteria p Proteobacteria c Gammaproteobacteria o Nevskiales f Nevskiaceae g Sinimarinibacterium s Sinimarinibacterium flocculans WGS QICN01
d Bacteria p Proteobacteria c Gammaproteobacteria o Nevskiales f Nevskiaceae g Nevskia s Nevskia ramosa WGS ATVI01
d Bacteria p Proteobacteria c Gammaproteobacteria o Nevskiales f Nevskiaceae g Hydrocarboniphaga s Hydrocarboniphaga daqingensis WGS FQWZ01
d Bacteria p Proteobacteria c Gammaproteobacteria o Pseudomonadales f UBA7239 g UBA7239 s UBA7239 sp002333095 WGS DDBK01
d Bacteria p Proteobacteria c Gammaproteobacteria o Pseudomonadales f M0112 g M0112 s M0112 sp006227085 WGS SBFY01
d Bacteria p Proteobacteria c Gammaproteobacteria o Nevskiales f Oceanococcaceae g Abyssibacter s Abyssibacter profundi WGS QEQK01
d Bacteria p Proteobacteria c Gammaproteobacteria o Nevskiales f Nevskiaceae g Solimonas s Solimonas variicoloris WGS ARNM01
d Bacteria p Proteobacteria c Gammaproteobacteria o Nevskiales f Nevskiaceae g GT1R17 s GT1R17 sp003052265 WGS QANS01
d Bacteria p Proteobacteria c Gammaproteobacteria o Nevskiales f Nevskiaceae g Polycyclovorans s Polycyclovorans algicola WGS JOMH01
d Bacteria p Proteobacteria c Gammaproteobacteria o Nevskiales f Nevskiaceae g SCVA01 s SCVA01 sp003730135 WGS RJVO01
d Bacteria p Proteobacteria c Gammaproteobacteria o Thiohalomonadales f Thiohalomonadaceae g SZUA-467 s SZUA-467 sp003252455 WGS QKED01
d Bacteria p Proteobacteria c Gammaproteobacteria o Thiohalomonadales f Thiohalomonadaceae g Thiohalomonas s Thiohalomonas denitrificans WGS FMWD01
d Bacteria p Proteobacteria c Gammaproteobacteria o Burkholderiales f Rhodocyclaceae g Sulfurisoma s Sulfurisoma sediminicola WGS RCCI01
d Bacteria p Proteobacteria c Gammaproteobacteria o Thiohalomonadales f Thiohalomonadaceae g SZUA-493 s SZUA-493 sp003251535 WGS QKFD01
d Bacteria p Proteobacteria c Gammaproteobacteria o QNFE01 f QNFE01 g QNFE01 s QNFE01 sp003645455 WGS QNFE01
d Bacteria p Proteobacteria c Gammaproteobacteria o UBA10353 f UBA5680 g UBA5680 s UBA5680 sp002420425 WGS DIIC01
d Bacteria p Proteobacteria c Gammaproteobacteria o UBA10353 f LS-SOB g LS-SOB s LS-SOB sp001543005 WGS LFLB01
d Bacteria p Proteobacteria c Alphaproteobacteria o Bin65 f Bin65 g Bin65 s Bin65 sp002238725 WGS MPMX01
d Bacteria p Proteobacteria c Gammaproteobacteria o UBA10353 f UBA7415 g Arenicella s Arenicella xantha WGS QNRT01
d Bacteria p Proteobacteria c Gammaproteobacteria o Thiotrichales f Thiotrichaceae g Thiothrix s Thiothrix caldifontis WGS FNQP01
d Bacteria p Proteobacteria c Gammaproteobacteria o Thiotrichales f Thiotrichaceae g Cocleimonas s Cocleimonas flava WGS SMFQ01
d Bacteria p Proteobacteria c Gammaproteobacteria o SZUA-229 f SZUA-229 g SZUA-229 s SZUA-229 sp003349885 WGS QFXC01
d Bacteria p Proteobacteria c Gammaproteobacteria o SZUA-229 f SZUA-229 g GCA-2746365 s GCA-2746365 sp002746365 WGS NVWZ01
d Bacteria p Proteobacteria c Gammaproteobacteria o SZUA-229 f SZUA-229 g SZUA-116 s SZUA-116 sp003229115 WGS QIHT01
d Bacteria p Nitrospirota c Thermodesulfovibrionia o UBA6902 f UBA6902 g BMS3Abin09 s BMS3Abin09 sp002897915 WGS BDTS01
d Bacteria p Nitrospirota c Thermodesulfovibrionia o Thermodesulfovibrionales f UBA6898 g UBA6898 s UBA6898 sp003252075 WGS QKDO01
d Bacteria p Nitrospirota c Thermodesulfovibrionia o UBA6902 f BMS3Bbin08 g BMS3Bbin08 s BMS3Bbin08 sp002897775 WGS BDTC01
d Bacteria p Nitrospirota c Thermodesulfovibrionia o Thermodesulfovibrionales f UBA6898 g GW-Nitrospira-1 s GW-Nitrospira-1 sp002839535 WGS PGYF01
d Bacteria p Nitrospirota c Thermodesulfovibrionia o Thermodesulfovibrionales f UBA9935 g GWB2-47-37 s GWB2-47-37 sp001803635 WGS MHDU01
d Bacteria p Nitrospirota c Thermodesulfovibrionia o Thermodesulfovibrionales f UBA6898 g PALSA-1316 s PALSA-1316 sp003153275 WGS PLXX01
d Bacteria p Nitrospirota c Thermodesulfovibrionia o Thermodesulfovibrionales f UBA1546 g UBA1546 s UBA1546 sp001871685 WGS PEUC01
d Bacteria p Nitrospirota c Thermodesulfovibrionia o Thermodesulfovibrionales f UBA9935 g Fen-1308 s Fen-1308 sp003170655 WGS PMMQ01
d Bacteria p Nitrospirota c Thermodesulfovibrionia o Thermodesulfovibrionales f UBA9935 g MYbin3 s MYbin3 sp004321915 WGS SCQE01
d Bacteria p Proteobacteria c Gammaproteobacteria o UBA10353 f BMS3Bbin11 g BMS3Bbin11 s BMS3Bbin11 sp002897635 WGS BDTD01
d Bacteria p Proteobacteria c Gammaproteobacteria o Thiohalobacterales f Thiohalobacteraceae g J048 s J048 sp003695825 WGS RFHK01
d Bacteria p Proteobacteria c Gammaproteobacteria o Chromatiales f Chromatiaceae g Thiorhodovibrio s Thiorhodovibrio sp000228725 WGS AFWS02
d Bacteria p Proteobacteria c Gammaproteobacteria o Chromatiales f Chromatiaceae g PWTO01 s PWTO01 sp003562815 WGS PWTO01
d Bacteria p Proteobacteria c Gammaproteobacteria o Chromatiales f Sedimenticolaceae g SLIM01 s SLIM01 sp007135625 WGS SLIM01
d Bacteria p Proteobacteria c Gammaproteobacteria o Ectothiorhodospirales f Thioalkalivibrionaceae g Thioalkalivibrio s Thioalkalivibrio sp000376845 WGS AQPB01
d Bacteria p Proteobacteria c Gammaproteobacteria o Chromatiales f Chromatiaceae g Thiosymbion s Thiosymbion oneisti WGS SMSC01
d Bacteria p Proteobacteria c Gammaproteobacteria o Chromatiales f Chromatiaceae g Lamprocystis s Lamprocystis purpurea WGS ARBC01
d Bacteria p Proteobacteria c Gammaproteobacteria o Chromatiales f Chromatiaceae g Thiohalocapsa s Thiohalocapsa sp001469165 WGS JABX01
d Bacteria p Proteobacteria c Gammaproteobacteria o Chromatiales f Chromatiaceae g Achromatium s Achromatium palustre WGS LFCU01
d Bacteria p Proteobacteria c Gammaproteobacteria o Chromatiales f Chromatiaceae g M0108 s M0108 sp006227775 WGS SBFV01
d Bacteria p Proteobacteria c Gammaproteobacteria o Chromatiales f Chromatiaceae g Thiocapsa s Thiocapsa marina WGS AFWV01
d Bacteria p Proteobacteria c Gammaproteobacteria o Chromatiales f Chromatiaceae g Imhoffiella s Imhoffiella purpurea WGS AONC01
d Bacteria p Proteobacteria c Gammaproteobacteria o Chromatiales f Chromatiaceae g Thiorhodococcus s Thiorhodococcus drewsii WGS AFWT01
d Bacteria p Proteobacteria c Gammaproteobacteria o GCF-002020875 f GCF-002020875 g GCF-002020875 s GCF-002020875 sp002020875 WGS MPRL01
d Bacteria p Proteobacteria c Gammaproteobacteria o Chromatiales f Sedimenticolaceae g YD12-FULL-61-37 s YD12-FULL-61-37 sp001801995 WGS MGZB01
d Bacteria p Proteobacteria c Gammaproteobacteria o Chromatiales f Sedimenticolaceae g GCF-002020805 s GCF-002020805 sp002020805 WGS MPRJ01
d Bacteria p Proteobacteria c Gammaproteobacteria o Chromatiales f Sedimenticolaceae g Sedimenticola A s Sedimenticola A endophacoides WGS PQCP01
d Bacteria p Proteobacteria c Gammaproteobacteria o Chromatiales f Sedimenticolaceae g GCF-000787395 s GCF-000787395 sp000787395 WGS JRAA01
d Bacteria p Proteobacteria c Gammaproteobacteria o Chromatiales f Sedimenticolaceae g Thiodiazotropha s Thiodiazotropha endoloripes B WGS LVKA01
d Bacteria p Proteobacteria c Gammaproteobacteria o Thiohalomonadales f Thiohalomonadaceae g SLDE01 s SLDE01 sp007125455 WGS SLDE01
d Bacteria p Proteobacteria c Gammaproteobacteria o Chromatiales f Sedimenticolaceae g BM503 s BM503 sp002869585 WGS PKUO01
d Bacteria p Proteobacteria c Gammaproteobacteria o Chromatiales f Sedimenticolaceae g QGON01 s QGON01 sp003660235 WGS QGOM01
d Bacteria p Proteobacteria c Gammaproteobacteria o Acidiferrobacterales f SZUA-150 g SZUA-150 s SZUA-150 sp003228915 WGS QIGL01
d Bacteria p Proteobacteria c Gammaproteobacteria o Thiohalomonadales A f UBA6429 g Thiohalophilus s Thiohalophilus thiocyanatoxydans WGS SOQX01

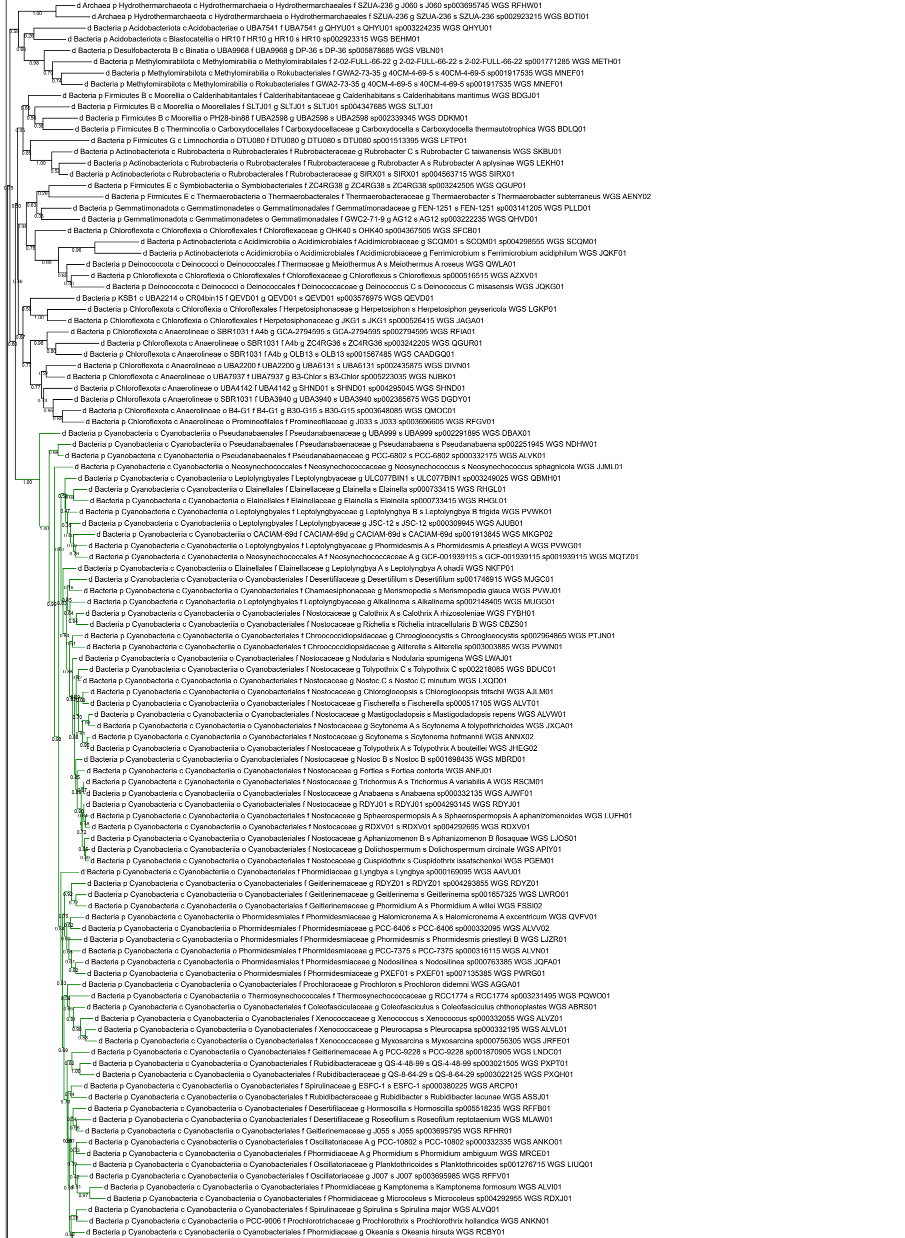
d Archaea p Hydrothermarchaeota c Hydrothermarchaeia o Hydrothermarchaeales f SZUA-236 g J060 s J060 sp003695745 WGS RFHW01
d Archaea p Hydrothermarchaeota c Hydrothermarchaeia o Hydrothermarchaeales f SZUA-236 g SZUA-236 s SZUA-236 sp002923215 WGS BDTI01
d Bacteria p Acidobacteriota c Acidobacteriae o UBA7541 f UBA7541 g QHYU01 s QHYU01 sp003224235 WGS QHYU01
d Bacteria p Acidobacteriota c Blastocatellia o HR10 f HR10 g HR10 s HR10 sp002923315 WGS BEHM01
d Bacteria p Desulfobacterota B c Binatia o UBA9968 f UBA9968 g DP-36 s DP-36 sp005878685 WGS VBLN01
d Bacteria p Methylomirabilota c Methylomirabilia o Methylomirabilales f 2-02-FULL-66-22 g 2-02-FULL-66-22 s 2-02-FULL-66-22 sp001771285 WGS METH01
d Bacteria p Methylomirabilota c Methylomirabilia o Rokubacteriales f GWA2-73-35 g 40CM-4-69-5 s 40CM-4-69-5 sp001917535 WGS MNEF01
d Bacteria p Methylomirabilota c Methylomirabilia o Rokubacteriales f GWA2-73-35 g 40CM-4-69-5 s 40CM-4-69-5 sp001917535 WGS MNEF01
d Bacteria p Firmicutes B c Moorellia o Calderihabitantales f Calderihabitantaceae g Calderihabitans s Calderihabitans maritimus WGS BDGJ01
d Bacteria p Firmicutes B c Moorellia o Moorellales f SLTJ01 g SLTJ01 s SLTJ01 sp004347685 WGS SLTJ01
d Bacteria p Firmicutes B c Moorellia o PH28-bin88 f UBA2598 g UBA2598 s UBA2598 sp002339345 WGS DDKM01
d Bacteria p Firmicutes B c Thermincolia o Carboxydocellales f Carboxydocellaceae g Carboxydocella s Carboxydocella thermautotrophica WGS BDLQ01
d Bacteria p Firmicutes G c Limnochordia o DTU080 f DTU080 g DTU080 s DTU080 sp001513395 WGS LFTP01
d Bacteria p Actinobacteriota c Rubrobacteria o Rubrobacterales f Rubrobacteraceae g Rubrobacter C s Rubrobacter C taiwanensis WGS SKBU01
d Bacteria p Actinobacteriota c Rubrobacteria o Rubrobacterales f Rubrobacteraceae g Rubrobacter A s Rubrobacter A aplysinae WGS LEKH01
d Bacteria p Actinobacteriota c Rubrobacteria o Rubrobacterales f Rubrobacteraceae g SIRX01 s SIRX01 sp004563715 WGS SIRX01
d Bacteria p Firmicutes E c Symbiobacteriia o Symbiobacteriales f ZC4RG38 g ZC4RG38 s ZC4RG38 sp003242505 WGS QGUP01
d Bacteria p Firmicutes E c Thermaerobacteria o Thermaerobacterales f Thermaerobacteraceae g Thermaerobacter s Thermaerobacter subterraneus WGS AENY02
d Bacteria p Gemmatimonadota c Gemmatimonadetes o Gemmatimonadales f Gemmatimonadaceae g FEN-1251 s FEN-1251 sp003141205 WGS PLLD01
d Bacteria p Gemmatimonadota c Gemmatimonadetes o Gemmatimonadales f GWC2-71-9 g AG12 s AG12 sp003222235 WGS QHVD01
d Bacteria p Chloroflexota c Chloroflexia o Chloroflexales f Chloroflexaceae g OHK40 s OHK40 sp004367505 WGS SFCB01
d Bacteria p Actinobacteriota c Acidimicrobiia o Acidimicrobiales f Acidimicrobiaceae g SCQM01 s SCQM01 sp004298555 WGS SCQM01
d Bacteria p Actinobacteriota c Acidimicrobiia o Acidimicrobiales f Acidimicrobiaceae g Ferrimicrobium s Ferrimicrobium acidiphilum WGS JQKF01
d Bacteria p Deinococcota c Deinococci o Deinococcales f Thermaceae g Meiothermus A s Meiothermus A roseus WGS QWLA01
d Bacteria p Chloroflexota c Chloroflexia o Chloroflexales f Chloroflexaceae g Chloroflexus s Chloroflexus sp000516515 WGS AZXV01
d Bacteria p Deinococcota c Deinococci o Deinococcales f Deinococcaceae g Deinococcus C s Deinococcus C misasensis WGS JQKG01
d Bacteria p KSB1 c UBA2214 o CR04bin15 f QEVD01 g QEVD01 s QEVD01 sp003576975 WGS QEVD01
d Bacteria p Chloroflexota c Chloroflexia o Chloroflexales f Herpetosiphonaceae g Herpetosiphon s Herpetosiphon geysericola WGS LGKP01
d Bacteria p Chloroflexota c Chloroflexia o Chloroflexales f Herpetosiphonaceae g JKG1 s JKG1 sp000526415 WGS JAGA01
d Bacteria p Chloroflexota c Anaerolineae o SBR1031 f A4b g GCA-2794595 s GCA-2794595 sp002794595 WGS RFIA01
d Bacteria p Chloroflexota c Anaerolineae o SBR1031 f A4b g ZC4RG36 s ZC4RG36 sp003242205 WGS QGUR01
d Bacteria p Chloroflexota c Anaerolineae o SBR1031 f A4b g OLB13 s OLB13 sp001567485 WGS CAADGQ01
d Bacteria p Chloroflexota c Anaerolineae o UBA2200 f UBA2200 g UBA6131 s UBA6131 sp002435875 WGS DIVN01
d Bacteria p Chloroflexota c Anaerolineae o UBA7937 f UBA7937 g B3-Chlor s B3-Chlor sp005223035 WGS NJBK01
d Bacteria p Chloroflexota c Anaerolineae o UBA4142 f UBA4142 g SHND01 s SHND01 sp004295045 WGS SHND01
d Bacteria p Chloroflexota c Anaerolineae o SBR1031 f UBA3940 g UBA3940 s UBA3940 sp002385675 WGS DGDY01
d Bacteria p Chloroflexota c Anaerolineae o B4-G1 f B4-G1 g B30-G15 s B30-G15 sp003648085 WGS QMOC01
d Bacteria p Chloroflexota c Anaerolineae o Promineofilales f Promineofilaceae g J033 s J033 sp003696605 WGS RFGV01
d Bacteria p Cyanobacteria c Cyanobacteriia o Pseudanabaenales f Pseudanabaenaceae g UBA999 s UBA999 sp002291895 WGS DBAX01
d Bacteria p Cyanobacteria c Cyanobacteriia o Pseudanabaenales f Pseudanabaenaceae g Pseudanabaena s Pseudanabaena sp002251945 WGS NDHW01
d Bacteria p Cyanobacteria c Cyanobacteriia o Pseudanabaenales f Pseudanabaenaceae g PCC-6802 s PCC-6802 sp000332175 WGS ALVK01
d Bacteria p Cyanobacteria c Cyanobacteriia o Neosynechococcales f Neosynechococcaceae g Neosynechococcus s Neosynechococcus sphagnicola WGS JJML01
d Bacteria p Cyanobacteria c Cyanobacteriia o Leptolyngbyales f Leptolyngbyaceae g ULC077BIN1 s ULC077BIN1 sp003249025 WGS QBMH01
d Bacteria p Cyanobacteria c Cyanobacteriia o Elainellales f Elainellaceae g Elainella s Elainella sp000733415 WGS RHGL01
d Bacteria p Cyanobacteria c Cyanobacteriia o Elainellales f Elainellaceae g Elainella s Elainella sp000733415 WGS RHGL01
d Bacteria p Cyanobacteria c Cyanobacteriia o Leptolyngbyales f Leptolyngbyaceae g Leptolyngbya B s Leptolyngbya B frigida WGS PVWK01
d Bacteria p Cyanobacteria c Cyanobacteriia o Leptolyngbyales f Leptolyngbyaceae g JSC-12 s JSC-12 sp000309945 WGS AJUB01
d Bacteria p Cyanobacteria c Cyanobacteriia o CACIAM-69d f CACIAM-69d g CACIAM-69d s CACIAM-69d sp001913845 WGS MKGP02
d Bacteria p Cyanobacteria c Cyanobacteriia o Leptolyngbyales f Leptolyngbyaceae g Phormidesmis A s Phormidesmis A priestleyi A WGS PVWG01
d Bacteria p Cyanobacteria c Cyanobacteriia o Neosynechococcales A f Neosynechococcaceae A g GCF-001939115 s GCF-001939115 sp001939115 WGS MQTZ01
d Bacteria p Cyanobacteria c Cyanobacteriia o Elainellales f Elainellaceae g Leptolyngbya A s Leptolyngbya A ohadii WGS NKFP01
d Bacteria p Cyanobacteria c Cyanobacteriia o Cyanobacteriales f Desertifilaceae g Desertifilum s Desertifilum sp001746915 WGS MJGC01
d Bacteria p Cyanobacteria c Cyanobacteriia o Cyanobacteriales f Chamaesiphonaceae g Merismopedia s Merismopedia glauca WGS PVWJ01
d Bacteria p Cyanobacteria c Cyanobacteriia o Leptolyngbyales f Leptolyngbyaceae g Alkalinema s Alkalinema sp002148405 WGS MUGG01
d Bacteria p Cyanobacteria c Cyanobacteriia o Cyanobacteriales f Nostocaceae g Calothrix A s Calothrix A rhizosoleniae WGS FYBH01
d Bacteria p Cyanobacteria c Cyanobacteriia o Cyanobacteriales f Nostocaceae g Richelia s Richelia intracellularis B WGS CBZS01
d Bacteria p Cyanobacteria c Cyanobacteriia o Cyanobacteriales f Chroococcidiopsidaceae g Chroogloeocystis s Chroogloeocystis sp002964865 WGS PTJN01
d Bacteria p Cyanobacteria c Cyanobacteriia o Cyanobacteriales f Chroococcidiopsidaceae g Aliterella s Aliterella sp003003885 WGS PVWN01
d Bacteria p Cyanobacteria c Cyanobacteriia o Cyanobacteriales f Nostocaceae g Nodularia s Nodularia spumigena WGS LWAJ01
d Bacteria p Cyanobacteria c Cyanobacteriia o Cyanobacteriales f Nostocaceae g Tolypothrix C s Tolypothrix C sp002218085 WGS BDUC01
d Bacteria p Cyanobacteria c Cyanobacteriia o Cyanobacteriales f Nostocaceae g Nostoc C s Nostoc C minutum WGS LXQD01
d Bacteria p Cyanobacteria c Cyanobacteriia o Cyanobacteriales f Nostocaceae g Chlorogloeopsis s Chlorogloeopsis fritschii WGS AJLM01
d Bacteria p Cyanobacteria c Cyanobacteriia o Cyanobacteriales f Nostocaceae g Fischerella s Fischerella sp000517105 WGS ALVT01
d Bacteria p Cyanobacteria c Cyanobacteriia o Cyanobacteriales f Nostocaceae g Mastigocladopsis s Mastigocladopsis repens WGS ALVW01
d Bacteria p Cyanobacteria c Cyanobacteriia o Cyanobacteriales f Nostocaceae g Scytonema A s Scytonema A tolypothrichoides WGS JXCA01
d Bacteria p Cyanobacteria c Cyanobacteriia o Cyanobacteriales f Nostocaceae g Scytonema s Scytonema hofmannii WGS ANNX02
d Bacteria p Cyanobacteria c Cyanobacteriia o Cyanobacteriales f Nostocaceae g Tolypothrix A s Tolypothrix A bouteillei WGS JHEG02
d Bacteria p Cyanobacteria c Cyanobacteriia o Cyanobacteriales f Nostocaceae g Nostoc B s Nostoc B sp001698435 WGS MBRD01
d Bacteria p Cyanobacteria c Cyanobacteriia o Cyanobacteriales f Nostocaceae g Fortiea s Fortiea contorta WGS ANFJ01
d Bacteria p Cyanobacteria c Cyanobacteriia o Cyanobacteriales f Nostocaceae g Trichormus A s Trichormus A variabilis A WGS RSCM01
d Bacteria p Cyanobacteria c Cyanobacteriia o Cyanobacteriales f Nostocaceae g Anabaena s Anabaena sp000332135 WGS AJWF01
d Bacteria p Cyanobacteria c Cyanobacteriia o Cyanobacteriales f Nostocaceae g RDYJ01 s RDYJ01 sp004293145 WGS RDYJ01
d Bacteria p Cyanobacteria c Cyanobacteriia o Cyanobacteriales f Nostocaceae g Sphaerospermopsis A s Sphaerospermopsis A aphanizomenoides WGS LUFH01
d Bacteria p Cyanobacteria c Cyanobacteriia o Cyanobacteriales f Nostocaceae g RDXV01 s RDXV01 sp004292695 WGS RDXV01
d Bacteria p Cyanobacteria c Cyanobacteriia o Cyanobacteriales f Nostocaceae g Aphanizomenon B s Aphanizomenon B flosaquae WGS LJOS01
d Bacteria p Cyanobacteria c Cyanobacteriia o Cyanobacteriales f Nostocaceae g Dolichospermum s Dolichospermum circinale WGS APIY01
d Bacteria p Cyanobacteria c Cyanobacteriia o Cyanobacteriales f Nostocaceae g Cuspidothrix s Cuspidothrix issatschenkoi WGS PGEM01
d Bacteria p Cyanobacteria c Cyanobacteriia o Cyanobacteriales f Phormidiaceae g Lyngbya s Lyngbya sp000169095 WGS AAVU01
d Bacteria p Cyanobacteria c Cyanobacteriia o Cyanobacteriales f Geitlerinemaceae g RDYZ01 s RDYZ01 sp004293855 WGS RDYZ01
d Bacteria p Cyanobacteria c Cyanobacteriia o Cyanobacteriales f Geitlerinemaceae g Geitlerinema s Geitlerinema sp001657325 WGS LWRO01
d Bacteria p Cyanobacteria c Cyanobacteriia o Cyanobacteriales f Geitlerinemaceae g Phormidium A s Phormidium A willei WGS FSSI02
d Bacteria p Cyanobacteria c Cyanobacteriia o Phormidesmiales f Phormidesmiaceae g Halomicronema A s Halomicronema A excentricum WGS QVFV01
d Bacteria p Cyanobacteria c Cyanobacteriia o Phormidesmiales f Phormidesmiaceae g PCC-6406 s PCC-6406 sp000332095 WGS ALVV02
d Bacteria p Cyanobacteria c Cyanobacteriia o Phormidesmiales f Phormidesmiaceae g Phormidesmis s Phormidesmis priestleyi B WGS LJZR01
d Bacteria p Cyanobacteria c Cyanobacteriia o Phormidesmiales f Phormidesmiaceae g PCC-7375 s PCC-7375 sp000316115 WGS ALVN01
d Bacteria p Cyanobacteria c Cyanobacteriia o Phormidesmiales f Phormidesmiaceae g Nodosilinea s Nodosilinea sp000763385 WGS JQFA01
d Bacteria p Cyanobacteria c Cyanobacteriia o Phormidesmiales f Phormidesmiaceae g PXEF01 s PXEF01 sp007135385 WGS PWRG01
d Bacteria p Cyanobacteria c Cyanobacteriia o Cyanobacteriales f Prochloraceae g Prochloron s Prochloron didemni WGS AGGA01
d Bacteria p Cyanobacteria c Cyanobacteriia o Thermosynechococcales f Thermosynechococcaceae g RCC1774 s RCC1774 sp003231495 WGS PQWO01
d Bacteria p Cyanobacteria c Cyanobacteriia o Cyanobacteriales f Coleofasciculaceae g Coleofasciculus s Coleofasciculus chthonoplastes WGS ABRS01
d Bacteria p Cyanobacteria c Cyanobacteriia o Cyanobacteriales f Xenococcaceae g Xenococcus s Xenococcus sp000332055 WGS ALVZ01
d Bacteria p Cyanobacteria c Cyanobacteriia o Cyanobacteriales f Xenococcaceae g Pleurocapsa s Pleurocapsa sp000332195 WGS ALVL01
d Bacteria p Cyanobacteria c Cyanobacteriia o Cyanobacteriales f Xenococcaceae g Myxosarcina s Myxosarcina sp000756305 WGS JRFE01
d Bacteria p Cyanobacteria c Cyanobacteriia o Cyanobacteriales f Geitlerinemaceae A g PCC-9228 s PCC-9228 sp001870905 WGS LNDC01
d Bacteria p Cyanobacteria c Cyanobacteriia o Cyanobacteriales f Rubidibacteraceae g QS-4-48-99 s QS-4-48-99 sp003021505 WGS PXPT01
d Bacteria p Cyanobacteria c Cyanobacteriia o Cyanobacteriales f Rubidibacteraceae g QS-8-64-29 s QS-8-64-29 sp003022125 WGS PXQH01
d Bacteria p Cyanobacteria c Cyanobacteriia o Cyanobacteriales f Spirulinaceae g ESFC-1 s ESFC-1 sp000380225 WGS ARCP01
d Bacteria p Cyanobacteria c Cyanobacteriia o Cyanobacteriales f Rubidibacteraceae g Rubidibacter s Rubidibacter lacunae WGS ASSJ01
d Bacteria p Cyanobacteria c Cyanobacteriia o Cyanobacteriales f Desertifilaceae g Hormoscilla s Hormoscilla sp005518235 WGS RFFB01
d Bacteria p Cyanobacteria c Cyanobacteriia o Cyanobacteriales f Desertifilaceae g Roseofilum s Roseofilum reptotaenium WGS MLAW01
d Bacteria p Cyanobacteria c Cyanobacteriia o Cyanobacteriales f Geitlerinemaceae g J055 s J055 sp003695795 WGS RFHR01
d Bacteria p Cyanobacteria c Cyanobacteriia o Cyanobacteriales f Oscillatoriaceae A g PCC-10802 s PCC-10802 sp000332335 WGS ANKO01
d Bacteria p Cyanobacteria c Cyanobacteriia o Cyanobacteriales f Phormidiaceae A g Phormidium s Phormidium ambiguum WGS MRCE01
d Bacteria p Cyanobacteria c Cyanobacteriia o Cyanobacteriales f Oscillatoriaceae g Planktothricoides s Planktothricoides sp001276715 WGS LIUQ01
d Bacteria p Cyanobacteria c Cyanobacteriia o Cyanobacteriales f Oscillatoriaceae g J007 s J007 sp003695985 WGS RFFV01
d Bacteria p Cyanobacteria c Cyanobacteriia o Cyanobacteriales f Phormidiaceae g Kamptonema s Kamptonema formosum WGS ALVI01
d Bacteria p Cyanobacteria c Cyanobacteriia o Cyanobacteriales f Phormidiaceae g Microcoleus s Microcoleus sp004292955 WGS RDXJ01
d Bacteria p Cyanobacteria c Cyanobacteriia o Cyanobacteriales f Spirulinaceae g Spirulina s Spirulina major WGS ALVQ01
d Bacteria p Cyanobacteria c Cyanobacteriia o PCC-9006 f Prochlorotrichaceae g Prochlorothrix s Prochlorothrix hollandica WGS ANKN01
d Bacteria p Cyanobacteria c Cyanobacteriia o Cyanobacteriales f Phormidiaceae g Okeania s Okeania hirsuta WGS RCBY01

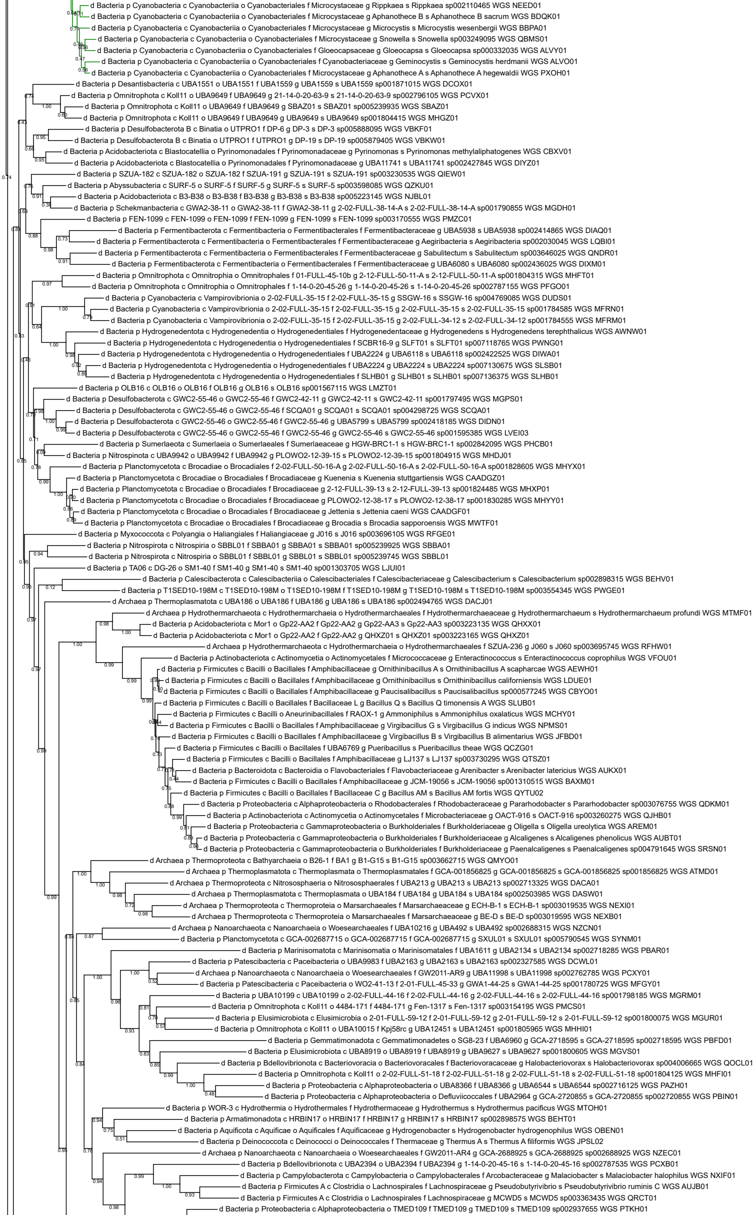
d Bacteria p Cyanobacteria c Cyanobacteriia o Cyanobacteriales f Microcystaceae g Rippkaea s Rippkaea sp002110465 WGS NEED01
d Bacteria p Cyanobacteria c Cyanobacteriia o Cyanobacteriales f Microcystaceae g Aphanothece B s Aphanothece B sacrum WGS BDQK01
d Bacteria p Cyanobacteria c Cyanobacteriia o Cyanobacteriales f Microcystaceae g Microcystis s Microcystis wesenbergii WGS BBPA01
d Bacteria p Cyanobacteria c Cyanobacteriia o Cyanobacteriales f Microcystaceae g Snowella s Snowella sp003249095 WGS QBMS01
d Bacteria p Cyanobacteria c Cyanobacteriia o Cyanobacteriales f Gloeocapsaceae g Gloeocapsa s Gloeocapsa sp000332035 WGS ALVY01
d Bacteria p Cyanobacteria c Cyanobacteriia o Cyanobacteriales f Cyanobacteriaceae g Geminocystis s Geminocystis herdmanii WGS ALVO01
d Bacteria p Cyanobacteria c Cyanobacteriia o Cyanobacteriales f Microcystaceae g Aphanothece A s Aphanothece A hegewaldii WGS PXOH01
d Bacteria p Desantisbacteria c UBA1551 o UBA1551 f UBA1559 g UBA1559 s UBA1559 sp001871015 WGS DCOX01
d Bacteria p Omnitrophota c Koll11 o UBA9649 f UBA9649 g 21-14-0-20-63-9 s 21-14-0-20-63-9 sp002796105 WGS PCVX01
d Bacteria p Omnitrophota c Koll11 o UBA9649 f UBA9649 g SBAZ01 s SBAZ01 sp005239935 WGS SBAZ01
d Bacteria p Omnitrophota c Koll11 o UBA9649 f UBA9649 g UBA9649 s UBA9649 sp001804415 WGS MHGZ01
d Bacteria p Desulfobacterota B c Binatia o UTPRO1 f DP-6 g DP-3 s DP-3 sp005888095 WGS VBKF01
d Bacteria p Desulfobacterota B c Binatia o UTPRO1 f UTPRO1 g DP-19 s DP-19 sp005879405 WGS VBKW01
d Bacteria p Acidobacteriota c Blastocatellia o Pyrinomonadales f Pyrinomonadaceae g Pyrinomonas s Pyrinomonas methylaliphatogenes WGS CBXV01
d Bacteria p Acidobacteriota c Blastocatellia o Pyrinomonadales f Pyrinomonadaceae g UBA11741 s UBA11741 sp002427845 WGS DIYZ01
d Bacteria p SZUA-182 c SZUA-182 o SZUA-182 f SZUA-191 g SZUA-191 s SZUA-191 sp003230535 WGS QIEW01
d Bacteria p Abyssubacteria c SURF-5 o SURF-5 f SURF-5 g SURF-5 s SURF-5 sp003598085 WGS QZKU01
d Bacteria p Acidobacteriota c B3-B38 o B3-B38 f B3-B38 g B3-B38 s B3-B38 sp005223145 WGS NJBL01
d Bacteria p Schekmanbacteria c GWA2-38-11 o GWA2-38-11 f GWA2-38-11 g 2-02-FULL-38-14-A s 2-02-FULL-38-14-A sp001790855 WGS MGDH01
d Bacteria p FEN-1099 c FEN-1099 o FEN-1099 f FEN-1099 g FEN-1099 s FEN-1099 sp003170555 WGS PMZC01
d Bacteria p Fermentibacterota c Fermentibacteria o Fermentibacterales f Fermentibacteraceae g UBA5938 s UBA5938 sp002414865 WGS DIAQ01
d Bacteria p Fermentibacterota c Fermentibacteria o Fermentibacterales f Fermentibacteraceae g Aegiribacteria s Aegiribacteria sp002030045 WGS LQBI01
d Bacteria p Fermentibacterota c Fermentibacteria o Fermentibacterales f Fermentibacteraceae g Sabulitectum s Sabulitectum sp003646025 WGS QNDR01
d Bacteria p Fermentibacterota c Fermentibacteria o Fermentibacterales f Fermentibacteraceae g UBA6080 s UBA6080 sp002436025 WGS DIXM01
d Bacteria p Omnitrophota c Omnitrophia o Omnitrophales f 01-FULL-45-10b g 2-12-FULL-50-11-A s 2-12-FULL-50-11-A sp001804315 WGS MHFT01
d Bacteria p Omnitrophota c Omnitrophia o Omnitrophales f 1-14-0-20-45-26 g 1-14-0-20-45-26 s 1-14-0-20-45-26 sp002787155 WGS PFGO01
d Bacteria p Cyanobacteria c Vampirovibrionia o 2-02-FULL-35-15 f 2-02-FULL-35-15 g SSGW-16 s SSGW-16 sp004769085 WGS DUDS01
d Bacteria p Cyanobacteria c Vampirovibrionia o 2-02-FULL-35-15 f 2-02-FULL-35-15 g 2-02-FULL-35-15 s 2-02-FULL-35-15 sp001784585 WGS MFRN01
d Bacteria p Cyanobacteria c Vampirovibrionia o 2-02-FULL-35-15 f 2-02-FULL-35-15 g 2-02-FULL-34-12 s 2-02-FULL-34-12 sp001784555 WGS MFRM01
d Bacteria p Hydrogenedentota c Hydrogenedentia o Hydrogenedentiales f Hydrogenedentaceae g Hydrogenedens s Hydrogenedens terephthalicus WGS AWNW01
d Bacteria p Hydrogenedentota c Hydrogenedentia o Hydrogenedentiales f SCBR16-9 g SLFT01 s SLFT01 sp007118765 WGS PWNG01
d Bacteria p Hydrogenedentota c Hydrogenedentia o Hydrogenedentiales f UBA2224 g UBA6118 s UBA6118 sp002422525 WGS DIWA01
d Bacteria p Hydrogenedentota c Hydrogenedentia o Hydrogenedentiales f UBA2224 g UBA2224 s UBA2224 sp007130675 WGS SLSB01
d Bacteria p Hydrogenedentota c Hydrogenedentia o Hydrogenedentiales f SLHB01 g SLHB01 s SLHB01 sp007136375 WGS SLHB01
d Bacteria p OLB16 c OLB16 o OLB16 f OLB16 g OLB16 s OLB16 sp001567115 WGS LMZT01
d Bacteria p Desulfobacterota c GWC2-55-46 o GWC2-55-46 f GWC2-42-11 g GWC2-42-11 s GWC2-42-11 sp001797495 WGS MGPS01
d Bacteria p Desulfobacterota c GWC2-55-46 o GWC2-55-46 f SCQA01 g SCQA01 s SCQA01 sp004298725 WGS SCQA01
d Bacteria p Desulfobacterota c GWC2-55-46 o GWC2-55-46 f GWC2-55-46 g UBA5799 s UBA5799 sp002418185 WGS DIDN01
d Bacteria p Desulfobacterota c GWC2-55-46 o GWC2-55-46 f GWC2-55-46 g GWC2-55-46 s GWC2-55-46 sp001595385 WGS LVEI03
d Bacteria p Sumerlaeota c Sumerlaeia o Sumerlaeales f Sumerlaeaceae g HGW-BRC1-1 s HGW-BRC1-1 sp002842095 WGS PHCB01
d Bacteria p Nitrospinota c UBA9942 o UBA9942 f UBA9942 g PLOWO2-12-39-15 s PLOWO2-12-39-15 sp001804915 WGS MHDJ01
d Bacteria p Planctomycetota c Brocadiae o Brocadiales f 2-02-FULL-50-16-A g 2-02-FULL-50-16-A s 2-02-FULL-50-16-A sp001828605 WGS MHYX01
d Bacteria p Planctomycetota c Brocadiae o Brocadiales f Brocadiaceae g Kuenenia s Kuenenia stuttgartiensis WGS CAADGZ01
d Bacteria p Planctomycetota c Brocadiae o Brocadiales f Brocadiaceae g 2-12-FULL-39-13 s 2-12-FULL-39-13 sp001824485 WGS MHXP01
d Bacteria p Planctomycetota c Brocadiae o Brocadiales f Brocadiaceae g PLOWO2-12-38-17 s PLOWO2-12-38-17 sp001830285 WGS MHYY01
d Bacteria p Planctomycetota c Brocadiae o Brocadiales f Brocadiaceae g Jettenia s Jettenia caeni WGS CAADGF01
d Bacteria p Planctomycetota c Brocadiae o Brocadiales f Brocadiaceae g Brocadia s Brocadia sapporoensis WGS MWTF01
d Bacteria p Myxococcota c Polyangia o Haliangiales f Haliangiaceae g J016 s J016 sp003696105 WGS RFGE01
d Bacteria p Nitrospirota c Nitrospiria o SBBL01 f SBBA01 g SBBA01 s SBBA01 sp005239925 WGS SBBA01
d Bacteria p Nitrospirota c Nitrospiria o SBBL01 f SBBL01 g SBBL01 s SBBL01 sp005239745 WGS SBBL01
d Bacteria p TA06 c DG-26 o SM1-40 f SM1-40 g SM1-40 s SM1-40 sp001303705 WGS LJUI01
d Bacteria p Calescibacterota c Calescibacteriia o Calescibacteriales f Calescibacteriaceae g Calescibacterium s Calescibacterium sp002898315 WGS BEHV01
d Bacteria p T1SED10-198M c T1SED10-198M o T1SED10-198M f T1SED10-198M g T1SED10-198M s T1SED10-198M sp003554345 WGS PWGE01
d Archaea p Thermoplasmatota c UBA186 o UBA186 f UBA186 g UBA186 s UBA186 sp002494765 WGS DACJ01
d Archaea p Hydrothermarchaeota c Hydrothermarchaeia o Hydrothermarchaeales f Hydrothermarchaeaceae g Hydrothermarchaeum s Hydrothermarchaeum profundi WGS MTMF01
d Bacteria p Acidobacteriota c Mor1 o Gp22-AA2 f Gp22-AA2 g Gp22-AA3 s Gp22-AA3 sp003223135 WGS QHXX01
d Bacteria p Acidobacteriota c Mor1 o Gp22-AA2 f Gp22-AA2 g QHXZ01 s QHXZ01 sp003223165 WGS QHXZ01
d Archaea p Hydrothermarchaeota c Hydrothermarchaeia o Hydrothermarchaeales f SZUA-236 g J060 s J060 sp003695745 WGS RFHW01
d Bacteria p Actinobacteriota c Actinomycetia o Actinomycetales f Micrococcaceae g Enteractinococcus s Enteractinococcus coprophilus WGS VFOU01
d Bacteria p Firmicutes c Bacilli o Bacillales f Amphibacillaceae g Ornithinibacillus A s Ornithinibacillus A scapharcae WGS AEWH01
d Bacteria p Firmicutes c Bacilli o Bacillales f Amphibacillaceae g Ornithinibacillus s Ornithinibacillus californiensis WGS LDUE01
d Bacteria p Firmicutes c Bacilli o Bacillales f Amphibacillaceae g Paucisalibacillus s Paucisalibacillus sp000577245 WGS CBYO01
d Bacteria p Firmicutes c Bacilli o Bacillales f Bacillaceae L g Bacillus Q s Bacillus Q timonensis A WGS SLUB01
d Bacteria p Firmicutes c Bacilli o Aneurinibacillales f RAOX-1 g Ammoniphilus s Ammoniphilus oxalaticus WGS MCHY01
d Bacteria p Firmicutes c Bacilli o Bacillales f Amphibacillaceae g Virgibacillus G s Virgibacillus G indicus WGS NPMS01
d Bacteria p Firmicutes c Bacilli o Bacillales f Amphibacillaceae g Virgibacillus B s Virgibacillus B alimentarius WGS JFBD01
d Bacteria p Firmicutes c Bacilli o Bacillales f UBA6769 g Pueribacillus s Pueribacillus theae WGS QCZG01
d Bacteria p Firmicutes c Bacilli o Bacillales f Amphibacillaceae g LJ137 s LJ137 sp003730295 WGS QTSZ01
d Bacteria p Bacteroidota c Bacteroidia o Flavobacteriales f Flavobacteriaceae g Arenibacter s Arenibacter latericius WGS AUKX01
d Bacteria p Firmicutes c Bacilli o Bacillales f Amphibacillaceae g JCM-19056 s JCM-19056 sp001310515 WGS BAXM01
d Bacteria p Firmicutes c Bacilli o Bacillales f Bacillaceae C g Bacillus AM s Bacillus AM fortis WGS QYTU02
d Bacteria p Proteobacteria c Alphaproteobacteria o Rhodobacterales f Rhodobacteraceae g Pararhodobacter s Pararhodobacter sp003076755 WGS QDKM01
d Bacteria p Actinobacteriota c Actinomycetia o Actinomycetales f Microbacteriaceae g OACT-916 s OACT-916 sp003260275 WGS QJHB01
d Bacteria p Proteobacteria c Gammaproteobacteria o Burkholderiales f Burkholderiaceae g Oligella s Oligella ureolytica WGS AREM01
d Bacteria p Proteobacteria c Gammaproteobacteria o Burkholderiales f Burkholderiaceae g Alcaligenes s Alcaligenes phenolicus WGS AUBT01
d Bacteria p Proteobacteria c Gammaproteobacteria o Burkholderiales f Burkholderiaceae g Paenalcaligenes s Paenalcaligenes sp004791645 WGS SRSN01
d Archaea p Thermoproteota c Bathyarchaeia o B26-1 f BA1 g B1-G15 s B1-G15 sp003662715 WGS QMYO01
d Archaea p Thermoplasmatota c Thermoplasmata o Thermoplasmatales f GCA-001856825 g GCA-001856825 s GCA-001856825 sp001856825 WGS ATMD01
d Archaea p Thermoproteota c Nitrososphaeria o Nitrososphaerales f UBA213 g UBA213 s UBA213 sp002713325 WGS DACA01
d Archaea p Thermoplasmatota c Thermoplasmata o UBA184 f UBA184 g UBA184 s UBA184 sp002503985 WGS DASW01
d Archaea p Thermoproteota c Thermoproteia o Marsarchaeales f Marsarchaeaceae g ECH-B-1 s ECH-B-1 sp003019535 WGS NEXI01
d Archaea p Thermoproteota c Thermoproteia o Marsarchaeales f Marsarchaeaceae g BE-D s BE-D sp003019595 WGS NEXB01
d Archaea p Nanoarchaeota c Nanoarchaeia o Woesearchaeales f UBA10216 g UBA492 s UBA492 sp002688315 WGS NZCN01
d Bacteria p Planctomycetota c GCA-002687715 o GCA-002687715 f GCA-002687715 g SXUL01 s SXUL01 sp005790545 WGS SYNM01
d Bacteria p Marinisomatota c Marinisomatia o Marinisomatales f UBA1611 g UBA2134 s UBA2134 sp002718285 WGS PBAR01
d Bacteria p Patescibacteria c Paceibacteria o UBA9983 f UBA2163 g UBA2163 s UBA2163 sp002327585 WGS DCWL01
d Archaea p Nanoarchaeota c Nanoarchaeia o Woesearchaeales f GW2011-AR9 g UBA11998 s UBA11998 sp002762785 WGS PCXY01
d Bacteria p Patescibacteria c Paceibacteria o WO2-41-13 f 2-01-FULL-45-33 g GWA1-44-25 s GWA1-44-25 sp001780725 WGS MFGY01
d Bacteria p UBA10199 c UBA10199 o 2-02-FULL-44-16 f 2-02-FULL-44-16 g 2-02-FULL-44-16 s 2-02-FULL-44-16 sp001798185 WGS MGRM01
d Bacteria p Omnitrophota c Koll11 o 4484-171 f 4484-171 g Fen-1317 s Fen-1317 sp003154195 WGS PMCS01
d Bacteria p Elusimicrobiota c Elusimicrobia o 2-01-FULL-59-12 f 2-01-FULL-59-12 g 2-01-FULL-59-12 s 2-01-FULL-59-12 sp001800075 WGS MGUR01
d Bacteria p Omnitrophota c Koll11 o UBA10015 f Kpj58rc g UBA12451 s UBA12451 sp001805965 WGS MHHI01
d Bacteria p Gemmatimonadota c Gemmatimonadetes o SG8-23 f UBA6960 g GCA-2718595 s GCA-2718595 sp002718595 WGS PBFD01
d Bacteria p Elusimicrobiota c UBA8919 o UBA8919 f UBA8919 g UBA9627 s UBA9627 sp001800605 WGS MGVS01
d Bacteria p Bdellovibrionota c Bacteriovoracia o Bacteriovoracales f Bacteriovoracaceae g Halobacteriovorax s Halobacteriovorax sp004006665 WGS QOCL01
d Bacteria p Omnitrophota c Koll11 o 2-02-FULL-51-18 f 2-02-FULL-51-18 g 2-02-FULL-51-18 s 2-02-FULL-51-18 sp001804125 WGS MHFI01
d Bacteria p Proteobacteria c Alphaproteobacteria o UBA8366 f UBA8366 g UBA6544 s UBA6544 sp002716125 WGS PAZH01
d Bacteria p Proteobacteria c Alphaproteobacteria o Defluviicoccales f UBA2964 g GCA-2720855 s GCA-2720855 sp002720855 WGS PBIN01
d Bacteria p WOR-3 c Hydrothermia o Hydrothermales f Hydrothermaceae g Hydrothermus s Hydrothermus pacificus WGS MTOH01
d Bacteria p Armatimonadota c HRBIN17 o HRBIN17 f HRBIN17 g HRBIN17 s HRBIN17 sp002898575 WGS BEHT01
d Bacteria p Aquificota c Aquificae o Aquificales f Aquificaceae g Hydrogenobacter s Hydrogenobacter hydrogenophilus WGS OBEN01
d Bacteria p Deinococcota c Deinococci o Deinococcales f Thermaceae g Thermus A s Thermus A filiformis WGS JPSL02
d Archaea p Nanoarchaeota c Nanoarchaeia o Woesearchaeales f GW2011-AR4 g GCA-2688925 s GCA-2688925 sp002688925 WGS NZEC01
d Bacteria p Bdellovibrionota c UBA2394 o UBA2394 f UBA2394 g 1-14-0-20-45-16 s 1-14-0-20-45-16 sp002787535 WGS PCXB01
d Bacteria p Campylobacterota c Campylobacteria o Campylobacterales f Arcobacteraceae g Malaciobacter s Malaciobacter halophilus WGS NXIF01
d Bacteria p Firmicutes A c Clostridia o Lachnospirales f Lachnospiraceae g Pseudobutyrivibrio s Pseudobutyrivibrio ruminis C WGS AUJB01
d Bacteria p Firmicutes A c Clostridia o Lachnospirales f Lachnospiraceae g MCWD5 s MCWD5 sp003363435 WGS QRCT01
d Bacteria p Proteobacteria c Alphaproteobacteria o TMED109 f TMED109 g TMED109 s TMED109 sp002937655 WGS PTKH01

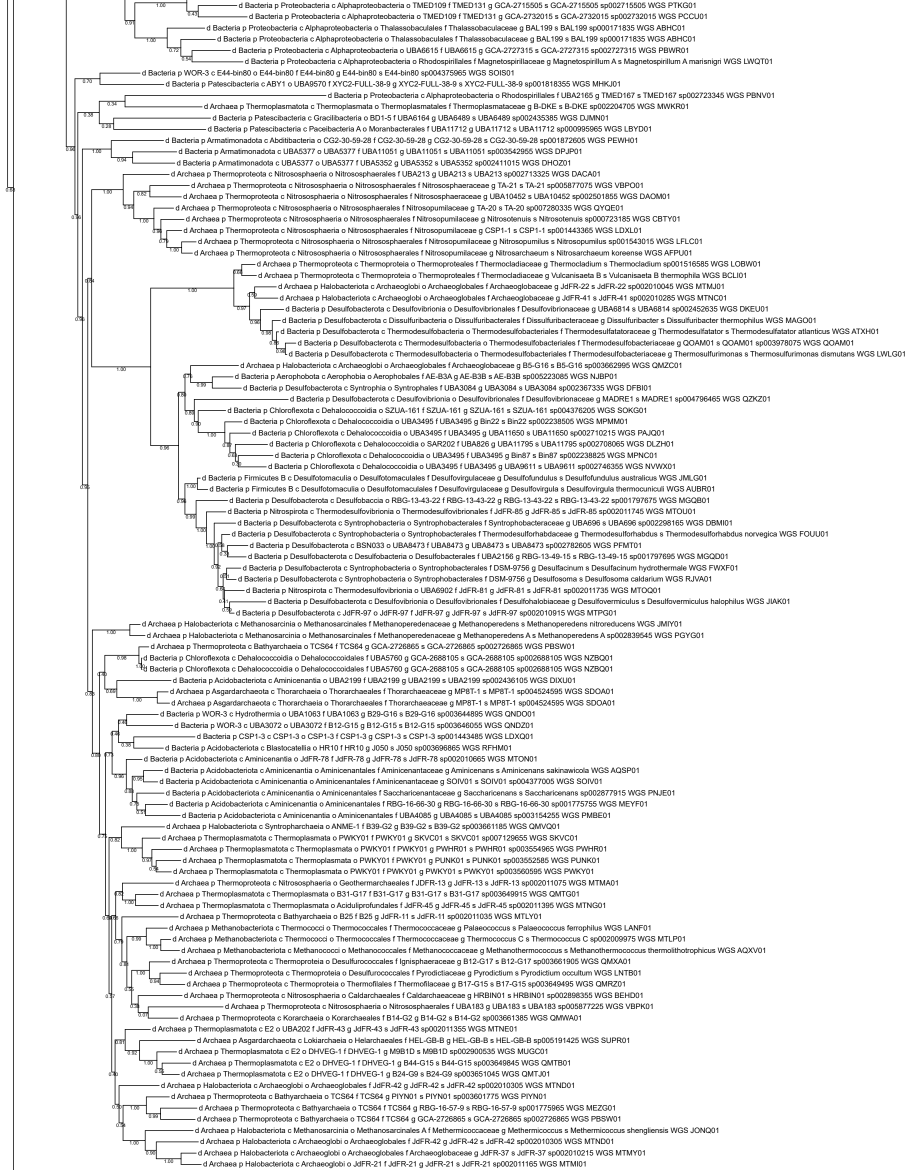
d Bacteria p Proteobacteria c Alphaproteobacteria o TMED109 f TMED131 g GCA-2715505 s GCA-2715505 sp002715505 WGS PTKG01
d Bacteria p Proteobacteria c Alphaproteobacteria o TMED109 f TMED131 g GCA-2732015 s GCA-2732015 sp002732015 WGS PCCU01
d Bacteria p Proteobacteria c Alphaproteobacteria o Thalassobaculales f Thalassobaculaceae g BAL199 s BAL199 sp000171835 WGS ABHC01
d Bacteria p Proteobacteria c Alphaproteobacteria o Thalassobaculales f Thalassobaculaceae g BAL199 s BAL199 sp000171835 WGS ABHC01
d Bacteria p Proteobacteria c Alphaproteobacteria o UBA6615 f UBA6615 g GCA-2727315 s GCA-2727315 sp002727315 WGS PBWR01
d Bacteria p Proteobacteria c Alphaproteobacteria o Rhodospirillales f Magnetospirillaceae g Magnetospirillum A s Magnetospirillum A marisnigri WGS LWQT01
d Bacteria p WOR-3 c E44-bin80 o E44-bin80 f E44-bin80 g E44-bin80 s E44-bin80 sp004375965 WGS SOIS01
d Bacteria p Patescibacteria c ABY1 o UBA9570 f XYC2-FULL-38-9 g XYC2-FULL-38-9 s XYC2-FULL-38-9 sp001818355 WGS MHKJ01
d Bacteria p Proteobacteria c Alphaproteobacteria o Rhodospirillales f UBA2165 g TMED167 s TMED167 sp002723345 WGS PBNV01
d Archaea p Thermoplasmatota c Thermoplasmata o Thermoplasmatales f Thermoplasmataceae g B-DKE s B-DKE sp002204705 WGS MWKR01
d Bacteria p Patescibacteria c Gracilibacteria o BD1-5 f UBA6164 g UBA6489 s UBA6489 sp002435385 WGS DJMN01
d Bacteria p Patescibacteria c Paceibacteria A o Moranbacterales f UBA11712 g UBA11712 s UBA11712 sp000995965 WGS LBYD01
d Bacteria p Armatimonadota c Abditibacteria o CG2-30-59-28 f CG2-30-59-28 g CG2-30-59-28 s CG2-30-59-28 sp001872605 WGS PEWH01
d Bacteria p Armatimonadota c UBA5377 o UBA5377 f UBA11051 g UBA11051 s UBA11051 sp003542955 WGS DPJP01
d Bacteria p Armatimonadota c UBA5377 o UBA5377 f UBA5352 g UBA5352 s UBA5352 sp002411015 WGS DHOZ01
d Archaea p Thermoproteota c Nitrososphaeria o Nitrososphaerales f UBA213 g UBA213 s UBA213 sp002713325 WGS DACA01
d Archaea p Thermoproteota c Nitrososphaeria o Nitrososphaerales f Nitrososphaeraceae g TA-21 s TA-21 sp005877075 WGS VBPO01
d Archaea p Thermoproteota c Nitrososphaeria o Nitrososphaerales f Nitrososphaeraceae g UBA10452 s UBA10452 sp002501855 WGS DAOM01
d Archaea p Thermoproteota c Nitrososphaeria o Nitrososphaerales f Nitrosopumilaceae g TA-20 s TA-20 sp007280335 WGS QYQE01
d Archaea p Thermoproteota c Nitrososphaeria o Nitrososphaerales f Nitrosopumilaceae g Nitrosotenuis s Nitrosotenuis sp000723185 WGS CBTY01
d Archaea p Thermoproteota c Nitrososphaeria o Nitrososphaerales f Nitrosopumilaceae g CSP1-1 s CSP1-1 sp001443365 WGS LDXL01
d Archaea p Thermoproteota c Nitrososphaeria o Nitrososphaerales f Nitrosopumilaceae g Nitrosopumilus s Nitrosopumilus sp001543015 WGS LFLC01
d Archaea p Thermoproteota c Nitrososphaeria o Nitrososphaerales f Nitrosopumilaceae g Nitrosarchaeum s Nitrosarchaeum koreense WGS AFPU01
d Archaea p Thermoproteota c Thermoproteia o Thermoproteales f Thermocladiaceae g Thermocladium s Thermocladium sp001516585 WGS LOBW01
d Archaea p Thermoproteota c Thermoproteia o Thermoproteales f Thermocladiaceae g Vulcanisaeta B s Vulcanisaeta B thermophila WGS BCLI01
d Archaea p Halobacteriota c Archaeoglobi o Archaeoglobales f Archaeoglobaceae g JdFR-22 s JdFR-22 sp002010045 WGS MTMJ01
d Archaea p Halobacteriota c Archaeoglobi o Archaeoglobales f Archaeoglobaceae g JdFR-41 s JdFR-41 sp002010285 WGS MTNC01
d Bacteria p Desulfobacterota c Desulfovibrionia o Desulfovibrionales f Desulfovibrionaceae g UBA6814 s UBA6814 sp002452635 WGS DKEU01
d Bacteria p Desulfobacterota c Dissulfuribacteria o Dissulfuribacterales f Dissulfuribacteraceae g Dissulfuribacter s Dissulfuribacter thermophilus WGS MAGO01
d Bacteria p Desulfobacterota c Thermodesulfobacteria o Thermodesulfobacteriales f Thermodesulfatatoraceae g Thermodesulfatator s Thermodesulfatator atlanticus WGS ATXH01
d Bacteria p Desulfobacterota c Thermodesulfobacteria o Thermodesulfobacteriales f Thermodesulfobacteriaceae g QOAM01 s QOAM01 sp003978075 WGS QOAM01
d Bacteria p Desulfobacterota c Thermodesulfobacteria o Thermodesulfobacteriales f Thermodesulfobacteriaceae g Thermosulfurimonas s Thermosulfurimonas dismutans WGS LWLG01
d Archaea p Halobacteriota c Archaeoglobi o Archaeoglobales f Archaeoglobaceae g B5-G16 s B5-G16 sp003662995 WGS QMZC01
d Bacteria p Aerophobota c Aerophobia o Aerophobales f AE-B3A g AE-B3B s AE-B3B sp005223085 WGS NJBP01
d Bacteria p Desulfobacterota c Syntrophia o Syntrophales f UBA3084 g UBA3084 s UBA3084 sp002367335 WGS DFBI01
d Bacteria p Desulfobacterota c Desulfovibrionia o Desulfovibrionales f Desulfovibrionaceae g MADRE1 s MADRE1 sp004796465 WGS QZKZ01
d Bacteria p Chloroflexota c Dehalococcoidia o SZUA-161 f SZUA-161 g SZUA-161 s SZUA-161 sp004376205 WGS SOKG01
d Bacteria p Chloroflexota c Dehalococcoidia o UBA3495 f UBA3495 g Bin22 s Bin22 sp002238505 WGS MPMM01
d Bacteria p Chloroflexota c Dehalococcoidia o UBA3495 f UBA3495 g UBA11650 s UBA11650 sp002710215 WGS PAJQ01
d Bacteria p Chloroflexota c Dehalococcoidia o SAR202 f UBA826 g UBA11795 s UBA11795 sp002708065 WGS DLZH01
d Bacteria p Chloroflexota c Dehalococcoidia o UBA3495 f UBA3495 g Bin87 s Bin87 sp002238825 WGS MPNC01
d Bacteria p Chloroflexota c Dehalococcoidia o UBA3495 f UBA3495 g UBA9611 s UBA9611 sp002746355 WGS NVWX01
d Bacteria p Firmicutes B c Desulfotomaculia o Desulfotomaculales f Desulfovirgulaceae g Desulfofundulus s Desulfofundulus australicus WGS JMLG01
d Bacteria p Firmicutes B c Desulfotomaculia o Desulfotomaculales f Desulfovirgulaceae g Desulfovirgula s Desulfovirgula thermocuniculi WGS AUBR01
d Bacteria p Desulfobacterota c Desulfobaccia o RBG-13-43-22 f RBG-13-43-22 g RBG-13-43-22 s RBG-13-43-22 sp001797675 WGS MGQB01
d Bacteria p Nitrospirota c Thermodesulfovibrionia o Thermodesulfovibrionales f JdFR-85 g JdFR-85 s JdFR-85 sp002011745 WGS MTOU01
d Bacteria p Desulfobacterota c Syntrophobacteria o Syntrophobacterales f Syntrophobacteraceae g UBA696 s UBA696 sp002298165 WGS DBMI01
d Bacteria p Desulfobacterota c Syntrophobacteria o Syntrophobacterales f Thermodesulforhabdaceae g Thermodesulforhabdus s Thermodesulforhabdus norvegica WGS FOUU01
d Bacteria p Desulfobacterota c BSN033 o UBA8473 f UBA8473 g UBA8473 s UBA8473 sp002782605 WGS PFMT01
d Bacteria p Desulfobacterota c Desulfobacteria o Desulfobacterales f UBA2156 g RBG-13-49-15 s RBG-13-49-15 sp001797695 WGS MGQD01
d Bacteria p Desulfobacterota c Syntrophobacteria o Syntrophobacterales f DSM-9756 g Desulfacinum s Desulfacinum hydrothermale WGS FWXF01
d Bacteria p Desulfobacterota c Syntrophobacteria o Syntrophobacterales f DSM-9756 g Desulfosoma s Desulfosoma caldarium WGS RJVA01
d Bacteria p Nitrospirota c Thermodesulfovibrionia o UBA6902 f JdFR-81 g JdFR-81 s JdFR-81 sp002011735 WGS MTOQ01
d Bacteria p Desulfobacterota c Desulfovibrionia o Desulfovibrionales f Desulfohalobiaceae g Desulfovermiculus s Desulfovermiculus halophilus WGS JIAK01
d Bacteria p Desulfobacterota c JdFR-97 o JdFR-97 f JdFR-97 g JdFR-97 s JdFR-97 sp002010915 WGS MTPG01
d Archaea p Halobacteriota c Methanosarcinia o Methanosarcinales f Methanoperedenaceae g Methanoperedens s Methanoperedens nitroreducens WGS JMIY01
d Archaea p Halobacteriota c Methanosarcinia o Methanosarcinales f Methanoperedenaceae g Methanoperedens A s Methanoperedens A sp002839545 WGS PGYG01
d Archaea p Thermoproteota c Bathyarchaeia o TCS64 f TCS64 g GCA-2726865 s GCA-2726865 sp002726865 WGS PBSW01
d Bacteria p Chloroflexota c Dehalococcoidia o Dehalococcoidales f UBA5760 g GCA-2688105 s GCA-2688105 sp002688105 WGS NZBQ01
d Bacteria p Chloroflexota c Dehalococcoidia o Dehalococcoidales f UBA5760 g GCA-2688105 s GCA-2688105 sp002688105 WGS NZBQ01
d Bacteria p Acidobacteriota c Aminicenantia o UBA2199 f UBA2199 g UBA2199 s UBA2199 sp002436105 WGS DIXU01
d Archaea p Asgardarchaeota c Thorarchaeia o Thorarchaeales f Thorarchaeaceae g MP8T-1 s MP8T-1 sp004524595 WGS SDOA01
d Archaea p Asgardarchaeota c Thorarchaeia o Thorarchaeales f Thorarchaeaceae g MP8T-1 s MP8T-1 sp004524595 WGS SDOA01
d Bacteria p WOR-3 c Hydrothermia o UBA1063 f UBA1063 g B29-G16 s B29-G16 sp003644895 WGS QNDO01
d Bacteria p WOR-3 c UBA3072 o UBA3072 f B12-G15 g B12-G15 s B12-G15 sp003646055 WGS QNDZ01
d Bacteria p CSP1-3 c CSP1-3 o CSP1-3 f CSP1-3 g CSP1-3 s CSP1-3 sp001443485 WGS LDXQ01
d Bacteria p Acidobacteriota c Blastocatellia o HR10 f HR10 g J050 s J050 sp003696865 WGS RFHM01
d Bacteria p Acidobacteriota c Aminicenantia o JdFR-78 f JdFR-78 g JdFR-78 s JdFR-78 sp002010665 WGS MTON01
d Bacteria p Acidobacteriota c Aminicenantia o Aminicenantales f Aminicenantaceae g Aminicenans s Aminicenans sakinawicola WGS AQSP01
d Bacteria p Acidobacteriota c Aminicenantia o Aminicenantales f Aminicenantaceae g SOIV01 s SOIV01 sp004377005 WGS SOIV01
d Bacteria p Acidobacteriota c Aminicenantia o Aminicenantales f Saccharicenantaceae g Saccharicenans s Saccharicenans sp002877915 WGS PNJE01
d Bacteria p Acidobacteriota c Aminicenantia o Aminicenantales f RBG-16-66-30 g RBG-16-66-30 s RBG-16-66-30 sp001775755 WGS MEYF01
d Bacteria p Acidobacteriota c Aminicenantia o Aminicenantales f UBA4085 g UBA4085 s UBA4085 sp003154255 WGS PMBE01
d Archaea p Halobacteriota c Syntropharchaeia o ANME-1 f B39-G2 g B39-G2 s B39-G2 sp003661185 WGS QMVQ01
d Archaea p Thermoplasmatota c Thermoplasmata o PWKY01 f PWKY01 g SKVC01 s SKVC01 sp007129655 WGS SKVC01
d Archaea p Thermoplasmatota c Thermoplasmata o PWKY01 f PWKY01 g PWHR01 s PWHR01 sp003554965 WGS PWHR01
d Archaea p Thermoplasmatota c Thermoplasmata o PWKY01 f PWKY01 g PUNK01 s PUNK01 sp003552585 WGS PUNK01
d Archaea p Thermoplasmatota c Thermoplasmata o PWKY01 f PWKY01 g PWKY01 s PWKY01 sp003560595 WGS PWKY01
d Archaea p Thermoproteota c Nitrososphaeria o Geothermarchaeales f JDFR-13 g JdFR-13 s JdFR-13 sp002011075 WGS MTMA01
d Archaea p Thermoplasmatota c Thermoplasmata o B31-G17 f B31-G17 g B31-G17 s B31-G17 sp003649915 WGS QMTG01
d Archaea p Thermoplasmatota c Thermoplasmata o Aciduliprofundales f JdFR-45 g JdFR-45 s JdFR-45 sp002011395 WGS MTNG01
d Archaea p Thermoproteota c Bathyarchaeia o B25 f B25 g JdFR-11 s JdFR-11 sp002011035 WGS MTLY01
d Archaea p Methanobacteriota c Thermococci o Thermococcales f Thermococcaceae g Palaeococcus s Palaeococcus ferrophilus WGS LANF01
d Archaea p Methanobacteriota c Thermococci o Thermococcales f Thermococcaceae g Thermococcus C s Thermococcus C sp002009975 WGS MTLP01
d Archaea p Methanobacteriota c Methanococci o Methanococcales f Methanococcaceae g Methanothermococcus s Methanothermococcus thermolithotrophicus WGS AQXV01
d Archaea p Thermoproteota c Thermoproteia o Desulfurococcales f Ignisphaeraceae g B12-G17 s B12-G17 sp003661905 WGS QMXA01
d Archaea p Thermoproteota c Thermoproteia o Desulfurococcales f Pyrodictiaceae g Pyrodictium s Pyrodictium occultum WGS LNTB01
d Archaea p Thermoproteota c Thermoproteia o Thermofilales f Thermofilaceae g B17-G15 s B17-G15 sp003649495 WGS QMRZ01
d Archaea p Thermoproteota c Nitrososphaeria o Caldarchaeales f Caldarchaeaceae g HRBIN01 s HRBIN01 sp002898355 WGS BEHD01
d Archaea p Thermoproteota c Nitrososphaeria o Nitrososphaerales f UBA183 g UBA183 s UBA183 sp005877225 WGS VBPK01
d Archaea p Thermoproteota c Korarchaeia o Korarchaeales f B14-G2 g B14-G2 s B14-G2 sp003661385 WGS QMWA01
d Archaea p Thermoplasmatota c E2 o UBA202 f JdFR-43 g JdFR-43 s JdFR-43 sp002011355 WGS MTNE01
d Archaea p Asgardarchaeota c Lokiarchaeia o Helarchaeales f HEL-GB-B g HEL-GB-B s HEL-GB-B sp005191425 WGS SUPR01
d Archaea p Thermoplasmatota c E2 o DHVEG-1 f DHVEG-1 g M9B1D s M9B1D sp002900535 WGS MUGC01
d Archaea p Thermoplasmatota c E2 o DHVEG-1 f DHVEG-1 g B44-G15 s B44-G15 sp003649845 WGS QMTB01
d Archaea p Thermoplasmatota c E2 o DHVEG-1 f DHVEG-1 g B24-G9 s B24-G9 sp003651045 WGS QMTJ01
d Archaea p Halobacteriota c Archaeoglobi o Archaeoglobales f JdFR-42 g JdFR-42 s JdFR-42 sp002010305 WGS MTND01
d Archaea p Thermoproteota c Bathyarchaeia o TCS64 f TCS64 g PIYN01 s PIYN01 sp003601775 WGS PIYN01
d Archaea p Thermoproteota c Bathyarchaeia o TCS64 f TCS64 g RBG-16-57-9 s RBG-16-57-9 sp001775965 WGS MEZG01
d Archaea p Thermoproteota c Bathyarchaeia o TCS64 f TCS64 g GCA-2726865 s GCA-2726865 sp002726865 WGS PBSW01
d Archaea p Halobacteriota c Methanosarcinia o Methanosarcinales A f Methermicoccaceae g Methermicoccus s Methermicoccus shengliensis WGS JONQ01
d Archaea p Halobacteriota c Archaeoglobi o Archaeoglobales f JdFR-42 g JdFR-42 s JdFR-42 sp002010305 WGS MTND01
d Archaea p Halobacteriota c Archaeoglobi o Archaeoglobales f Archaeoglobaceae g JdFR-37 s JdFR-37 sp002010215 WGS MTMY01
d Archaea p Halobacteriota c Archaeoglobi o JdFR-21 f JdFR-21 g JdFR-21 s JdFR-21 sp002011165 WGS MTMI01

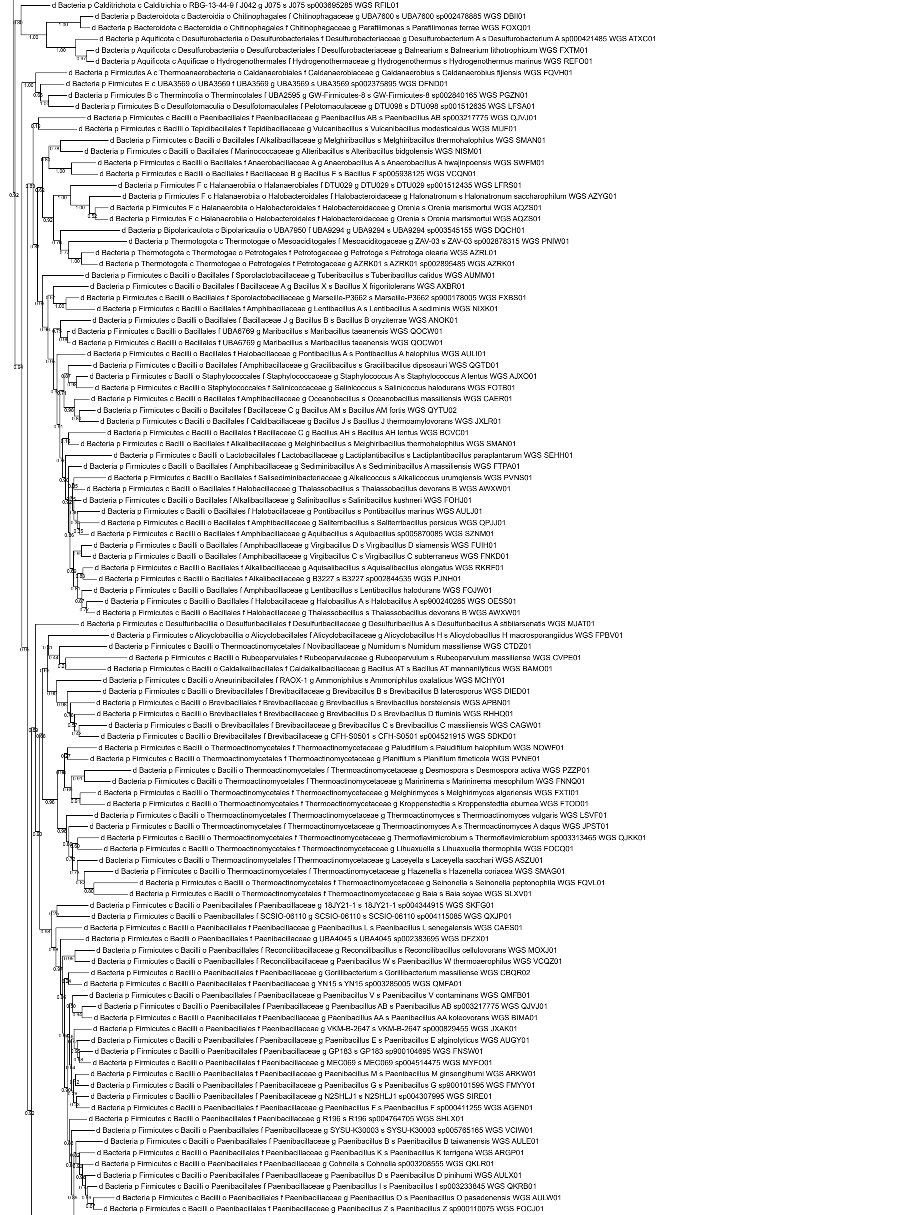
d Bacteria p Calditrichota c Calditrichia o RBG-13-44-9 f J042 g J075 s J075 sp003695285 WGS RFIL01
d Bacteria p Bacteroidota c Bacteroidia o Chitinophagales f Chitinophagaceae g UBA7600 s UBA7600 sp002478885 WGS DBII01
d Bacteria p Bacteroidota c Bacteroidia o Chitinophagales f Chitinophagaceae g Parafilimonas s Parafilimonas terrae WGS FOXQ01
d Bacteria p Aquificota c Desulfurobacteriia o Desulfurobacteriales f Desulfurobacteriaceae g Desulfurobacterium A s Desulfurobacterium A sp000421485 WGS ATXC01
d Bacteria p Aquificota c Desulfurobacteriia o Desulfurobacteriales f Desulfurobacteriaceae g Balnearium s Balnearium lithotrophicum WGS FXTM01
d Bacteria p Aquificota c Aquificae o Hydrogenothermales f Hydrogenothermaceae g Hydrogenothermus s Hydrogenothermus marinus WGS REFO01
d Bacteria p Firmicutes A c Thermoanaerobacteria o Caldanaerobiales f Caldanaerobiaceae g Caldanaerobius s Caldanaerobius fijiensis WGS FQVH01
d Bacteria p Firmicutes E c UBA3569 o UBA3569 f UBA3569 g UBA3569 s UBA3569 sp002375895 WGS DFND01
d Bacteria p Firmicutes B c Thermincolia o Thermincolales f UBA2595 g GW-Firmicutes-8 s GW-Firmicutes-8 sp002840165 WGS PGZN01
d Bacteria p Firmicutes B c Desulfotomaculia o Desulfotomaculales f Pelotomaculaceae g DTU098 s DTU098 sp001512635 WGS LFSA01
d Bacteria p Firmicutes c Bacilli o Paenibacillales f Paenibacillaceae g Paenibacillus AB s Paenibacillus AB sp003217775 WGS QJVJ01
d Bacteria p Firmicutes c Bacilli o Tepidibacillales f Tepidibacillaceae g Vulcanibacillus s Vulcanibacillus modesticaldus WGS MIJF01
d Bacteria p Firmicutes c Bacilli o Bacillales f Alkalibacillaceae g Melghiribacillus s Melghiribacillus thermohalophilus WGS SMAN01
d Bacteria p Firmicutes c Bacilli o Bacillales f Marinococcaceae g Alteribacillus s Alteribacillus bidgolensis WGS NISM01
d Bacteria p Firmicutes c Bacilli o Bacillales f Anaerobacillaceae A g Anaerobacillus A s Anaerobacillus A hwajinpoensis WGS SWFM01
d Bacteria p Firmicutes c Bacilli o Bacillales f Bacillaceae B g Bacillus F s Bacillus F sp005938125 WGS VCQN01
d Bacteria p Firmicutes F c Halanaerobiia o Halanaerobiales f DTU029 g DTU029 s DTU029 sp001512435 WGS LFRS01
d Bacteria p Firmicutes F c Halanaerobiia o Halobacteroidales f Halobacteroidaceae g Halonatronum s Halonatronum saccharophilum WGS AZYG01
d Bacteria p Firmicutes F c Halanaerobiia o Halobacteroidales f Halobacteroidaceae g Orenia s Orenia marismortui WGS AQZS01
d Bacteria p Firmicutes F c Halanaerobiia o Halobacteroidales f Halobacteroidaceae g Orenia s Orenia marismortui WGS AQZS01
d Bacteria p Bipolaricaulota c Bipolaricaulia o UBA7950 f UBA9294 g UBA9294 s UBA9294 sp003545155 WGS DQCH01
d Bacteria p Thermotogota c Thermotogae o Mesoaciditogales f Mesoaciditogaceae g ZAV-03 s ZAV-03 sp002878315 WGS PNIW01
d Bacteria p Thermotogota c Thermotogae o Petrotogales f Petrotogaceae g Petrotoga s Petrotoga olearia WGS AZRL01
d Bacteria p Thermotogota c Thermotogae o Petrotogales f Petrotogaceae g AZRK01 s AZRK01 sp002895485 WGS AZRK01
d Bacteria p Firmicutes c Bacilli o Bacillales f Sporolactobacillaceae g Tuberibacillus s Tuberibacillus calidus WGS AUMM01
d Bacteria p Firmicutes c Bacilli o Bacillales f Bacillaceae A g Bacillus X s Bacillus X frigoritolerans WGS AXBR01
d Bacteria p Firmicutes c Bacilli o Bacillales f Sporolactobacillaceae g Marseille-P3662 s Marseille-P3662 sp900178005 WGS FXBS01
d Bacteria p Firmicutes c Bacilli o Bacillales f Amphibacillaceae g Lentibacillus A s Lentibacillus A sediminis WGS NIXK01
d Bacteria p Firmicutes c Bacilli o Bacillales f Bacillaceae J g Bacillus B s Bacillus B oryziterrae WGS ANOK01
d Bacteria p Firmicutes c Bacilli o Bacillales f UBA6769 g Maribacillus s Maribacillus taeanensis WGS QOCW01
d Bacteria p Firmicutes c Bacilli o Bacillales f UBA6769 g Maribacillus s Maribacillus taeanensis WGS QOCW01
d Bacteria p Firmicutes c Bacilli o Bacillales f Halobacillaceae g Pontibacillus A s Pontibacillus A halophilus WGS AULI01
d Bacteria p Firmicutes c Bacilli o Bacillales f Amphibacillaceae g Gracilibacillus s Gracilibacillus dipsosauri WGS QGTD01
d Bacteria p Firmicutes c Bacilli o Staphylococcales f Staphylococcaceae g Staphylococcus A s Staphylococcus A lentus WGS AJXO01
d Bacteria p Firmicutes c Bacilli o Staphylococcales f Salinicoccaceae g Salinicoccus s Salinicoccus halodurans WGS FOTB01
d Bacteria p Firmicutes c Bacilli o Bacillales f Amphibacillaceae g Oceanobacillus s Oceanobacillus massiliensis WGS CAER01
d Bacteria p Firmicutes c Bacilli o Bacillales f Bacillaceae C g Bacillus AM s Bacillus AM fortis WGS QYTU02
d Bacteria p Firmicutes c Bacilli o Bacillales f Caldibacillaceae g Bacillus J s Bacillus J thermoamylovorans WGS JXLR01
d Bacteria p Firmicutes c Bacilli o Bacillales f Bacillaceae C g Bacillus AH s Bacillus AH lentus WGS BCVC01
d Bacteria p Firmicutes c Bacilli o Bacillales f Alkalibacillaceae g Melghiribacillus s Melghiribacillus thermohalophilus WGS SMAN01
d Bacteria p Firmicutes c Bacilli o Lactobacillales f Lactobacillaceae g Lactiplantibacillus s Lactiplantibacillus paraplantarum WGS SEHH01
d Bacteria p Firmicutes c Bacilli o Bacillales f Amphibacillaceae g Sediminibacillus A s Sediminibacillus A massiliensis WGS FTPA01
d Bacteria p Firmicutes c Bacilli o Bacillales f Salisediminibacteriaceae g Alkalicoccus s Alkalicoccus urumqiensis WGS PVNS01
d Bacteria p Firmicutes c Bacilli o Bacillales f Halobacillaceae g Thalassobacillus s Thalassobacillus devorans B WGS AWXW01
d Bacteria p Firmicutes c Bacilli o Bacillales f Alkalibacillaceae g Salinibacillus s Salinibacillus kushneri WGS FOHJ01
d Bacteria p Firmicutes c Bacilli o Bacillales f Halobacillaceae g Pontibacillus s Pontibacillus marinus WGS AULJ01
d Bacteria p Firmicutes c Bacilli o Bacillales f Amphibacillaceae g Saliterribacillus s Saliterribacillus persicus WGS QPJJ01
d Bacteria p Firmicutes c Bacilli o Bacillales f Amphibacillaceae g Aquibacillus s Aquibacillus sp005870085 WGS SZNM01
d Bacteria p Firmicutes c Bacilli o Bacillales f Amphibacillaceae g Virgibacillus D s Virgibacillus D siamensis WGS FUIH01
d Bacteria p Firmicutes c Bacilli o Bacillales f Amphibacillaceae g Virgibacillus C s Virgibacillus C subterraneus WGS FNKD01
d Bacteria p Firmicutes c Bacilli o Bacillales f Alkalibacillaceae g Aquisalibacillus s Aquisalibacillus elongatus WGS RKRF01
d Bacteria p Firmicutes c Bacilli o Bacillales f Alkalibacillaceae g B3227 s B3227 sp002844535 WGS PJNH01
d Bacteria p Firmicutes c Bacilli o Bacillales f Amphibacillaceae g Lentibacillus s Lentibacillus halodurans WGS FOJW01
d Bacteria p Firmicutes c Bacilli o Bacillales f Halobacillaceae g Halobacillus A s Halobacillus A sp900240285 WGS OESS01
d Bacteria p Firmicutes c Bacilli o Bacillales f Halobacillaceae g Thalassobacillus s Thalassobacillus devorans B WGS AWXW01
d Bacteria p Firmicutes c Desulfuribacillia o Desulfuribacillales f Desulfuribacillaceae g Desulfuribacillus A s Desulfuribacillus A stibiiarsenatis WGS MJAT01
d Bacteria p Firmicutes c Alicyclobacillia o Alicyclobacillales f Alicyclobacillaceae g Alicyclobacillus H s Alicyclobacillus H macrosporangiidus WGS FPBV01
d Bacteria p Firmicutes c Bacilli o Thermoactinomycetales f Novibacillaceae g Numidum s Numidum massiliense WGS CTDZ01
d Bacteria p Firmicutes c Bacilli o Rubeoparvulales f Rubeoparvulaceae g Rubeoparvulum s Rubeoparvulum massiliense WGS CVPE01
d Bacteria p Firmicutes c Bacilli o Caldalkalibacillales f Caldalkalibacillaceae g Bacillus AT s Bacillus AT mannanilyticus WGS BAMO01
d Bacteria p Firmicutes c Bacilli o Aneurinibacillales f RAOX-1 g Ammoniphilus s Ammoniphilus oxalaticus WGS MCHY01
d Bacteria p Firmicutes c Bacilli o Brevibacillales f Brevibacillaceae g Brevibacillus B s Brevibacillus B laterosporus WGS DIED01
d Bacteria p Firmicutes c Bacilli o Brevibacillales f Brevibacillaceae g Brevibacillus s Brevibacillus borstelensis WGS APBN01
d Bacteria p Firmicutes c Bacilli o Brevibacillales f Brevibacillaceae g Brevibacillus D s Brevibacillus D fluminis WGS RHHQ01
d Bacteria p Firmicutes c Bacilli o Brevibacillales f Brevibacillaceae g Brevibacillus C s Brevibacillus C massiliensis WGS CAGW01
d Bacteria p Firmicutes c Bacilli o Brevibacillales f Brevibacillaceae g CFH-S0501 s CFH-S0501 sp004521915 WGS SDKD01
d Bacteria p Firmicutes c Bacilli o Thermoactinomycetales f Thermoactinomycetaceae g Paludifilum s Paludifilum halophilum WGS NOWF01
d Bacteria p Firmicutes c Bacilli o Thermoactinomycetales f Thermoactinomycetaceae g Planifilum s Planifilum fimeticola WGS PVNE01
d Bacteria p Firmicutes c Bacilli o Thermoactinomycetales f Thermoactinomycetaceae g Desmospora s Desmospora activa WGS PZZP01
d Bacteria p Firmicutes c Bacilli o Thermoactinomycetales f Thermoactinomycetaceae g Marininema s Marininema mesophilum WGS FNNQ01
d Bacteria p Firmicutes c Bacilli o Thermoactinomycetales f Thermoactinomycetaceae g Melghirimyces s Melghirimyces algeriensis WGS FXTI01
d Bacteria p Firmicutes c Bacilli o Thermoactinomycetales f Thermoactinomycetaceae g Kroppenstedtia s Kroppenstedtia eburnea WGS FTOD01
d Bacteria p Firmicutes c Bacilli o Thermoactinomycetales f Thermoactinomycetaceae g Thermoactinomyces s Thermoactinomyces vulgaris WGS LSVF01
d Bacteria p Firmicutes c Bacilli o Thermoactinomycetales f Thermoactinomycetaceae g Thermoactinomyces A s Thermoactinomyces A daqus WGS JPST01
d Bacteria p Firmicutes c Bacilli o Thermoactinomycetales f Thermoactinomycetaceae g Thermoflavimicrobium s Thermoflavimicrobium sp003313465 WGS QJKK01
d Bacteria p Firmicutes c Bacilli o Thermoactinomycetales f Thermoactinomycetaceae g Lihuaxuella s Lihuaxuella thermophila WGS FOCQ01
d Bacteria p Firmicutes c Bacilli o Thermoactinomycetales f Thermoactinomycetaceae g Laceyella s Laceyella sacchari WGS ASZU01
d Bacteria p Firmicutes c Bacilli o Thermoactinomycetales f Thermoactinomycetaceae g Hazenella s Hazenella coriacea WGS SMAG01
d Bacteria p Firmicutes c Bacilli o Thermoactinomycetales f Thermoactinomycetaceae g Seinonella s Seinonella peptonophila WGS FQVL01
d Bacteria p Firmicutes c Bacilli o Thermoactinomycetales f Thermoactinomycetaceae g Baia s Baia soyae WGS SLXV01
d Bacteria p Firmicutes c Bacilli o Paenibacillales f Paenibacillaceae g 18JY21-1 s 18JY21-1 sp004344915 WGS SKFG01
d Bacteria p Firmicutes c Bacilli o Paenibacillales f SCSIO-06110 g SCSIO-06110 s SCSIO-06110 sp004115085 WGS QXJP01
d Bacteria p Firmicutes c Bacilli o Paenibacillales f Paenibacillaceae g Paenibacillus L s Paenibacillus L senegalensis WGS CAES01
d Bacteria p Firmicutes c Bacilli o Paenibacillales f Paenibacillaceae g UBA4045 s UBA4045 sp002383695 WGS DFZX01
d Bacteria p Firmicutes c Bacilli o Paenibacillales f Reconcilibacillaceae g Reconcilibacillus s Reconcilibacillus cellulovorans WGS MOXJ01
d Bacteria p Firmicutes c Bacilli o Paenibacillales f Reconcilibacillaceae g Paenibacillus W s Paenibacillus W thermoaerophilus WGS VCQZ01
d Bacteria p Firmicutes c Bacilli o Paenibacillales f Paenibacillaceae g Gorillibacterium s Gorillibacterium massiliense WGS CBQR02
d Bacteria p Firmicutes c Bacilli o Paenibacillales f Paenibacillaceae g YN15 s YN15 sp003285005 WGS QMFA01
d Bacteria p Firmicutes c Bacilli o Paenibacillales f Paenibacillaceae g Paenibacillus V s Paenibacillus V contaminans WGS QMFB01
d Bacteria p Firmicutes c Bacilli o Paenibacillales f Paenibacillaceae g Paenibacillus AB s Paenibacillus AB sp003217775 WGS QJVJ01
d Bacteria p Firmicutes c Bacilli o Paenibacillales f Paenibacillaceae g Paenibacillus AA s Paenibacillus AA koleovorans WGS BIMA01
d Bacteria p Firmicutes c Bacilli o Paenibacillales f Paenibacillaceae g VKM-B-2647 s VKM-B-2647 sp000829455 WGS JXAK01
d Bacteria p Firmicutes c Bacilli o Paenibacillales f Paenibacillaceae g Paenibacillus E s Paenibacillus E alginolyticus WGS AUGY01
d Bacteria p Firmicutes c Bacilli o Paenibacillales f Paenibacillaceae g GP183 s GP183 sp900104695 WGS FNSW01
d Bacteria p Firmicutes c Bacilli o Paenibacillales f Paenibacillaceae g MEC069 s MEC069 sp004514475 WGS MYFO01
d Bacteria p Firmicutes c Bacilli o Paenibacillales f Paenibacillaceae g Paenibacillus M s Paenibacillus M ginsengihumi WGS ARKW01
d Bacteria p Firmicutes c Bacilli o Paenibacillales f Paenibacillaceae g Paenibacillus G s Paenibacillus G sp900101595 WGS FMYY01
d Bacteria p Firmicutes c Bacilli o Paenibacillales f Paenibacillaceae g N2SHLJ1 s N2SHLJ1 sp004307995 WGS SIRE01
d Bacteria p Firmicutes c Bacilli o Paenibacillales f Paenibacillaceae g Paenibacillus F s Paenibacillus F sp000411255 WGS AGEN01
d Bacteria p Firmicutes c Bacilli o Paenibacillales f Paenibacillaceae g R196 s R196 sp004764705 WGS SHLX01
d Bacteria p Firmicutes c Bacilli o Paenibacillales f Paenibacillaceae g SYSU-K30003 s SYSU-K30003 sp005765165 WGS VCIW01
d Bacteria p Firmicutes c Bacilli o Paenibacillales f Paenibacillaceae g Paenibacillus B s Paenibacillus B taiwanensis WGS AULE01
d Bacteria p Firmicutes c Bacilli o Paenibacillales f Paenibacillaceae g Paenibacillus K s Paenibacillus K terrigena WGS ARGP01
d Bacteria p Firmicutes c Bacilli o Paenibacillales f Paenibacillaceae g Cohnella s Cohnella sp003208555 WGS QKLR01
d Bacteria p Firmicutes c Bacilli o Paenibacillales f Paenibacillaceae g Paenibacillus D s Paenibacillus D pinihumi WGS AULX01
d Bacteria p Firmicutes c Bacilli o Paenibacillales f Paenibacillaceae g Paenibacillus I s Paenibacillus I sp003233845 WGS QKRB01
d Bacteria p Firmicutes c Bacilli o Paenibacillales f Paenibacillaceae g Paenibacillus O s Paenibacillus O pasadenensis WGS AULW01
d Bacteria p Firmicutes c Bacilli o Paenibacillales f Paenibacillaceae g Paenibacillus Z s Paenibacillus Z sp900110075 WGS FOCJ01

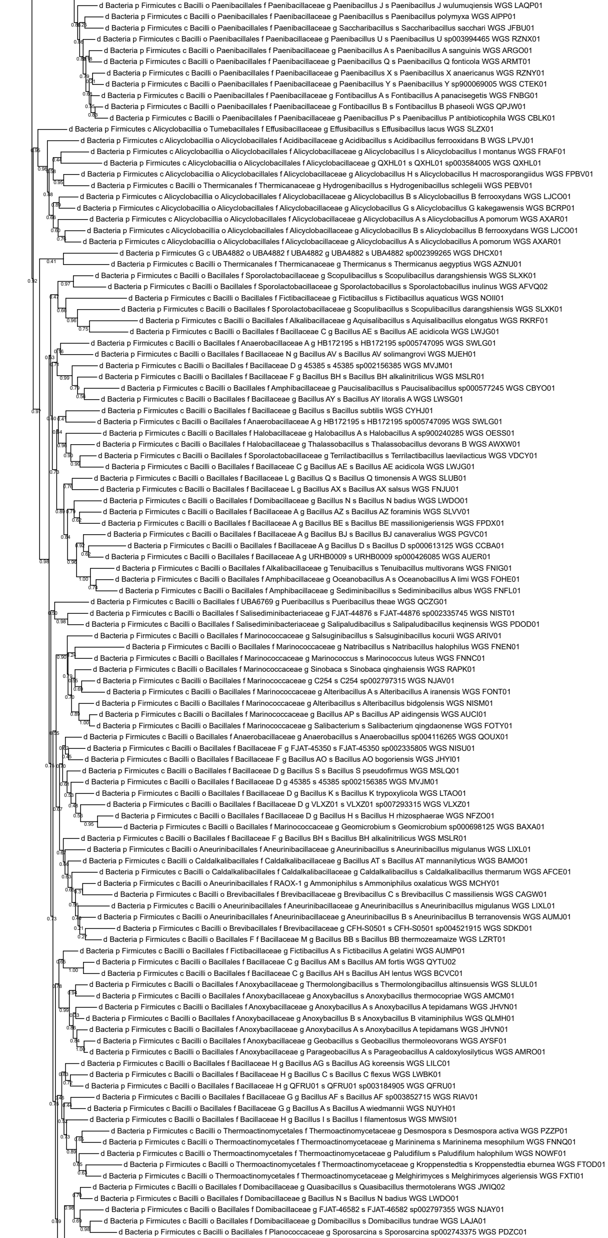
d Bacteria p Firmicutes c Bacilli o Paenibacillales f Paenibacillaceae g Paenibacillus J s Paenibacillus J wulumuqiensis WGS LAQP01
d Bacteria p Firmicutes c Bacilli o Paenibacillales f Paenibacillaceae g Paenibacillus s Paenibacillus polymyxa WGS AIPP01
d Bacteria p Firmicutes c Bacilli o Paenibacillales f Paenibacillaceae g Saccharibacillus s Saccharibacillus sacchari WGS JFBU01
d Bacteria p Firmicutes c Bacilli o Paenibacillales f Paenibacillaceae g Paenibacillus U s Paenibacillus U sp003994465 WGS RZNX01
d Bacteria p Firmicutes c Bacilli o Paenibacillales f Paenibacillaceae g Paenibacillus A s Paenibacillus A sanguinis WGS ARGO01
d Bacteria p Firmicutes c Bacilli o Paenibacillales f Paenibacillaceae g Paenibacillus Q s Paenibacillus Q fonticola WGS ARMT01
d Bacteria p Firmicutes c Bacilli o Paenibacillales f Paenibacillaceae g Paenibacillus X s Paenibacillus X anaericanus WGS RZNY01
d Bacteria p Firmicutes c Bacilli o Paenibacillales f Paenibacillaceae g Paenibacillus Y s Paenibacillus Y sp900069005 WGS CTEK01
d Bacteria p Firmicutes c Bacilli o Paenibacillales f Paenibacillaceae g Fontibacillus A s Fontibacillus A panacisegetis WGS FNBG01
d Bacteria p Firmicutes c Bacilli o Paenibacillales f Paenibacillaceae g Fontibacillus B s Fontibacillus B phaseoli WGS QPJW01
d Bacteria p Firmicutes c Bacilli o Paenibacillales f Paenibacillaceae g Paenibacillus P s Paenibacillus P antibioticophila WGS CBLK01
d Bacteria p Firmicutes c Alicyclobacillia o Tumebacillales f Effusibacillaceae g Effusibacillus s Effusibacillus lacus WGS SLZX01
d Bacteria p Firmicutes c Alicyclobacillia o Alicyclobacillales f Acidibacillaceae g Acidibacillus s Acidibacillus ferrooxidans B WGS LPVJ01
d Bacteria p Firmicutes c Alicyclobacillia o Alicyclobacillales f Alicyclobacillaceae g Alicyclobacillus I s Alicyclobacillus I montanus WGS FRAF01
d Bacteria p Firmicutes c Alicyclobacillia o Alicyclobacillales f Alicyclobacillaceae g QXHL01 s QXHL01 sp003584005 WGS QXHL01
d Bacteria p Firmicutes c Alicyclobacillia o Alicyclobacillales f Alicyclobacillaceae g Alicyclobacillus H s Alicyclobacillus H macrosporangiidus WGS FPBV01
d Bacteria p Firmicutes c Bacilli o Thermicanales f Thermicanaceae g Hydrogenibacillus s Hydrogenibacillus schlegelii WGS PEBV01
d Bacteria p Firmicutes c Alicyclobacillia o Alicyclobacillales f Alicyclobacillaceae g Alicyclobacillus B s Alicyclobacillus B ferrooxydans WGS LJCO01
d Bacteria p Firmicutes c Alicyclobacillia o Alicyclobacillales f Alicyclobacillaceae g Alicyclobacillus G s Alicyclobacillus G kakegawensis WGS BCRP01
d Bacteria p Firmicutes c Alicyclobacillia o Alicyclobacillales f Alicyclobacillaceae g Alicyclobacillus A s Alicyclobacillus A pomorum WGS AXAR01
d Bacteria p Firmicutes c Alicyclobacillia o Alicyclobacillales f Alicyclobacillaceae g Alicyclobacillus B s Alicyclobacillus B ferrooxydans WGS LJCO01
d Bacteria p Firmicutes c Alicyclobacillia o Alicyclobacillales f Alicyclobacillaceae g Alicyclobacillus A s Alicyclobacillus A pomorum WGS AXAR01
d Bacteria p Firmicutes G c UBA4882 o UBA4882 f UBA4882 g UBA4882 s UBA4882 sp002399265 WGS DHCX01
d Bacteria p Firmicutes c Bacilli o Thermicanales f Thermicanaceae g Thermicanus s Thermicanus aegyptius WGS AZNU01
d Bacteria p Firmicutes c Bacilli o Bacillales f Sporolactobacillaceae g Scopulibacillus s Scopulibacillus darangshiensis WGS SLXK01
d Bacteria p Firmicutes c Bacilli o Bacillales f Sporolactobacillaceae g Sporolactobacillus s Sporolactobacillus inulinus WGS AFVQ02
d Bacteria p Firmicutes c Bacilli o Bacillales f Fictibacillaceae g Fictibacillus s Fictibacillus aquaticus WGS NOII01
d Bacteria p Firmicutes c Bacilli o Bacillales f Sporolactobacillaceae g Scopulibacillus s Scopulibacillus darangshiensis WGS SLXK01
d Bacteria p Firmicutes c Bacilli o Bacillales f Alkalibacillaceae g Aquisalibacillus s Aquisalibacillus elongatus WGS RKRF01
d Bacteria p Firmicutes c Bacilli o Bacillales f Bacillaceae C g Bacillus AE s Bacillus AE acidicola WGS LWJG01
d Bacteria p Firmicutes c Bacilli o Bacillales f Anaerobacillaceae A g HB172195 s HB172195 sp005747095 WGS SWLG01
d Bacteria p Firmicutes c Bacilli o Bacillales f Bacillaceae N g Bacillus AV s Bacillus AV solimangrovi WGS MJEH01
d Bacteria p Firmicutes c Bacilli o Bacillales f Bacillaceae D g 45385 s 45385 sp002156385 WGS MVJM01
d Bacteria p Firmicutes c Bacilli o Bacillales f Bacillaceae F g Bacillus BH s Bacillus BH alkalinitrilicus WGS MSLR01
d Bacteria p Firmicutes c Bacilli o Bacillales f Amphibacillaceae g Paucisalibacillus s Paucisalibacillus sp000577245 WGS CBYO01
d Bacteria p Firmicutes c Bacilli o Bacillales f Bacillaceae g Bacillus AY s Bacillus AY litoralis A WGS LWSG01
d Bacteria p Firmicutes c Bacilli o Bacillales f Bacillaceae g Bacillus s Bacillus subtilis WGS CYHJ01
d Bacteria p Firmicutes c Bacilli o Bacillales f Anaerobacillaceae A g HB172195 s HB172195 sp005747095 WGS SWLG01
d Bacteria p Firmicutes c Bacilli o Bacillales f Halobacillaceae g Halobacillus A s Halobacillus A sp900240285 WGS OESS01
d Bacteria p Firmicutes c Bacilli o Bacillales f Halobacillaceae g Thalassobacillus s Thalassobacillus devorans B WGS AWXW01
d Bacteria p Firmicutes c Bacilli o Bacillales f Sporolactobacillaceae g Terrilactibacillus s Terrilactibacillus laevilacticus WGS VDCY01
d Bacteria p Firmicutes c Bacilli o Bacillales f Bacillaceae C g Bacillus AE s Bacillus AE acidicola WGS LWJG01
d Bacteria p Firmicutes c Bacilli o Bacillales f Bacillaceae L g Bacillus Q s Bacillus Q timonensis A WGS SLUB01
d Bacteria p Firmicutes c Bacilli o Bacillales f Bacillaceae L g Bacillus AX s Bacillus AX salsus WGS FNJU01
d Bacteria p Firmicutes c Bacilli o Bacillales f Domibacillaceae g Bacillus N s Bacillus N badius WGS LWDO01
d Bacteria p Firmicutes c Bacilli o Bacillales f Bacillaceae A g Bacillus AZ s Bacillus AZ foraminis WGS SLVV01
d Bacteria p Firmicutes c Bacilli o Bacillales f Bacillaceae A g Bacillus BE s Bacillus BE massilionigeriensis WGS FPDX01
d Bacteria p Firmicutes c Bacilli o Bacillales f Bacillaceae A g Bacillus BJ s Bacillus BJ canaveralius WGS PGVC01
d Bacteria p Firmicutes c Bacilli o Bacillales f Bacillaceae A g Bacillus D s Bacillus D sp000613125 WGS CCBA01
d Bacteria p Firmicutes c Bacilli o Bacillales f Bacillaceae A g URHB0009 s URHB0009 sp000426085 WGS AUER01
d Bacteria p Firmicutes c Bacilli o Bacillales f Alkalibacillaceae g Tenuibacillus s Tenuibacillus multivorans WGS FNIG01
d Bacteria p Firmicutes c Bacilli o Bacillales f Amphibacillaceae g Oceanobacillus A s Oceanobacillus A limi WGS FOHE01
d Bacteria p Firmicutes c Bacilli o Bacillales f Amphibacillaceae g Sediminibacillus s Sediminibacillus albus WGS FNFL01
d Bacteria p Firmicutes c Bacilli o Bacillales f UBA6769 g Pueribacillus s Pueribacillus theae WGS QCZG01
d Bacteria p Firmicutes c Bacilli o Bacillales f Salisediminibacteriaceae g FJAT-44876 s FJAT-44876 sp002335745 WGS NIST01
d Bacteria p Firmicutes c Bacilli o Bacillales f Salisediminibacteriaceae g Salipaludibacillus s Salipaludibacillus keqinensis WGS PDOD01
d Bacteria p Firmicutes c Bacilli o Bacillales f Marinococcaceae g Salsuginibacillus s Salsuginibacillus kocurii WGS ARIV01
d Bacteria p Firmicutes c Bacilli o Bacillales f Marinococcaceae g Natribacillus s Natribacillus halophilus WGS FNEN01
d Bacteria p Firmicutes c Bacilli o Bacillales f Marinococcaceae g Marinococcus s Marinococcus luteus WGS FNNC01
d Bacteria p Firmicutes c Bacilli o Bacillales f Marinococcaceae g Sinobaca s Sinobaca qinghaiensis WGS RAPK01
d Bacteria p Firmicutes c Bacilli o Bacillales f Marinococcaceae g C254 s C254 sp002797315 WGS NJAV01
d Bacteria p Firmicutes c Bacilli o Bacillales f Marinococcaceae g Alteribacillus A s Alteribacillus A iranensis WGS FONT01
d Bacteria p Firmicutes c Bacilli o Bacillales f Marinococcaceae g Alteribacillus s Alteribacillus bidgolensis WGS NISM01
d Bacteria p Firmicutes c Bacilli o Bacillales f Marinococcaceae g Bacillus AP s Bacillus AP aidingensis WGS AUCI01
d Bacteria p Firmicutes c Bacilli o Bacillales f Marinococcaceae g Salibacterium s Salibacterium qingdaonense WGS FOTY01
d Bacteria p Firmicutes c Bacilli o Bacillales f Anaerobacillaceae g Anaerobacillus s Anaerobacillus sp004116265 WGS QOUX01
d Bacteria p Firmicutes c Bacilli o Bacillales f Bacillaceae F g FJAT-45350 s FJAT-45350 sp002335805 WGS NISU01
d Bacteria p Firmicutes c Bacilli o Bacillales f Bacillaceae F g Bacillus AO s Bacillus AO bogoriensis WGS JHYI01
d Bacteria p Firmicutes c Bacilli o Bacillales f Bacillaceae D g Bacillus S s Bacillus S pseudofirmus WGS MSLQ01
d Bacteria p Firmicutes c Bacilli o Bacillales f Bacillaceae D g 45385 s 45385 sp002156385 WGS MVJM01
d Bacteria p Firmicutes c Bacilli o Bacillales f Bacillaceae D g Bacillus K s Bacillus K trypoxylicola WGS LTAO01
d Bacteria p Firmicutes c Bacilli o Bacillales f Bacillaceae D g VLXZ01 s VLXZ01 sp007293315 WGS VLXZ01
d Bacteria p Firmicutes c Bacilli o Bacillales f Bacillaceae D g Bacillus H s Bacillus H rhizosphaerae WGS NFZO01
d Bacteria p Firmicutes c Bacilli o Bacillales f Marinococcaceae g Geomicrobium s Geomicrobium sp000698125 WGS BAXA01
d Bacteria p Firmicutes c Bacilli o Bacillales f Bacillaceae F g Bacillus BH s Bacillus BH alkalinitrilicus WGS MSLR01
d Bacteria p Firmicutes c Bacilli o Aneurinibacillales f Aneurinibacillaceae g Aneurinibacillus s Aneurinibacillus migulanus WGS LIXL01
d Bacteria p Firmicutes c Bacilli o Caldalkalibacillales f Caldalkalibacillaceae g Bacillus AT s Bacillus AT mannanilyticus WGS BAMO01
d Bacteria p Firmicutes c Bacilli o Caldalkalibacillales f Caldalkalibacillaceae g Caldalkalibacillus s Caldalkalibacillus thermarum WGS AFCE01
d Bacteria p Firmicutes c Bacilli o Aneurinibacillales f RAOX-1 g Ammoniphilus s Ammoniphilus oxalaticus WGS MCHY01
d Bacteria p Firmicutes c Bacilli o Brevibacillales f Brevibacillaceae g Brevibacillus C s Brevibacillus C massiliensis WGS CAGW01
d Bacteria p Firmicutes c Bacilli o Aneurinibacillales f Aneurinibacillaceae g Aneurinibacillus s Aneurinibacillus migulanus WGS LIXL01
d Bacteria p Firmicutes c Bacilli o Aneurinibacillales f Aneurinibacillaceae g Aneurinibacillus B s Aneurinibacillus B terranovensis WGS AUMJ01
d Bacteria p Firmicutes c Bacilli o Brevibacillales f Brevibacillaceae g CFH-S0501 s CFH-S0501 sp004521915 WGS SDKD01
d Bacteria p Firmicutes c Bacilli o Bacillales F f Bacillaceae M g Bacillus BB s Bacillus BB thermozeamaize WGS LZRT01
d Bacteria p Firmicutes c Bacilli o Bacillales f Fictibacillaceae g Fictibacillus A s Fictibacillus A gelatini WGS AUMP01
d Bacteria p Firmicutes c Bacilli o Bacillales f Bacillaceae C g Bacillus AM s Bacillus AM fortis WGS QYTU02
d Bacteria p Firmicutes c Bacilli o Bacillales f Bacillaceae C g Bacillus AH s Bacillus AH lentus WGS BCVC01
d Bacteria p Firmicutes c Bacilli o Bacillales f Anoxybacillaceae g Thermolongibacillus s Thermolongibacillus altinsuensis WGS SLUL01
d Bacteria p Firmicutes c Bacilli o Bacillales f Anoxybacillaceae g Anoxybacillus s Anoxybacillus thermocopriae WGS AMCM01
d Bacteria p Firmicutes c Bacilli o Bacillales f Anoxybacillaceae g Anoxybacillus A s Anoxybacillus A tepidamans WGS JHVN01
d Bacteria p Firmicutes c Bacilli o Bacillales f Anoxybacillaceae g Anoxybacillus B s Anoxybacillus B vitaminiphilus WGS QLMH01
d Bacteria p Firmicutes c Bacilli o Bacillales f Anoxybacillaceae g Anoxybacillus A s Anoxybacillus A tepidamans WGS JHVN01
d Bacteria p Firmicutes c Bacilli o Bacillales f Anoxybacillaceae g Geobacillus s Geobacillus thermoleovorans WGS AYSF01
d Bacteria p Firmicutes c Bacilli o Bacillales f Anoxybacillaceae g Parageobacillus A s Parageobacillus A caldoxylosilyticus WGS AMRO01
d Bacteria p Firmicutes c Bacilli o Bacillales f Bacillaceae H g Bacillus AG s Bacillus AG koreensis WGS LILC01
d Bacteria p Firmicutes c Bacilli o Bacillales f Bacillaceae H g Bacillus C s Bacillus C flexus WGS LWBK01
d Bacteria p Firmicutes c Bacilli o Bacillales f Bacillaceae H g QFRU01 s QFRU01 sp003184905 WGS QFRU01
d Bacteria p Firmicutes c Bacilli o Bacillales f Bacillaceae G g Bacillus AF s Bacillus AF sp003852715 WGS RIAV01
d Bacteria p Firmicutes c Bacilli o Bacillales f Bacillaceae G g Bacillus A s Bacillus A wiedmannii WGS NUYH01
d Bacteria p Firmicutes c Bacilli o Bacillales f Bacillaceae H g Bacillus I s Bacillus I filamentosus WGS MWSI01
d Bacteria p Firmicutes c Bacilli o Thermoactinomycetales f Thermoactinomycetaceae g Desmospora s Desmospora activa WGS PZZP01
d Bacteria p Firmicutes c Bacilli o Thermoactinomycetales f Thermoactinomycetaceae g Marininema s Marininema mesophilum WGS FNNQ01
d Bacteria p Firmicutes c Bacilli o Thermoactinomycetales f Thermoactinomycetaceae g Paludifilum s Paludifilum halophilum WGS NOWF01
d Bacteria p Firmicutes c Bacilli o Thermoactinomycetales f Thermoactinomycetaceae g Kroppenstedtia s Kroppenstedtia eburnea WGS FTOD01
d Bacteria p Firmicutes c Bacilli o Thermoactinomycetales f Thermoactinomycetaceae g Melghirimyces s Melghirimyces algeriensis WGS FXTI01
d Bacteria p Firmicutes c Bacilli o Bacillales f Domibacillaceae g Quasibacillus s Quasibacillus thermotolerans WGS JWIQ02
d Bacteria p Firmicutes c Bacilli o Bacillales f Domibacillaceae g Bacillus N s Bacillus N badius WGS LWDO01
d Bacteria p Firmicutes c Bacilli o Bacillales f Domibacillaceae g FJAT-46582 s FJAT-46582 sp002797355 WGS NJAY01
d Bacteria p Firmicutes c Bacilli o Bacillales f Domibacillaceae g Domibacillus s Domibacillus tundrae WGS LAJA01
d Bacteria p Firmicutes c Bacilli o Bacillales f Planococcaceae g Sporosarcina s Sporosarcina sp002743375 WGS PDZC01

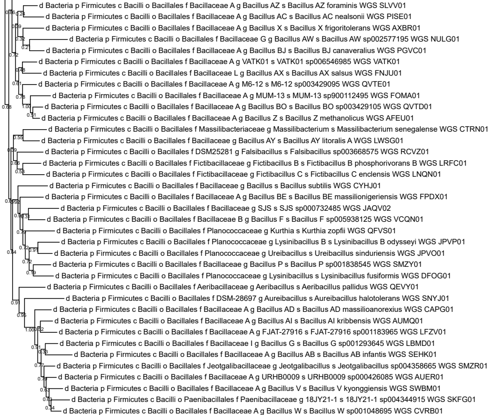
d Bacteria p Firmicutes c Bacilli o Bacillales f Bacillaceae A g Bacillus AZ s Bacillus AZ foraminis WGS SLVV01
d Bacteria p Firmicutes c Bacilli o Bacillales f Bacillaceae A g Bacillus AC s Bacillus AC nealsonii WGS PISE01
d Bacteria p Firmicutes c Bacilli o Bacillales f Bacillaceae A g Bacillus X s Bacillus X frigoritolerans WGS AXBR01
d Bacteria p Firmicutes c Bacilli o Bacillales f Bacillaceae G g Bacillus AW s Bacillus AW sp002577195 WGS NULG01
d Bacteria p Firmicutes c Bacilli o Bacillales f Bacillaceae A g Bacillus BJ s Bacillus BJ canaveralius WGS PGVC01
d Bacteria p Firmicutes c Bacilli o Bacillales f Bacillaceae A g VATK01 s VATK01 sp006546985 WGS VATK01
d Bacteria p Firmicutes c Bacilli o Bacillales f Bacillaceae L g Bacillus AX s Bacillus AX salsus WGS FNJU01
d Bacteria p Firmicutes c Bacilli o Bacillales f Bacillaceae A g M6-12 s M6-12 sp003429095 WGS QVTE01
d Bacteria p Firmicutes c Bacilli o Bacillales f Bacillaceae A g MUM-13 s MUM-13 sp900112495 WGS FOMA01
d Bacteria p Firmicutes c Bacilli o Bacillales f Bacillaceae A g Bacillus BO s Bacillus BO sp003429105 WGS QVTD01
d Bacteria p Firmicutes c Bacilli o Bacillales f Bacillaceae A g Bacillus Z s Bacillus Z methanolicus WGS AFEU01
d Bacteria p Firmicutes c Bacilli o Bacillales f Massilibacteriaceae g Massilibacterium s Massilibacterium senegalense WGS CTRN01
d Bacteria p Firmicutes c Bacilli o Bacillales f Bacillaceae g Bacillus AY s Bacillus AY litoralis A WGS LWSG01
d Bacteria p Firmicutes c Bacilli o Bacillales f DSM25281 g Falsibacillus s Falsibacillus sp003668575 WGS RCVZ01
d Bacteria p Firmicutes c Bacilli o Bacillales f Fictibacillaceae g Fictibacillus B s Fictibacillus B phosphorivorans B WGS LRFC01
d Bacteria p Firmicutes c Bacilli o Bacillales f Fictibacillaceae g Fictibacillus C s Fictibacillus C enclensis WGS LNQN01
d Bacteria p Firmicutes c Bacilli o Bacillales f Bacillaceae g Bacillus s Bacillus subtilis WGS CYHJ01
d Bacteria p Firmicutes c Bacilli o Bacillales f Bacillaceae A g Bacillus BE s Bacillus BE massilionigeriensis WGS FPDX01
d Bacteria p Firmicutes c Bacilli o Bacillales f Bacillaceae g SJS s SJS sp000732485 WGS JAQV02
d Bacteria p Firmicutes c Bacilli o Bacillales f Bacillaceae B g Bacillus F s Bacillus F sp005938125 WGS VCQN01
d Bacteria p Firmicutes c Bacilli o Bacillales f Planococcaceae g Kurthia s Kurthia zopfii WGS QFVS01
d Bacteria p Firmicutes c Bacilli o Bacillales f Planococcaceae g Lysinibacillus B s Lysinibacillus B odysseyi WGS JPVP01
d Bacteria p Firmicutes c Bacilli o Bacillales f Planococcaceae g Ureibacillus s Ureibacillus sinduriensis WGS JPVO01
d Bacteria p Firmicutes c Bacilli o Bacillales f Bacillaceae g Bacillus P s Bacillus P sp001838545 WGS SMZY01
d Bacteria p Firmicutes c Bacilli o Bacillales f Planococcaceae g Lysinibacillus s Lysinibacillus fusiformis WGS DFOG01
d Bacteria p Firmicutes c Bacilli o Bacillales f Aeribacillaceae g Aeribacillus s Aeribacillus pallidus WGS QEVY01
d Bacteria p Firmicutes c Bacilli o Bacillales f DSM-28697 g Aureibacillus s Aureibacillus halotolerans WGS SNYJ01
d Bacteria p Firmicutes c Bacilli o Bacillales f Bacillaceae A g Bacillus AD s Bacillus AD massilioanorexius WGS CAPG01
d Bacteria p Firmicutes c Bacilli o Bacillales f Bacillaceae A g Bacillus AI s Bacillus AI kribbensis WGS AUMQ01
d Bacteria p Firmicutes c Bacilli o Bacillales f Bacillaceae A g FJAT-27916 s FJAT-27916 sp001183965 WGS LFZV01
d Bacteria p Firmicutes c Bacilli o Bacillales f Bacillaceae I g Bacillus G s Bacillus G sp001293645 WGS LBMD01
d Bacteria p Firmicutes c Bacilli o Bacillales f Bacillaceae A g Bacillus AB s Bacillus AB infantis WGS SEHK01
d Bacteria p Firmicutes c Bacilli o Bacillales f Jeotgalibacillaceae g Jeotgalibacillus s Jeotgalibacillus sp004358665 WGS SMZR01
d Bacteria p Firmicutes c Bacilli o Bacillales f Bacillaceae A g URHB0009 s URHB0009 sp000426085 WGS AUER01
d Bacteria p Firmicutes c Bacilli o Bacillales f Bacillaceae A g Bacillus V s Bacillus V kyonggiensis WGS SWBM01
d Bacteria p Firmicutes c Bacilli o Paenibacillales f Paenibacillaceae g 18JY21-1 s 18JY21-1 sp004344915 WGS SKFG01
d Bacteria p Firmicutes c Bacilli o Bacillales f Bacillaceae A g Bacillus W s Bacillus W sp001048695 WGS CVRB01
